# Supplementary material for: A compendium of DNA-binding specificities of transcription factors in Pseudomonas syringae
Source: Nat Commun. 2020 Oct 2;11:4947. doi: 10.1038/s41467-020-18744-7 (PMC7532196; doi:10.1038/s41467-020-18744-7)
Supplement: Supplementary file 1 — Supplementary Information [file 41467_2020_18744_MOESM1_ESM.pdf]

Supplementary Information

The Compendium of DNA-Binding Specificities of Transcription Factors in *Pseudomonas syringae*

Fan et al.

Supplementary Information

Supplementary Figure 1. The schematic diagram of generating the input library for HT-SELEX, related to Fig. 1.

Supplementary Figure 2. Validation of different modes of TF binding, related to Fig. 2.

Supplementary Figure 3. Validation of PWM putative TF binding sites, related to Fig. 2.

Supplementary Table 1a. Family classification for TF based on DBD domain, related to Fig. 1.

Supplementary Table 1b. Strains, plasmids and primers used in this study, related to Fig. 1, Fig. 2.

Supplementary Table 1c. List of oligo sequences for building the HT-SELEX libraries used in the study, related to Fig. 1.

Supplementary Figure 1

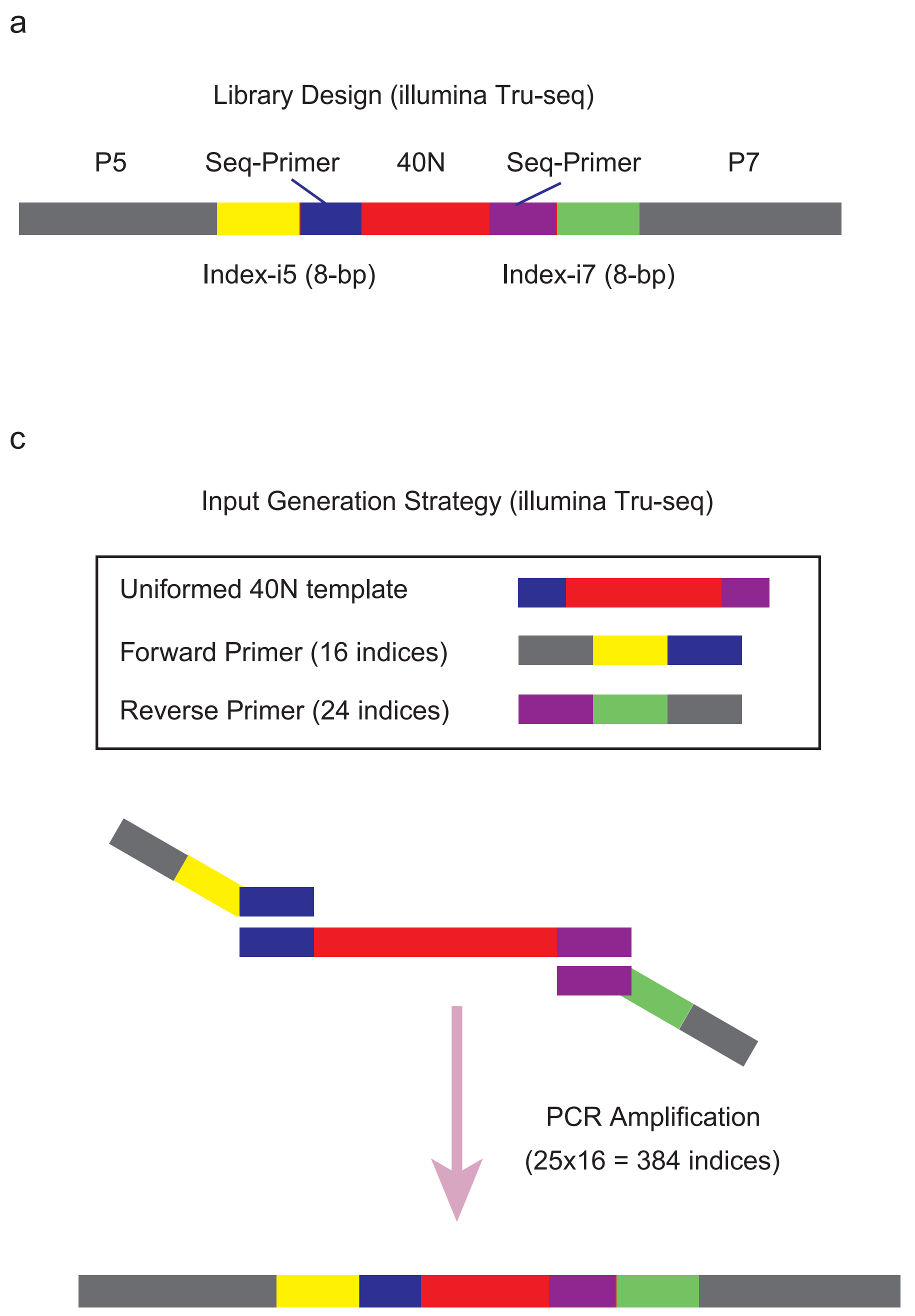

Supplementary figure legends

Supplementary Figure 1. The schematic diagram of generating the input library for HT-SELEX, related to Fig. 1.

(a) HT-SELEX input library design adapted to illumine Tru-seq system.

(b) HT-SELEX input library design adapted to BGIseq-2000 system.

(c) The strategy to assemble illumina Tru-seq input library with 384 indices.

(d) The strategy to assemble BGIseq-2000 input library with 96 indices.

Forward and reverse primer sequences are shown in Supplementary Table 1c (c-d).

Supplementary Figure 2

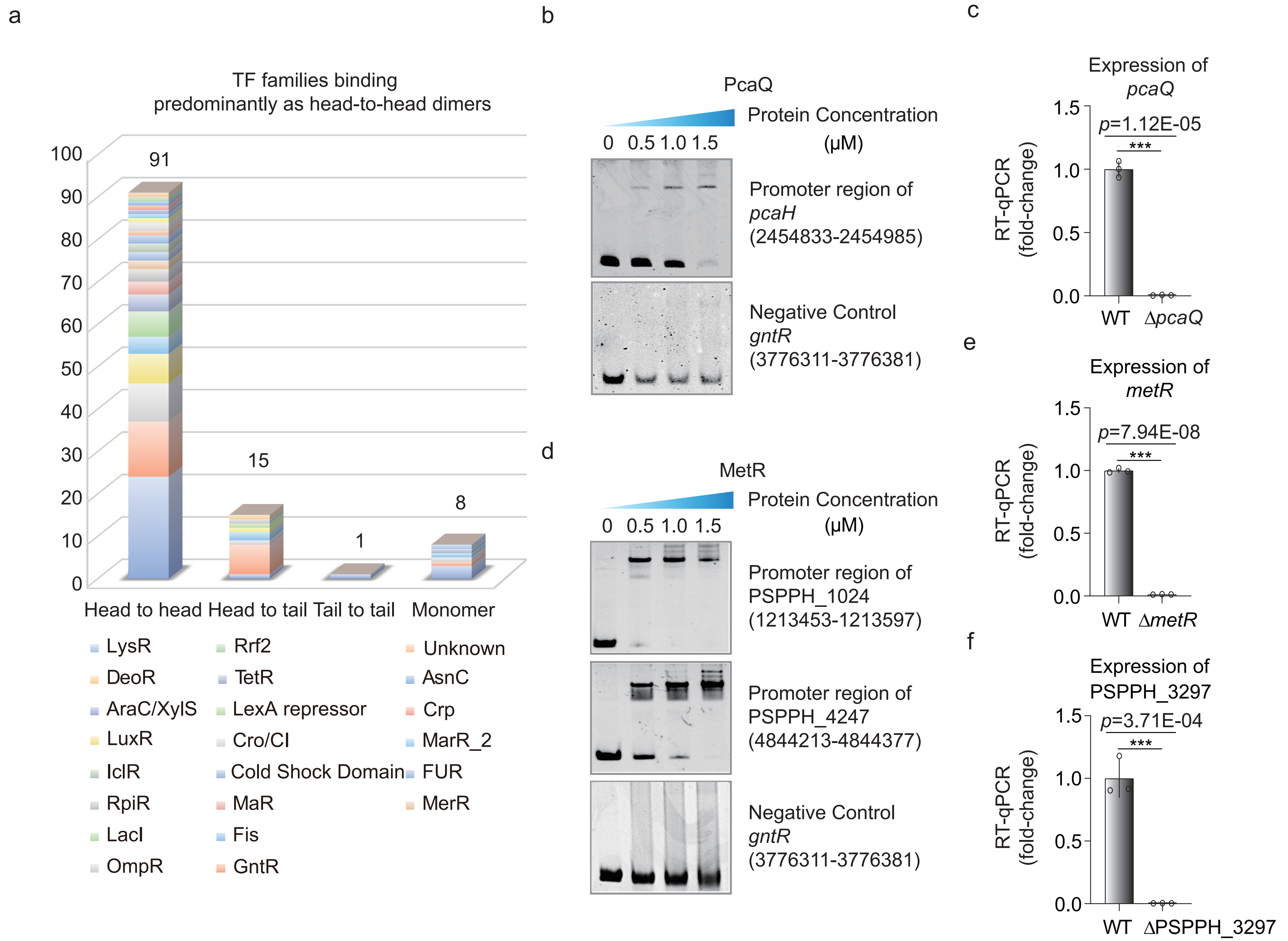

Supplementary Figure 2. Validation of different modes of TF binding, related to Fig. 2.

(a) The distribution of the number of TF binding modes different families. The TF family is color-coded. The prevalent dimeric types are classified as head-to-head, head-to-tail and tail-to-tail, most of them are the head-to-head palindromic binding preference.

(b) Validation of the putative binding of PcaQ in the promoter of the gene *pcaH* was verified using EMSA, and a fragment taken from the *gntR* promoter was used as the negative control.

(c) The deficient expression *pcaQ* in Δ*pcaQ* was confirmed by RT-qPCR, compared with the wild-type strain (WT). *p* value is 1.120E-05.

(d) Validation of two putative binding sites of MetR by EMSA and a fragment taken from the *gntR* promoter was used as the negative control.

(e) The deficient expression *metR* in Δ*metR* was confirmed by RT-qPCR, compared with the wild-type strain (WT). *p* value is 7.935E-08.

(f) The deficient expression PSPPH_3297 in the PSPPH_3297 mutant strain (ΔPSPPH_3297) was confirmed by RT-qPCR, compared with the wild-type strain (WT). *p* value is 3.710E-04.

Three independent biological replicates were performed. Statistic *p* values by two-tailed Student's t-test are shown; *** $p < 0.001$ (c-f). Error bars show standard deviations. Source data to this figure are provided in the Source Data file.

Supplementary Figure 3

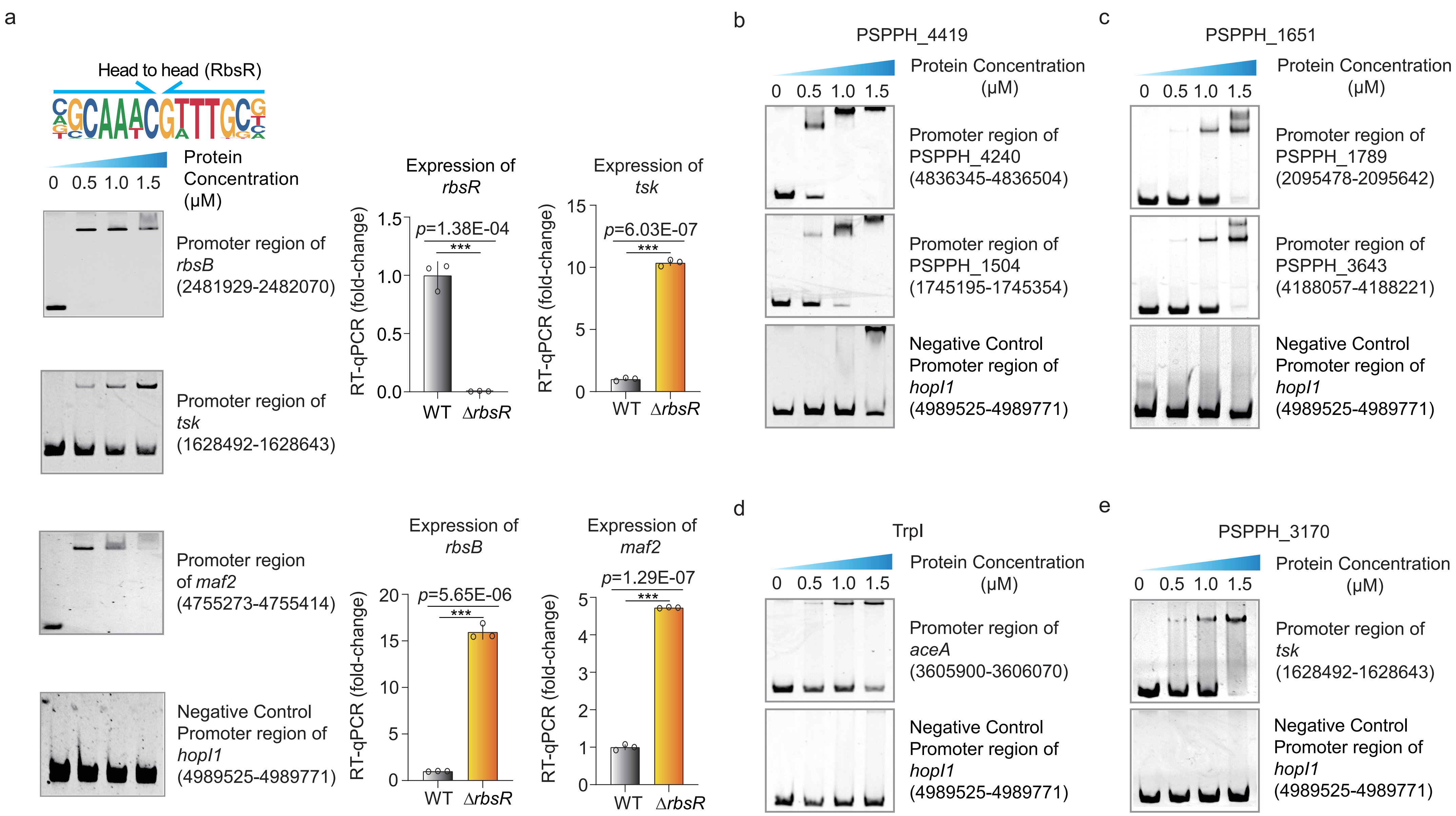

Supplementary Figure 3. Validation of putative TF binding sites, related to Fig. 2.

(a) Validation of the binding of RbsR by EMSA and RT-qPCR. The putative binding sites from the promoters of the target genes *rbsB*, *tsk* and *maf2* were verified using EMSA, respectively, and a fragment taken from the *hopl1* promoter was used as the negative control. The expression of target gene *rbsR*, *tsk*, *rbsB* and *maf2* were measured in wild-type and *rbsR* mutant (Δ*rbsR*) by RT-qPCR, respectively. Logo shows binding site model of RbsR. *p* value is 5.651E-06 (*rbsB*), 6.033E-07 (*tsk*), and 1.290E-07 (*maf2*), respectively.

(b) Validation of two putative binding sites of PSPPH_4419 by EMSA. The putative binding sites from the promoters of PSPPH_4240 and PSPPH_1504 were verified using EMSA, respectively.

(c) Validating the binding site of PSPPH_1651 by EMSA. The putative binding sites from the promoter of target gene PSPPH_1789 and PSPPH_3643 were verified using EMSA, respectively.

(d) Validating the binding site of Trpl by EMSA. The putative binding sites from the promoter of target gene *aceA* was verified using EMSA.

(e) Validating the binding site of PSPPH_3170 by EMSA. The putative binding sites from the promoter of target gene *tsk* was verified using EMSA.

Three independent biological replicates were performed. Statistic p values by two-tailed Student's t-test are shown; *** $p < 0.001$. Error bars show standard deviations. A fragment taken from the *hopl1* promoter was used as the negative control (B-E). Source data to this figure are provided in the Source Data file.

Supplementary Figure 4

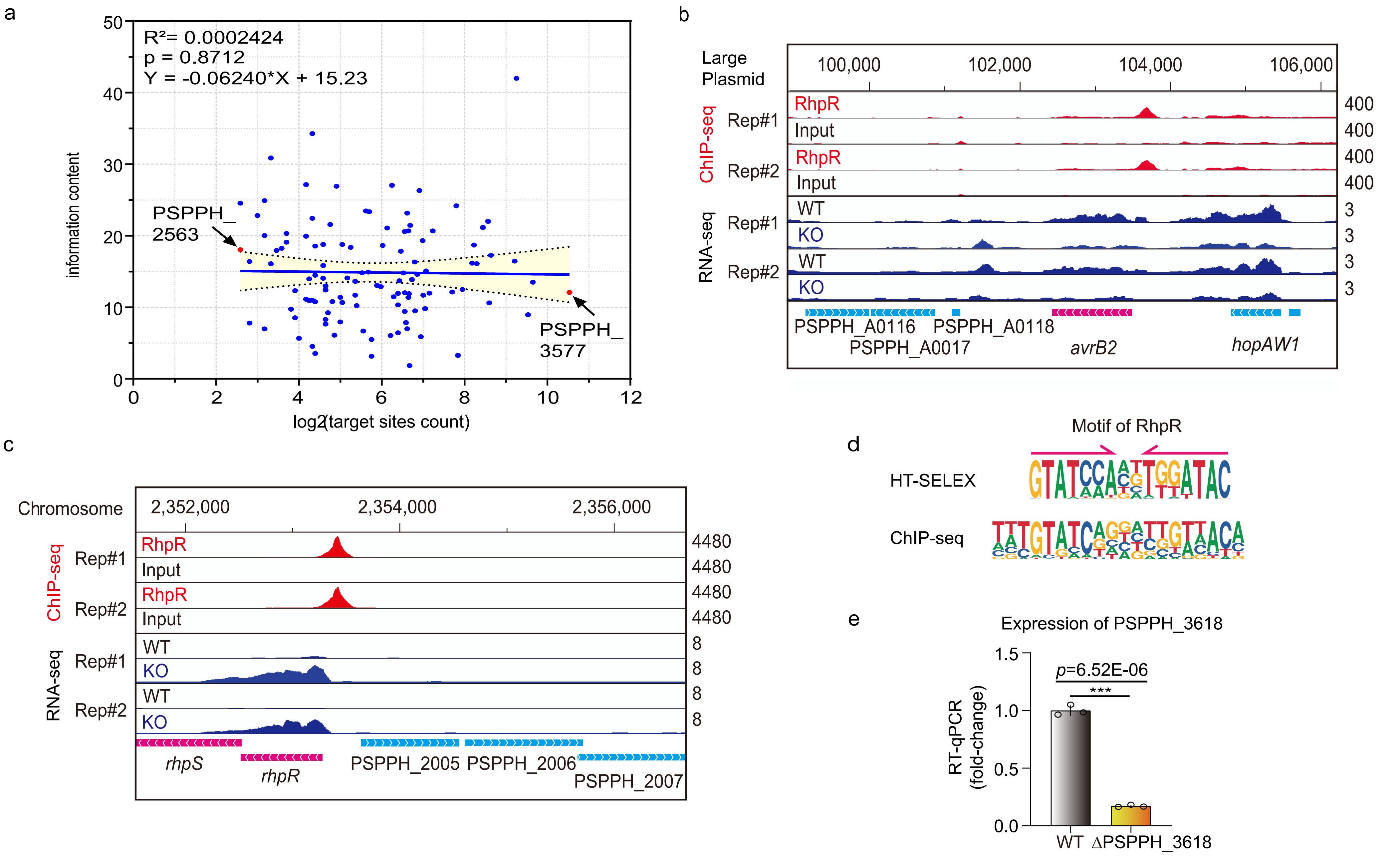

Supplementary Figure 4. Transcriptional regulation in T3SS pathway.

(a) Scatterplot shows the trend of the number of putative genomic sites of a TF by PWM model (x-axis) along the information content of the model (y-axis). Note that there's no significant correlation between the two variables (p=0.8712, two-sided), suggesting that the differential number of targets of different TFs may not result from the motif complexity or degeneracy but more likely depends on the genomic composition. Red dotes highlight the two TFs with the most of least number of putative genomic sites shown in Fig. 3a.

(b) Binding of RhpR to a putative site in the promoter of the *avrB2* gene on the large plasmid is proven by ChIP-seq in the wild-type cells. The RNA-seq result shows that loss of *rhpS* leads to increased RhpR expression. The increased binding of RhpR caused reduced expression of *avrB*. Rep# indicates the independent biological replicate.

(c) Binding of RhpR to a putative site in its own promoter is proven by ChIP-seq in the wild-type cells. The RNA-seq result shows that loss of *rhpS* leads to increased RhpR expression in knockout cells (KO) compared to the wild-type cells (WT). Red peaks show RhpR validated binding sites in the promoter of *rhpR* gene, blue peaks show the RhpR expression. Rep# indicates the biological replicates.

(d) Comparison of the binding motif of RhpR obtained from HT-SELEX (upper) or ChIP-seq (lower). Arrows indicate half-sites in dimeric sites.

(e) The deficient expression of PSPPH_3618 in ΔPSPPH_3618 was confirmed by RT-qPCR, compared with the wild-type strain (WT). p value is 6.521E-06.

Three independent replicates were performed. Statistic p values by two-tailed Student's t-test are shown; *** $p < 0.001$. Error bars show standard deviations. Source data to this figure are provided in the Source Data file.

Supplementary Table 1a: Family classification for TF based on DBD domain, related to Fig.1b, Fig.2, Supplementary Fig. 2 and 3.

DBD family catelogies for 301 TFs we used in the study			
**No.**	**TF Locus_tag**	**TF Name**	**DBD Family (Pfam)**
1	PSPPH_3155		LysR family
2	PSPPH_2921		
3	PSPPH_3817		
4	PSPPH_2301		
5	PSPPH_3022		
6	PSPPH_4062	IciA	
7	PSPPH_3079		
8	PSPPH_3464		
9	PSPPH_3911	MetR	
10	PSPPH_0141		
11	PSPPH_3233		
12	PSPPH_3990		
13	PSPPH_3460		
14	PSPPH_1100		
15	PSPPH_2439		
16	PSPPH_2095	PcaQ	
17	PSPPH_0037	TrpI	
18	PSPPH_2555		
19	PSPPH_3611		
20	PSPPH_0711		
21	PSPPH_2309	MexT	
22	PSPPH_2469		
23	PSPPH_4620		
24	PSPPH_2772		
25	PSPPH_2638		
26	PSPPH_2048	CysB	
27	PSPPH_2693		
28	PSPPH_1493		
29	PSPPH_4998		
30	PSPPH_1259		
31	PSPPH_3798		
32	PSPPH_2444		
33	PSPPH_0190	OxyR	
34	PSPPH_4638		
35	PSPPH_0700		
36	PSPPH_1435		
37	PSPPH_2193		
38	PSPPH_5144		
39	PSPPH_3411		
40	PSPPH_3278		
41	PSPPH_2688		
42	PSPPH_1737		
43	PSPPH_1951		
44	PSPPH_1758		
45	PSPPH_0442		
46	PSPPH_3014		
47	PSPPH_2580		
48	PSPPH_3618		
49	PSPPH_2631		
50	PSPPH_0077		
51	PSPPH_2476		
52	PSPPH_2236		
53	PSPPH_2967		
54	PSPPH_0589		
55	PSPPH_1579		
56	PSPPH_2454		

57	PSPPH_2553		
58	PSPPH_3241		
59	PSPPH_5132		
60	PSPPH_2689		
61	PSPPH_0411		
62	PSPPH_2300		
63	PSPPH_3468		
64	PSPPH_2491		
65	PSPPH_3643		
66	PSPPH_0031		
67	PSPPH_2423		
68	PSPPH_2295		
69	PSPPH_2204		
70	PSPPH_0755		
71	PSPPH_1049		
72	PSPPH_2106		
73	PSPPH_4622		
74	PSPPH_2407		
75	PSPPH_1730		
76	PSPPH_1728		
77	PSPPH_3487		
78	PSPPH_3244	AefR	
79	PSPPH_2448		
80	PSPPH_3066		
81	PSPPH_3654		
82	PSPPH_2305		
83	PSPPH_2214		TetR family
84	PSPPH_4495		
85	PSPPH_3202	PsrA	
86	PSPPH_2733		
87	PSPPH_3479		
88	PSPPH_4788		
89	PSPPH_0376		
90	PSPPH_0735		
91	PSPPH_3268		
92	PSPPH_4899		
93	PSPPH_1651		
94	PSPPH_3872		
95	PSPPH_3983		
96	PSPPH_4012		
97	PSPPH_4768	BetI	
98	PSPPH_0218		
99	PSPPH_1969		
100	PSPPH_2983		
101	PSPPH_2315		
102	PSPPH_5138		
103	PSPPH_3936		
104	PSPPH_3577		
105	PSPPH_0349	HutC	
106	PSPPH_0286		
107	PSPPH_3031		
108	PSPPH_1771		
109	PSPPH_3004		
110	PSPPH_0486		GntR family
111	PSPPH_2055		
112	PSPPH_1776		
113	PSPPH_3005		
114	PSPPH_3471		
115	PSPPH_5087		

116	PSPPH_2937	PhnF	
117	PSPPH_2473		
118	PSPPH_2720		
119	PSPPH_1595		
120	PSPPH_4253		
121	PSPPH_1233		
122	PSPPH_2563		
123	PSPPH_3048		
124	PSPPH_3285		
125	PSPPH_2357		
126	PSPPH_4744		
127	PSPPH_4127		
128	PSPPH_2421		
129	PSPPH_4841		
130	PSPPH_4692		
131	PSPPH_2862		
132	PSPPH_3431	PobR	
133	PSPPH_2893		
134	PSPPH_1097		AraC/XylS family
135	PSPPH_3122		
136	PSPPH_3486		
137	PSPPH_2597		
138	PSPPH_1962		
139	PSPPH_2994		
140	PSPPH_1670		
141	PSPPH_2984		
142	PSPPH_3522		
143	PSPPH_2774		
144	PSPPH_1615		
145	PSPPH_0082		
146	PSPPH_2337		
147	PSPPH_1169		
148	PSPPH_1680		
149	PSPPH_1270	HrpR	
150	PSPPH_2426		
151	PSPPH_2229		
152	PSPPH_1271	HrpS	
153	PSPPH_4853	NtrC	
154	PSPPH_0252	AlgB	
155	PSPPH_0857	TsiR	Fis family
156	PSPPH_3385	FleR	
157	PSPPH_3907		
158	PSPPH_4002		
159	PSPPH_4255		
160	PSPPH_0736	PilR	
161	PSPPH_3387	FleQ	
162	PSPPH_3168		
163	PSPPH_4448		
164	PSPPH_0146		
165	PSPPH_1460	CpxR	
166	PSPPH_2004	RhpR	
167	PSPPH_2022	KdpE	
168	PSPPH_3127		
169	PSPPH_2376	BaeS1	
170	PSPPH_3547		
171	PSPPH_3040		
172	PSPPH_1195		
173	PSPPH_3453		
174	PSPPH_4828		OmpR family

175	PSPPH_0246	OmpR	OmpR family
176	PSPPH_3962	TctD	
177	PSPPH_3294		
178	PSPPH_2995		
179	PSPPH_1179	GltR	
180	PSPPH_5114	PhoB	
181	PSPPH_3730	PhoP	
182	PSPPH_4075	ColR	
183	PSPPH_3737	RstA	
184	PSPPH_2539		
185	PSPPH_2917		
186	PSPPH_3170		
187	PSPPH_1393		
188	PSPPH_0653		
189	PSPPH_2151		
190	PSPPH_2478		
191	PSPPH_0226		
192	PSPPH_5057		
193	PSPPH_4324		Cro/CI family
194	PSPPH_1584		
195	PSPPH_2792		
196	PSPPH_4694		
197	PSPPH_4674		
198	PSPPH_4673		
199	PSPPH_2194		
200	PSPPH_2327		
201	PSPPH_2800		
202	PSPPH_4238		
203	PSPPH_1818		
204	PSPPH_5171		
205	PSPPH_3800		
206	PSPPH_4241		
207	PSPPH_0642		
208	PSPPH_3220		
209	PSPPH_1364		LuxR family
210	PSPPH_1374		
211	PSPPH_2328	GacA	
212	PSPPH_0778		
213	PSPPH_1906		
214	PSPPH_2015		
215	PSPPH_2149		
216	PSPPH_4419		
217	PSPPH_4336		
218	PSPPH_1762		
219	PSPPH_0239		
220	PSPPH_5042		
221	PSPPH_4377		
222	PSPPH_1244		AsnC family
223	PSPPH_4356		
224	PSPPH_4700		
225	PSPPH_5054		
226	PSPPH_0222	Irp	
227	PSPPH_2338		
228	PSPPH_3257	GntR	
229	PSPPH_2128	RbsR	
230	PSPPH_0846	FruR	LacI family
231	PSPPH_5193		
232	PSPPH_4730		
233	PSPPH_2905		
234	PSPPH_4858		

235	PSPPH_1617		MarR family
236	PSPPH_1519		
237	PSPPH_2432		
238	PSPPH_1800		
239	PSPPH_3555	MarR	
240	PSPPH_3297		
241	PSPPH_3645	MgrA	
242	PSPPH_1160		Cold Shock Domain family
243	PSPPH_3099	CspD	
244	PSPPH_1380	CapB	
245	PSPPH_2133	CapA	
246	PSPPH_3779		
247	PSPPH_4644		MerR family
248	PSPPH_2142		
249	PSPPH_1011		
250	PSPPH_2630		
251	PSPPH_4870	CadR	
252	PSPPH_3247	IclR	IclR family
253	PSPPH_4020	PcaR	
254	PSPPH_1960		
255	PSPPH_2665		
256	PSPPH_2832		
257	PSPPH_3900	GlpR	DeoR family
258	PSPPH_5204		
259	PSPPH_4844		
260	PSPPH_2188		
261	PSPPH_3439	Srl	
262	PSPPH_1734		ArsR family
263	PSPPH_1222		
264	PSPPH_4056		
265	PSPPH_4637		
266	PSPPH_4822		
267	PSPPH_0406		RpiR family
268	PSPPH_5145		
269	PSPPH_1187	HexR	
270	PSPPH_3195		
271	PSPPH_4612		BirA family
272	PSPPH_2219		
273	PSPPH_1456		
274	PSPPH_1136		HxlR family
275	PSPPH_2859		
276	PSPPH_5194		
277	PSPPH_2872		Sigma factor 54
278	PSPPH_3530		
279	PSPPH_3182	DksA	Zinc finger family
280	PSPPH_0861	DksA2	
281	PSPPH_0263		Fur family
282	PSPPH_4209	Fur	
283	PSPPH_0101		ParB family
284	PSPPH_0102		
285	PSPPH_3203	LexA2	LexA repressor
286	PSPPH_2677	LexA1	
287	PSPPH_3351	Anr	CRP family
288	PSPPH_0676	Vfr	
289	PSPPH_3923		PadR-like family
290	PSPPH_3027		PucR C-terminal
291	PSPPH_3504		Arc-like family
292	PSPPH_1308	IscR	Rrf2 family
293	PSPPH_1580		RmlC family
294	PSPPH_4920		Not annotated

295	PSPPH_5139		PAI 2-type
296	PSPPH_4320		Ribonuclease H-like family
297	PSPPH_1700	Lon	Lon family
298	PSPPH_0863	SfsA	SfsA family
299	PSPPH_0061	AlgQ	Rsd/AlgQ family
300	PSPPH_0069	AlgR	LytTr family
301	PSPPH_4646		AraC family

**The successful fractions of 301 TFs for HT-SELEX**			
**Family Name**	**Number of TFs**	**Number of successful TFs**	**Successful Fraction**
LysR family	68	25	0.367647059
TetR family	29	7	0.24137931
GntR family	26	13	0.5
AraC/XylS family	21	1	0.047619048
Fis family	20	7	0.35
OmpR family	20	10	0.5
Cro/CI family	17	3	0.176470588
LuxR family	16	7	0.4375
AsnC family	9	1	0.111111111
LacI family	8	6	0.75
MarR family	7	4	0.571428571
Cold Shock Domain family	5	2	0.4
MerR family	5	2	0.4
IclR family	5	2	0.4
DeoR family	5	1	0.2
ArsR family	5	0	0
RpiR family	4	2	0.5
BirA family	3	1	0.333333333
HxlR family	3	0	0
Sigma factor 54	2	0	0
Zinc finger family	2	0	0
Fur family	2	2	1
ParB family	2	0	0
LexA repressor	2	1	0.5
CRP family	2	1	0.5
PadR-like family	1	0	0
PucR C-terminal	1	0	0
Arc-like family	1	0	0
Rrf2 family	1	1	1
RmlC family	1	1	1
Not annotated	1	0	0
PAI 2-type	1	0	0
Ribonuclease H-like family	1	0	0
Lon family	1	0	0
SfsA family	1	0	0
Rsd/AlgQ family	1	0	0
LytTr family	1	0	0
AraC family	1	0	0

Primers	Sequences
**EMSA**	
EMSA-PSPPH_2621-F	TACTTTAAAGAACGGGATGAT
EMSA-PSPPH_2621-F	GGTGCGGCATATCCCGT
EMSA-PSPPH_3297-F	CTGAGTCGTGAACAAT
EMSA-PSPPH_3297-F	TCTACGCCCCCTCAAA
EMSA-PSPPH_0985-F	CTTTTAGGCCTTTGTTAGCT
EMSA-PSPPH_0985-R	GTACCTGCCAGAATGTTGA
EMSA-*katB*-F	ACAATTAGTTGAATAATTTCCTT
EMSA-*katB*-R	TAGACGGCGTGATTAAACG
EMSA-*rbsB*-F	AACAGATTTCATTTTCGACGG
EMSA-*rbsB*-R	ATGAGTTCATCTCCGATCTT
EMSA-*tsk*-F	GGGCGTTGCGGATCGAG
EMSA-*tsk*-R	CCCGATCGGTCATCTTCT
EMSA-*maf2*-F	AAGCGAGGGCATAAGGACA
EMSA-*maf2*-R	CTGTTGTGGCCCTGGATC
EMSA-*nuoA*-F	CTGACGCCCGGCTG
EMSA-*nuoA*-R	GTGCTTGATAAAGAGTC
EMSA-*sdhC*-F	CACCACCCAGGAAGT
EMSA-*sdhC*-R	TTTGTCCCGGCAACG
EMSA-PSPPH_4240-F	GGCCGCTACCGTGTCAG
EMSA-PSPPH_4240-R	TTCTGTTAAGCATCAGGCG
EMSA-PSPPH_1504-F	CTGCTCGGGCCGCGC
EMSA-PSPPH_1504-R	TCGCGAAACCGAGCGCT
EMSA-PSPPH_1024-F	TCAAGAATTACAGTTGTGCC
EMSA-PSPPH_1024-R	TTCACAGAAATCCCCTGAAA
EMSA-PSPPH_4247-F	TGCATACAAATGAGCAGGC
EMSA-PSPPH_4247-R	GATGGGTGTTCGGTGATG
EMSA-PSPPH_1789-F	AGGTTCTGTCAGCCG
EMSA-PSPPH_1789-R	CGCATGACGCGCTGT
EMSA-PSPPH_3643-F	AAAGCGTTTTTCAGC
EMSA-PSPPH_3643-R	CATCAATCTCCCAGATG
EMSA-*aceA*-F	GGAGCGAGGCAATCATGC
EMSA-*aceA*-R	TTTTGCGCGTAAAGGTCG
EMSA-NC(3776311-3776381)-*gntR*-F	CATAGGGGTTGCCAAATGT
EMSA-NC(3776311-3776381)-*gntR*-R	ATAAAGCATTGTCACCTTCC
EMSA-NC(4989525-4989771)-*hopI*1-F	CTGCTCCAGCAATGAGC
EMSA-NC(4989525-4989771)-*hopI*1-R	ATGCTCGCGTCAGTGTTC
**RT-qPCR**	
RT-PSPPH_2621-F	CAGACCATTACCCCGAGTGT
RT-PSPPH_2621-R	GCCAGAAACAGGTGCTCAAA
RT-PSPPH_0985-F	GGGTGGAATCTTCTGGTGGA
RT-PSPPH_0985-R	CGTCTGTTTCTCTTTGCGCT

RT-*katB*-F	ACCAAGTTCTACACGGCAGA
RT-*katB*-R	ACCATGTCCGGGAACTTGAT
RT-*maf2*-F	CTTGATGGGCGCATTCTTGG
RT-*maf2*-R	ACAATACGGGTTTCACTGCG
RT-*tsk*-F	CCAAGCCCAACGATGACATT
RT-*tsk*-R	CATGATCCCAGACGCCTTTG
RT-*rbsB*-F	GAAGTCGCTCGCCAATGAAT
RT-*rbsB*-R	AGGTGTCTGACTCGTCCTTG
RT-*nuoA*-F	CGCAGCAAGAACGAACCTTT
RT-*nuoA*-R	AGCATCGCGACCAGATAGAA
RT-*sdhC*-F	GTGAAGTCAAGGCGTGTCTG
RT-*sdhC*-R	CGGAGTCCATGATGAGGTGA
**Lux reporter**	
pMS402-PSPPH_2621-F	TCGTCTTCACCTCGAGGGGATCCTGTTTCAGAGCAGTGAGAAA
pMS402-PSPPH_2621-R	GCGGCCGCAACTAGAGGATCCGCACAGCAGATAAAAAGCAA
pMS402-PSPPH_3297-F	TCGTCTTCACCTCGAGGGGATCCGGAGTAGCTCGCAAGGTA
pMS402-PSPPH_3297-R	GCGGCCGCAACTAGAGGATCCGAATCACCGGGGGCTGA
**Mutant validation**	
PSPPH_3297-verify-PCR-F	GTAGCAATTATTTTTTGAAACAAT
PSPPH_3297-verify-PCR-R	AGGGTGCCGGCGGCC
PSPPH_3297-qPCR-F	CATCAGGTAAAAGTCGCGCA
PSPPH_3297-qPCR-R	GATCATTGGGGTCTTCGCTG
metR-verify-PCR-F	CACCCTGATCCTGTTTCCA
metR-verify-PCR-R	TCCGCTGACACGCGACC
metR-qPCR-F	CATTGTTCCGGAAGACCTGC
metR-qPCR-R	CATCACCGTCAATTCCGAGG
rbsR-verify-PCR-F	TCGGTGGCACCAGCCTG
rbsR-verify-PCR-R	CCACACTGCCACCCAGG
rbsR-qPCR-F	ATCGAGGACTACTGTGAGCG
rbsR-qPCR-R	AGCAAAACACGCACGTAACT
pcaQ-varify-PCR-F	GTTGCCGAAGCGGTTTTAC
pcaQ-varify-PCR-R	GGAAATGGATTGCGGGATA
pcaQ-qPCR-F	CCATCTGGTGTGCTTTCTCG
pcaQ-qPCR-R	GCAGTGTTTCCAGCTCCTTG
**Mutant construction**	
pK18-PSPPH_3297-up-F	AAACAGCTATGACATGATTACGAATTCGCACGCCCTGCGCAATG
pK18-PSPPH_3297-up-R (BamHI)	TTGGATCCGAATCACCGGGGGCTGAG
pK18-PSPPH_3297-down-F(BamHI)	TTGGATCCTATTTCAGGCCTTCGCCGA
pK18-PSPPH_3297-down-R	AACGACGGCCAGTGCCAAGCTTTTCATGAAGTCCGCGTCGT
pK18-*metR*-up-F	AAACAGCTATGACATGATTACGAATTCGCCACACTTGCGTGACAG
pK18-*metR*-up-R (BamHI)	TTGGATCCACAGCGCCGACTGCGTC
pK18-*metR*-down-F(BamHI)	TTGGATCCTCGCGCCGACATGCTCG
pK18-*metR*-down-R	AACGACGGCCAGTGCCAAGCTTCCGCTGGTTGCGCTTCG

pK18-*pcaQ*-up-F	AAACAGCTATGACATGATTACGAATTCTTCTACGTCGTGTACTCCG
pK18-*pcaQ*-up-R (BamHI)	TTGGATCCATGCGCGTCGGGTCCG
pK18-*pcaQ*-down-F(BamHI)	TTGGATCCCCATTTGGTTATGGATAACC
pK18-*pcaQ*-down-R	AACGACGGCCAGTGCCAAGCTTGCCGGGAACGCACGCA
pK18-*rbsR*-up-F	AAACAGCTATGACATGATTACGAATTCACGACTCATGAAAAATACTCC
pK18-*rbsR*-up-R (BamHI)	TTGGATCCATCAGCCCTGGCGCCGT
pK18-*rbsR*-down-F(BamHI)	TTGGATCCTCGCCGCACCGCATACA
pK18-*rbsR*-down-R	AACGACGGCCAGTGCCAAGCTTTGTCTGGAAAACTGCATCAA
**TF cloning**	
pET28a-PSPPH_4238-F	AGCAAATGGGTCGCGGATCCATGCATATCAGGTTGTCG
pET28a-PSPPH_4238-R	CGGAGCTCGAATTCGGATCCTTATTGGTCGAGTAAACGG
pET28a-PSPPH_1818-F	AGCAAATGGGTCGCGGATCCATGTGCAGTGCCATTAAAG
pET28a-PSPPH_1818-R	CGGAGCTCGAATTCGGATCCTTACCTGATCAAACCCCG
pET28a-PSPPH_1615-F	AGCAAATGGGTCGCGGATCCATGAAGATAAACGGGGCC
pET28a-PSPPH_1615-R	CGGAGCTCGAATTCGGATCCTTAGACCATGCCAATGTTG
pET28a-PSPPH_3285-F	AGCAAATGGGTCGCGGATCCATGGAAAACATGAAAGGCAC
pET28a-PSPPH_3285-R	CGGAGCTCGAATTCGGATCCTTAAAGCCCTGAAAAAAGCCCTG
pET28a-PSPPH_3530-F	AGCAAATGGGTCGCGGATCCATGCGCATCAAGGTGCA
pET28a-PSPPH_3530-R	CGGAGCTCGAATTCGGATCCTTAATCAGCGGATTGTTGA
pET28a-PSPPH_0082-F	AGCAAATGGGTCGCGGATCCATGACCCTCATCGCCACT
pET28a-PSPPH_0082-R	CGGAGCTCGAATTCGGATCCTTAGCGCGGAATGC
pET28a-PSPPH_2337-F	AGCAAATGGGTCGCGGATCCATGGCACTCAACAGCG
pET28a-PSPPH_2337-R	CGGAGCTCGAATTCGGATCCTTAGAGCCCTCGAAAATC
pET28a-PSPPH_1169-F	AGCAAATGGGTCGCGGATCCATGCGCATCCATGTCAG
pET28a-PSPPH_1169-R	CGGAGCTCGAATTCGGATCCTTAGCGCTTGCCCGG
pET28a-PSPPH_2872-F	AGCAAATGGGTCGCGGATCCATGACCCACCCCCTGT
pET28a-PSPPH_2872-R	CGGAGCTCGAATTCGGATCCTTAGCGAATACCCAGGC
pET28a-PSPPH_2357-F	AGCAAATGGGTCGCGGATCCATGTCCTGGGATCTGTTTC
pET28a-PSPPH_2357-R	CGGAGCTCGAATTCGGATCCTTAGGCACCTGGACATT
pET28a-PSPPH_0406-F	AGCAAATGGGTCGCGGATCCATGGACATTCTGTACCAGAT
pET28a-PSPPH_0406-R	CGGAGCTCGAATTCGGATCCTTAGTCTCCCAACGGC
pET28a-PSPPH_5145-F	AGCAAATGGGTCGCGGATCCATGAATCTGCTGCAACAC
pET28a-PSPPH_5145-R	CGGAGCTCGAATTCGGATCCTTAGTCTTCGTGAGTCTTTA
pET28a-*hexR*-F	AGCAAATGGGTCGCGGATCCATGGACCGCGTAAGAAAC
pET28a-*hexR*-R	CGGAGCTCGAATTCGGATCCTTAGCCTTGATCCTCGATC
pET28a-PSPPH_3195-F	AGCAAATGGGTCGCGGATCCATGACCCGCGCCGAT
pET28a-PSPPH_3195-R	CGGAGCTCGAATTCGGATCCTTAATCATCAAACCCGACCT
pET28a-PSPPH_1680-F	AGCAAATGGGTCGCGGATCCATGTCTCAATCCAGCGC
pET28a-PSPPH_1680-R	CGGAGCTCGAATTCGGATCCTTAAACGATCCGCTGTTC
pET28a-PSPPH_2426-F	AGCAAATGGGTCGCGGATCCATGCCGGGCCGAGGT
pET28a-PSPPH_2426-R	CGGAGCTCGAATTCGGATCCTTAGGCGCCCCCTGG
pET28a-PSPPH_3168-F	AGCAAATGGGTCGCGGATCCATGTCAGGGCAACTGCT
pET28a-PSPPH_3168-R	CGGAGCTCGAATTCGGATCCTTACACCGACAAATGCA
pET28a-PSPPH_2229-F	AGCAAATGGGTCGCGGATCCATGCATGGATCGGCCC
pET28a-PSPPH_2229-R	CGGAGCTCGAATTCGGATCCTTAGCGAATCTGATGCTTG

pET28a-PSPPH_3522-F	AGCAAATGGGTCGCGGATCCATGTTCCAGATCGAGCCAT
pET28a-PSPPH_3522-R	CGGAGCTCGAATTCGGATCCTTAACCGCGTTCGGC
pET28a-PSPPH_4744-F	AGCAAATGGGTCGCGGATCCATGACATCGTTCAATCAAGGG
pET28a-PSPPH_4744-R	CGGAGCTCGAATTCGGATCCTTAAAGCCTCACACTCGC
pET28a-PSPPH_4127-F	AGCAAATGGGTCGCGGATCCATGCCGATCCAACACG
pET28a-PSPPH_4127-R	CGGAGCTCGAATTCGGATCCTTACAGCTTTTCCTTCTGG
pET28a-*ftrA*-F	AGCAAATGGGTCGCGGATCCATGAAAAATCATCTCGTTGTCG
pET28a-*ftrA*-R	CGGAGCTCGAATTCGGATCCTTACGCCCGGGCAA
pET28a-*ntrC*-F	AGCAAATGGGTCGCGGATCCATGAGCCGTAGTGAAACTG
pET28a-*ntrC*-R	CGGAGCTCGAATTCGGATCCTTAGCCCTCATCAGCCTC
pET28a-*fleQ*-F	AGCAAATGGGTCGCGGATCCATGTGGCGTGAAATCAAG
pET28a-*fleQ*-R	CGGAGCTCGAATTCGGATCCTCAATCATCTGCCTGTTCAT
pET28a-*algB*-F	AGCAAATGGGTCGCGGATCCATGGAAGCAGCCACTGAG
pET28a-*algB*-R	CGGAGCTCGAATTCGGATCCTTACAGGTTGTACTGTTTACG
pET28a-PSPPH_0146-F	AGCAAATGGGTCGCGGATCCATGAGTTCGGACACAGC
pET28a-PSPPH_0146-R	CGGAGCTCGAATTCGGATCCTTAATCGTTGTCATCGGA
pET28a-*fleR*-F	AGCAAATGGGTCGCGGATCCATGTCGATCAACGTGCTG
pET28a-*fleR*-R	CGGAGCTCGAATTCGGATCCTTAGGTAGCGAACAGATAG
pET28a-PSPPH_3907-F	AGCAAATGGGTCGCGGATCCATGAGCATCAACAGCGAC
pET28a-PSPPH_3907-R	CGGAGCTCGAATTCGGATCCTTAAAGCCCGTATTTCTTGA
pET28a-PSPPH_4002-F	AGCAAATGGGTCGCGGATCCATGAACGCGGTCATCGT
pET28a-PSPPH_4002-R	CGGAGCTCGAATTCGGATCCTTAGCTCAGGAACATTTCCCG
pET28a-PSPPH_5171-F	AGCAAATGGGTCGCGGATCCATGAACACAGCCGCCA
pET28a-PSPPH_5171-R	CGGAGCTCGAATTCGGATCCTTAACGGAGTTCTTTGAGC
pET28a-PSPPH_3800-F	AGCAAATGGGTCGCGGATCCATGACCTACGACATCCTG
pET28a-PSPPH_3800-R	CGGAGCTCGAATTCGGATCCTTAACTGGACGAGATGGA
pET28a-PSPPH_4241-F	AGCAAATGGGTCGCGGATCCATGCCGTGCAGAATCATTG
pET28a-PSPPH_4241-R	CGGAGCTCGAATTCGGATCCTCAGTGCTGTTTGCCGGC
pET28a-PSPPH_0642-F	AGCAAATGGGTCGCGGATCCATGATTCGTGTTTTGGTA
pET28a-PSPPH_0642-R	CGGAGCTCGAATTCGGATCCTCAGAGCAGTTTGTGCTC
pET28a-PSPPH_3220-F	AGCAAATGGGTCGCGGATCCATGACCTGCAATCTGTTA
pET28a-PSPPH_3220-R	CGGAGCTCGAATTCGGATCCTCAATCGTCCAGACTGAT
pET28a-PSPPH_1364-F	AGCAAATGGGTCGCGGATCCATGCGCGTCATCGTGGCT
pET28a-PSPPH_1364-R	CGGAGCTCGAATTCGGATCCTCAGGACATGCCGCTTTC
pET28a-PSPPH_1374-F	AGCAAATGGGTCGCGGATCCATGAATAAAGTGCTGATC
pET28a-PSPPH_1374-R	CGGAGCTCGAATTCGGATCCTCAGACCAGCCCGTTACG
pET28a-*gacA*-F	AGCAAATGGGTCGCGGATCCATGATTAAGGTGCTAGTT
pET28a-*gacA*-R	CGGAGCTCGAATTCGGATCCTCAGGCGCTGGCATCAAC
pET28a-*cpxR*-F	AGCAAATGGGTCGCGGATCCATGACTTCCGTATTGCTTG
pET28a-*cpxR*-R	CGGAGCTCGAATTCGGATCCTCATGGCGTAATCAACAG
pET28a-PSPPH_0778-F	AGCAAATGGGTCGCGGATCCATGCAGCTCAGAATCATC
pET28a-PSPPH_0778-R	CGGAGCTCGAATTCGGATCCCTACGTACTAAGGTGGGT
pET28a-PSPPH_1906-F	AGCAAATGGGTCGCGGATCCATGACTGACACTGTGCGA
pET28a-PSPPH_1906-R	CGGAGCTCGAATTCGGATCCTCATCGCCACTCGCTCGT
pET28a-PSPPH_4419-F	AGCAAATGGGTCGCGGATCCATGGATAACTACCCGGTC
pET28a-PSPPH_4419-R	CGGAGCTCGAATTCGGATCCTCAGTCCTGGCTTTCGATC

pET28a-*kdpE*-F	AGCAAATGGGTCGCGGATCCATGAGCCAGGCGGCAA
pET28a-*kdpE*-R	CGGAGCTCGAATTCGGATCCTTAGTGGTCGGCCATCAG
pET28a-*cvsR*-F	AGCAAATGGGTCGCGGATCCATGCGAATTCTTCTGGTTG
pET28a-*cvsR*-R	CGGAGCTCGAATTCGGATCCTTAAACTCCATCCCGGCC
pET28a-*colR*-F	AGCAAATGGGTCGCGGATCCATGACCTCCATGACCACCG
pET28a-*colR*-R	CGGAGCTCGAATTCGGATCCTCAAAGCTCGAGCTTGTA
pET28a-*baeS1*-F	AGCAAATGGGTCGCGGATCCATGAATGTCCTGATCGTTGAT
pET28a-*baeS1*-R	CGGAGCTCGAATTCGGATCCTTAGAGCTGCTGCATCATCTT
pET28a-*algR*-F	AGCAAATGGGTCGCGGATCCATGAGCGAGCTGTTACTAATTG
pET28a-*algR*-R	CGGAGCTCGAATTCGGATCCTTACAAAGCATAATAATAGCCGCG
pET28a-PSPPH_3547-F	AGCAAATGGGTCGCGGATCCATGACCCGAATTCTGGCAA
pET28a-PSPPH_3547-R	CGGAGCTCGAATTCGGATCCTTAGACGGGTTCGGCAAT
pET28a-PSPPH_3040-F	AGCAAATGGGTCGCGGATCCATGCGCGTGCTGATTATCG
pET28a-PSPPH_3040-R	CGGAGCTCGAATTCGGATCCTTAACCCGCACTGCGG
pET28a-PSPPH_1195-F	AGCAAATGGGTCGCGGATCCATGCGCCTGCTTCTTGTCG
pET28a-PSPPH_1195-R	CGGAGCTCGAATTCGGATCCTTATGCCTTGAGCTCCAGC
pET28a-PSPPH_3453-F	AGCAAATGGGTCGCGGATCCATGCGAATCCTTGTCGTAGA
pET28a-PSPPH_3453-R	CGGAGCTCGAATTCGGATCCTTATTCACGCACTTCCAGC
pET28a-PSPPH_4828-F	AGCAAATGGGTCGCGGATCCATGAGCAGCACCGCACAA
pET28a-PSPPH_4828-R	CGGAGCTCGAATTCGGATCCTTAGCGATTACCAGCACCAT
pET28a-*ompR*-F	AGCAAATGGGTCGCGGATCCATGCGTGTTCTTCTTGTGGA
pET28a-*ompR*-R	CGGAGCTCGAATTCGGATCCTTAATCATCGCGGCTTTCAA
pET28a-*tctD*-F	AGCAAATGGGTCGCGGATCCATGCATATCCTGCTGATTGAAGAC
pET28a-*tctD*-R	CGGAGCTCGAATTCGGATCCTTAGGGAAGGCTGGCGT
pET28a-PSPPH_3294-F	AGCAAATGGGTCGCGGATCCATGAACATTCTGGTTATTGAAGATCAC
pET28a-PSPPH_3294-R	CGGAGCTCGAATTCGGATCCTTAGCGCTGGACACACAA
pET28a-PSPPH_2995-F	AGCAAATGGGTCGCGGATCCATGAGTTCAGTCAGTAAATCGATC
pET28a-PSPPH_2995-R	CGGAGCTCGAATTCGGATCCTTAATAGCCGTTGCCCGC
pET28a-*gltR*-F	AGCAAATGGGTCGCGGATCCATGGCTGGCAGGAGCAT
pET28a-*gltR*-R	CGGAGCTCGAATTCGGATCCTTAGCTCTTTGTCGAGAAGC
pET28a-*phoB*-F	AGCAAATGGGTCGCGGATCCATGAAGTTGTTGGTAGTTGAGGA
pET28a-*phoB*-R	CGGAGCTCGAATTCGGATCCTTACGTACAGCGCTCATTG
pET28a-*phoP*-F	AGCAAATGGGTCGCGGATCCATGGAGCCACACACCTG
pET28a-*phoP*-R	CGGAGCTCGAATTCGGATCCTTAGCCGCTGAACACGT
pET28a-*rstA*-F	AGCAAATGGGTCGCGGATCCATGAGTGACGAAGACATCCA
pET28a-*rstA*-R	CGGAGCTCGAATTCGGATCCTTAACGCCGCACAGGG
pET28a-PSPPH_4255-F	AGCAAATGGGTCGCGGATCCATGCCTGACAGCCGAG
pET28a-PSPPH_4255-R	CGGAGCTCGAATTCGGATCCTTAGACGGATGCAGCG
pET28a-PSPPH_4841-F	AGCAAATGGGTCGCGGATCCATGCCTTCCACACACTG
pET28a-PSPPH_4841-R	CGGAGCTCGAATTCGGATCCTTAGTCTGGAAGGTTTGA
pET28a-PSPPH_4692-F	AGCAAATGGGTCGCGGATCCATGAGCACCATCCACTCC
pET28a-PSPPH_4692-R	CGGAGCTCGAATTCGGATCCTTACAACCGATACTGCCC
pET28a-PSPPH_2862-F	AGCAAATGGGTCGCGGATCCATGGGCGAATCTGCCC
pET28a-PSPPH_2862-R	CGGAGCTCGAATTCGGATCCTTAGCAGCCCTTCGAAG
pET28a-PSPPH_1580-F	AGCAAATGGGTCGCGGATCCATGTCCGAGCCAGTAAAGT
pET28a-PSPPH_1580-R	CGGAGCTCGAATTCGGATCCTTAGCTTTGGATTGCGC

pET28a-anr-F	AGCAAATGGGTCGCGGATCCATGGTCGCAATAACTTTAACGC
pET28a-*anr*-R	CGGAGCTCGAATTCGGATCCTTAGCGCGTACCGTACAC
pET28a-*vfr*-F	AGCAAATGGGTCGCGGATCCATGCCGACTAACGGACAA
pET28a-vfr-R	CGGAGCTCGAATTCGGATCCTTACTTGAACAAATCACCGGGC
pET28a-PSPPH_4646-F	AGCAAATGGGTCGCGGATCCATGGCCAGACCTGCCAC
pET28a-PSPPH_4646-R	CGGAGCTCGAATTCGGATCCTTATTTCTGCTGACGAAACGCTT
pET28a-*pobR*-F	AGCAAATGGGTCGCGGATCCATGAAGCTTGAGCAATACCTGG
pET28a-*pobR*-R	CGGAGCTCGAATTCGGATCCTTAATCGCCCTGCGCA
pET28a-PSPPH_2917-F	AGCAAATGGGTCGCGGATCCATGCACAAAGAAAATTCCCCGC
pET28a-PSPPH_2917-R	CGGAGCTCGAATTCGGATCCTTAGAGCACGACGTTACG
pET28a-PSPPH_3170-F	AGCAAATGGGTCGCGGATCCATGGCTGATCGTCAGAGC
pET28a-PSPPH_3170-R	CGGAGCTCGAATTCGGATCCTTAGGCTTCGGCTTGAACT
pET28a-PSPPH_1160-F	AGCAAATGGGTCGCGGATCCATGGCGATGCTTAACGGAAA
pET28a-PSPPH_1160-R	CGGAGCTCGAATTCGGATCCTTAAGCGTGTGCATGTTCAG
pET28a-*cspD*-F	AGCAAATGGGTCGCGGATCCATGTCCAATCGCCAAACTGG
pET28a-*cspD*-R	CGGAGCTCGAATTCGGATCCTTAGATAACTTGAACTTCTTCAGCTTG
pET28a-*capB*-F	AGCAAATGGGTCGCGGATCCATGTCTAATCGCCAAACCGG
pET28a-*capB*-R	CGGAGCTCGAATTCGGATCCTTAGTCAACGCGGACTTGTT
pET28a-*capA*-F	AGCAAATGGGTCGCGGATCCATGGGAAATCGTGATACTGGC
pET28a-*capA*-R	CGGAGCTCGAATTCGGATCCTTAGCGGCGGGGC
pET28a-PSPPH_3779-F	AGCAAATGGGTCGCGGATCCATGAGCCCTATGAAACAGGC
pET28a-PSPPH_3779-R	CGGAGCTCGAATTCGGATCCTTAGGACTCTTCGGAAGCGA
pET28a-PSPPH_3504-F	AGCAAATGGGTCGCGGATCCATGAATAACAATAAGCGCCCCA
pET28a-PSPPH_3504-R	CGGAGCTCGAATTCGGATCCTTAGGCGTCGGTATTCTCC
pET28a-PSPPH_2338-F	AGCAAATGGGTCGCGGATCCATGATCCGCATTGGTTCCCG
pET28a-PSPPH_2338-R	CGGAGCTCGAATTCGGATCCTTAGGAGCTTTCCCGTACCA
pET28a-*gntR*-F	AGCAAATGGGTCGCGGATCCATGGCGACTATCAAGGATGTC
pET28a-*gntR*-R	CGGAGCTCGAATTCGGATCCTTATTCATTGGACAAAGTGTTCG
pET28a-*rbsR*-F	AGCAAATGGGTCGCGGATCCATGAAACTGAGCGATATCGC
pET28a-*rbsR*-R	CGGAGCTCGAATTCGGATCCTTACACCTCGTGAATACGTT
pET28a-*fruR*-F	AGCAAATGGGTCGCGGATCCATGACCTCAGTTAAAGACGTTG
pET28a-*fruR*-R	CGGAGCTCGAATTCGGATCCTTATAACGATTCCCTCACCAC
pET28a-PSPPH_5193-F	AGCAAATGGGTCGCGGATCCATGGTAACGCAGACCGGT
pET28a-PSPPH_5193-R	CGGAGCTCGAATTCGGATCCTTACTGAGGCTGAGCATGAA
pET28a-PSPPH_4730-F	AGCAAATGGGTCGCGGATCCATGGCCGACGAGAAACCC
pET28a-PSPPH_4730-R	CGGAGCTCGAATTCGGATCCTTATGTACTCTCGCGCTCTA
pET28a-PSPPH_4858-F	AGCAAATGGGTCGCGGATCCATGAATACATCAGGCGATCGA
pET28a-PSPPH_4858-R	CGGAGCTCGAATTCGGATCCTTACAACAGCAGCATCGC
pET28a-PSPPH_4673-F	AGCAAATGGGTCGCGGATCCATGGGCGTGCCCG
pET28a-PSPPH_4673-R	CGGAGCTCGAATTCGGATCCTTATAGCCTGGCGGTCATT
pET28a-PSPPH_1393-F	AGCAAATGGGTCGCGGATCCATGAACAGAAAATGGTATGAAGTC
pET28a-PSPPH_1393-R	CGGAGCTCGAATTCGGATCCTTAGAGACGCATCGTCAT
pET28a-PSPPH_0653-F	AGCAAATGGGTCGCGGATCCATGACGAAGAAACGCATCCT
pET28a-PSPPH_0653-R	CGGAGCTCGAATTCGGATCCTTAAACTAGGTTAGCATTCCAGAC
pET28a-PSPPH_2151-F	AGCAAATGGGTCGCGGATCCATGGACAATCCATTCCAACTG
pET28a-PSPPH_2151-R	CGGAGCTCGAATTCGGATCCTTAAAAATTCGCCGGTGTG

pET28a-PSPPH_2478-F	AGCAAATGGGTCGCGGATCCATGGACGTGGGTGAACGA
pET28a-PSPPH_2478-R	CGGAGCTCGAATTCGGATCCTTAGAAATTGGCTGGCGTT
pET28a-PSPPH_0226-F	AGCAAATGGGTCGCGGATCCATGAGCCAAGCCGCGT
pET28a-PSPPH_0226-R	CGGAGCTCGAATTCGGATCCTTACCGGCCCAGTGTC
pET28a-PSPPH_5057-F	AGCAAATGGGTCGCGGATCCATGGACAAGTTGGCGAAAG
pET28a-PSPPH_5057-R	CGGAGCTCGAATTCGGATCCTTAGGCGGGTTGCAGC
pET28a-PSPPH_4324-F	AGCAAATGGGTCGCGGATCCATGGATCTTCAAGTGATATCCCG
pET28a-PSPPH_4324-R	CGGAGCTCGAATTCGGATCCTTACGACTCGCCTCGC
pET28a-PSPPH_1584-F	AGCAAATGGGTCGCGGATCCATGGACTATTCCCTCATCACAAC
pET28a-PSPPH_1584-R	CGGAGCTCGAATTCGGATCCTTAGTCGAATACTCCCTCAAT
pET28a-PSPPH_4694-F	AGCAAATGGGTCGCGGATCCATGAGCATCGGCGAAAATCT
pET28a-PSPPH_4694-R	CGGAGCTCGAATTCGGATCCTTACGCCACATCGTTTTCCT
pET28a-PSPPH_2792-F	AGCAAATGGGTCGCGGATCCATGAGTGGAATCGGTTGTCG
pET28a-PSPPH_2792-R	CGGAGCTCGAATTCGGATCCTTAAGGAAGCTTTTCAGGAGCG
pET28a-PSPPH_2327-F	AGCAAATGGGTCGCGGATCCATGTGTGGCAACAAGCCT
pET28a-PSPPH_2327-R	CGGAGCTCGAATTCGGATCCTTACTTCAACTTCGAGGTCA
pET28a-PSPPH_2194-F	AGCAAATGGGTCGCGGATCCATGAAAACCGTCCATAACGC
pET28a-PSPPH_2194-R	CGGAGCTCGAATTCGGATCCTTACGAGCTAAGTAAGCGC
pET28a-PSPPH_4674-F	AGCAAATGGGTCGCGGATCCATGGAATTGAAGGAAGCATTTGC
pET28a-PSPPH_4674-R	CGGAGCTCGAATTCGGATCCTTACACTTCCAGCGTATCGC
pET28a-PSPPH_2800-F	AGCAAATGGGTCGCGGATCCATGAACATCGGTCAGGCGG
pET28a-PSPPH_2800-R	CGGAGCTCGAATTCGGATCCTTAGCAACCCACTCCCGA
pET28a-PSPPH_4644-F	AGCAAATGGGTCGCGGATCCATGCTGGAACCAAGTCATAAC
pET28a-PSPPH_4644-R	CGGAGCTCGAATTCGGATCCTTACGCTTTGAGCATAACCAG
pET28a-PSPPH_2142-F	AGCAAATGGGTCGCGGATCCATGAATGAAACAGACACAGAGGT
pET28a-PSPPH_2142-R	CGGAGCTCGAATTCGGATCCTTAGATAAGTCCGAGCTTGCC
pET28a-PSPPH_1011-F	AGCAAATGGGTCGCGGATCCATGAGCAGTCAGACTTACAGC
pET28a-PSPPH_1011-R	CGGAGCTCGAATTCGGATCCTTAGTGTGAAGTGGCAGC
pET28a-PSPPH_2630-F	AGCAAATGGGTCGCGGATCCATGAAAATCGGCGAACTGG
pET28a-PSPPH_2630-R	CGGAGCTCGAATTCGGATCCTTAATGCCCATGACTCCTC
pET28a-*cadR*-F	AGCAAATGGGTCGCGGATCCATGGTCGATATCAGAACACGTG
pET28a-*cadR*-R	CGGAGCTCGAATTCGGATCCTTAATGCACCGCCGG
pET28a-PSPPH_2539-F	AGCAAATGGGTCGCGGATCCATGCTTCGCGACAACG
pET28a-PSPPH_2539-R	CGGAGCTCGAATTCGGATCCTTACTCCACCAAAATAACCC
pET28a-PSPPH_2015-F	AGCAAATGGGTCGCGGATCCATGGATACCACCATCGTATGC
pET28a-PSPPH_2015-R	CGGAGCTCGAATTCGGATCCTTAGCAAATCGCCGCC
pET28a-PSPPH_2149-F	AGCAAATGGGTCGCGGATCCATGACTCAGGACATCGAAAGC
pET28a-PSPPH_2149-R	CGGAGCTCGAATTCGGATCCTTAGTTTGACGGACGGCT
pET28a-*iclR*-F	AGCAAATGGGTCGCGGATCCATGAACGATGAACTGCGTA
pET28a-*iclR*-R	CGGAGCTCGAATTCGGATCCTTAGGTAAACAGCTGCGT
pET28a-*pcaR*-F	AGCAAATGGGTCGCGGATCCATGCACAACGATTCGATCAT
pET28a-*pcaR*-R	CGGAGCTCGAATTCGGATCCTTAGCCGCCGAGATCG
pET28a-PSPPH_1960-F	AGCAAATGGGTCGCGGATCCATGACTGAAGACACAATCAAGC
pET28a-PSPPH_1960-R	CGGAGCTCGAATTCGGATCCTTATTCGTTGATTCGACGAGC
pET28a-PSPPH_2665-F	AGCAAATGGGTCGCGGATCCATGAATAATTCTACTGATCGGAATA
pET28a-PSPPH_2665-R	CGGAGCTCGAATTCGGATCCTTAATCCGCCTGCCCG

pET28a-PSPPH_2832-F	AGCAAATGGGTCGCGGATCCATGCTGACCTTGTTAAAGCTT
pET28a-PSPPH_2832-R	CGGAGCTCGAATTCGGATCCTTAAGAATCATCTCGCAACC
pET28a-PSPPH_4612-F	AGCAAATGGGTCGCGGATCCATGGAAGGCTTGGTCAAAC
pET28a-PSPPH_4612-R	CGGAGCTCGAATTCGGATCCTTAATGGCGCAACAGTTTG
pET28a-PSPPH_5042-F	AGCAAATGGGTCGCGGATCCATGAGCAAGCTCGACAGA
pET28a-PSPPH_5042-R	CGGAGCTCGAATTCGGATCCTTAGTTGCGCAAATGGGT
pET28a-PSPPH_4377-F	AGCAAATGGGTCGCGGATCCATGCAGCCTAAATTGAGCC
pET28a-PSPPH_4377-R	CGGAGCTCGAATTCGGATCCTTAGTCCAGCGGCAACTC
pET28a-PSPPH_2219-F	AGCAAATGGGTCGCGGATCCATGACCTCGCCGACGTT
pET28a-PSPPH_2219-R	CGGAGCTCGAATTCGGATCCTTAGGCCGAAGGTTTGAC
pET28a-PSPPH_1244-F	AGCAAATGGGTCGCGGATCCATGAAATTCATCATGAAAGCAATA
pET28a-PSPPH_1244-R	CGGAGCTCGAATTCGGATCCTTAAATGGGTAATTGCGTGGT
pET28a-PSPPH_1762-F	AGCAAATGGGTCGCGGATCCATGGACAAATTCGACCGG
pET28a-PSPPH_1762-R	CGGAGCTCGAATTCGGATCCTTAGCCGCCAAACGAT
pET28a-PSPPH_5054-F	AGCAAATGGGTCGCGGATCCATGCCCGATACCCGC
pET28a-PSPPH_5054-R	CGGAGCTCGAATTCGGATCCTTATACCGGCTGGCCG
pET28a-PSPPH_4700-F	AGCAAATGGGTCGCGGATCCATGACGCTGGATAAATACGAT
pET28a-PSPPH_4700-R	CGGAGCTCGAATTCGGATCCTTAGTATTCCTGATTGCCGTT
pET28a-PSPPH_4356-F	AGCAAATGGGTCGCGGATCCATGCGCACTCAACACCA
pET28a-PSPPH_4356-R	CGGAGCTCGAATTCGGATCCTTAGTCGGCAATCGGCA
pET28a-*lrp*-F	AGCAAATGGGTCGCGGATCCATGCAAACCGAGCTGGA
pET28a-*lrp*-R	CGGAGCTCGAATTCGGATCCTTAGCCACCCTGGCG
pET28a-PSPPH_0239-F	AGCAAATGGGTCGCGGATCCATGAACGTCAAGCAGCTTC
pET28a-PSPPH_0239-R	CGGAGCTCGAATTCGGATCCTTATCGACTATTGCGAGCATC
pET28a-PSPPH_3155-F	AGCAAATGGGTCGCGGATCCATGCAACTCTACGGCGTAC
pET28a-PSPPH_3155-R	CGGAGCTCGAATTCGGATCCTTAGGTCTTTGCCAGCTC
pET28a-PSPPH_2921-F	AGCAAATGGGTCGCGGATCCATGCACATCAATCTCTGGGA
pET28a-PSPPH_2921-R	CGGAGCTCGAATTCGGATCCTTACTGGCGGTACTCATCG
pET28a-PSPPH_3817-F	AGCAAATGGGTCGCGGATCCATGAAGTTCAGTCTGCGAC
pET28a-PSPPH_3817-R	CGGAGCTCGAATTCGGATCCTTAGACAAAAGGTACTCTTTC
pET28a-PSPPH_2301-F	AGCAAATGGGTCGCGGATCCATGGACCTGCGCGATCT
pET28a-PSPPH_2301-R	CGGAGCTCGAATTCGGATCCTTACCCCCAACCCAGAA
pET28a-PSPPH_3022-F	AGCAAATGGGTCGCGGATCCATGTTCGACTACAAATTGTTATCTG
pET28a-PSPPH_3022-R	CGGAGCTCGAATTCGGATCCTTAGTCTTCGCTCAGCG
pET28a-*iciA*-F	AGCAAATGGGTCGCGGATCCATGCCCATCAATTTCGACCT
pET28a-*iciA*-R	CGGAGCTCGAATTCGGATCCTTAGTTCCCCGCTGAAC
pET28a-PSPPH_3079-F	AGCAAATGGGTCGCGGATCCATGACTGAACGTATACCGCC
pET28a-PSPPH_3079-R	CGGAGCTCGAATTCGGATCCTTAAGGTGCCGCGA
pET28a-PSPPH_3464-F	AGCAAATGGGTCGCGGATCCATGCTTGAAATACGCCAC
pET28a-PSPPH_3464-R	CGGAGCTCGAATTCGGATCCTTAGCGAACCACGCTAA
pET28a-*metR*-F	AGCAAATGGGTCGCGGATCCATGGACATCAAGCAACTTAAG
pET28a-*metR*-R	CGGAGCTCGAATTCGGATCCTTAGCGCGGCATTTCT
pET28a-PSPPH_0141-F	AGCAAATGGGTCGCGGATCCATGAGACTCAACCTGCG
pET28a-PSPPH_0141-R	CGGAGCTCGAATTCGGATCCTTACGGCGCTTTGCTG
pET28a-PSPPH_3233-F	AGCAAATGGGTCGCGGATCCATGGCAAGCACAGCTGAC
pET28a-PSPPH_3233-R	CGGAGCTCGAATTCGGATCCTTACTCCGGCGTCGCTA

pET28a-PSPPH_3990-F	AGCAAATGGGTCGCGGATCCATGAGCAGAATCTTCAACG
pET28a-PSPPH_3990-R	CGGAGCTCGAATTCGGATCCTTAGATCACTGCTTCGTTCA
pET28a-PSPPH_3460-F	AGCAAATGGGTCGCGGATCCATGGACACCCTGCAAAAC
pET28a-PSPPH_3460-R	CGGAGCTCGAATTCGGATCCTTAGAACGAGGACTTGAG
pET28a-PSPPH_1100-F	AGCAAATGGGTCGCGGATCCATGGACCGTTTTGACGC
pET28a-PSPPH_1100-R	CGGAGCTCGAATTCGGATCCTTACGATTGCTCGGCTC
pET28a-PSPPH_2439-F	AGCAAATGGGTCGCGGATCCATGAATATTGATACCCGCATCA
pET28a-PSPPH_2439-R	CGGAGCTCGAATTCGGATCCTTATGCATACTGCGCTTTCC
pET28a-*pcaQ*-F	AGCAAATGGGTCGCGGATCCATGAGCCGCGACCTTC
pET28a-*pcaQ*-R	CGGAGCTCGAATTCGGATCCTTAGTTTTTGATCGGTTCC
pET28a-*trpI*-F	AGCAAATGGGTCGCGGATCCATGAATTATCCAAACCTTGATG
pET28a-*trpI*-R	CGGAGCTCGAATTCGGATCCTTAGCGGTTTGCCGG
pET28a-PSPPH_2555-F	AGCAAATGGGTCGCGGATCCATGAAAATTGATGATATCGATGCGT
pET28a-PSPPH_2555-R	CGGAGCTCGAATTCGGATCCTTAGGCGGCTGCTGAA
pET28a-PSPPH_3611-F	AGCAAATGGGTCGCGGATCCATGAAAGCCAGATCTGATGA
pET28a-PSPPH_3611-R	CGGAGCTCGAATTCGGATCCTTACCTGCGCACAACG
pET28a-PSPPH_0711-F	AGCAAATGGGTCGCGGATCCATGAATCGTAACGACCTGCG
pET28a-PSPPH_0711-R	CGGAGCTCGAATTCGGATCCTTATAAACTATCCGGATCC
pET28a-*mexT*-F	AGCAAATGGGTCGCGGATCCATGTTCGAGCTTTCTCAACT
pET28a-*mexT*-R	CGGAGCTCGAATTCGGATCCTTAATTTTCATCCACCCTGA
pET28a-PSPPH_2469-F	AGCAAATGGGTCGCGGATCCATGAGCGTCCGTCGC
pET28a-PSPPH_2469-R	CGGAGCTCGAATTCGGATCCTTATCGGGTAGCGGC
pET28a-PSPPH_4620-F	AGCAAATGGGTCGCGGATCCATGCGCCATCTCGATAC
pET28a-PSPPH_4620-R	CGGAGCTCGAATTCGGATCCTTACAAGGCTGCCATCG
pET28a-PSPPH_2772-F	AGCAAATGGGTCGCGGATCCATGGATCGATTCAACGCC
pET28a-PSPPH_2772-R	CGGAGCTCGAATTCGGATCCTTAGGAAACCAGTCTGTC
pET28a-PSPPH_2638-F	AGCAAATGGGTCGCGGATCCATGAAGCTCCAACAACTGC
pET28a-PSPPH_2638-R	CGGAGCTCGAATTCGGATCCTTAGTGCACCGGCAGT
pET28a-*cysB*-F	AGCAAATGGGTCGCGGATCCATGCTTATGGATTTGCGTC
pET28a-*cysB*-R	CGGAGCTCGAATTCGGATCCTTACTCGGCATAAATGCTC
pET28a-PSPPH_2693-F	AGCAAATGGGTCGCGGATCCATGCTGTCCACTGACC
pET28a-PSPPH_2693-R	CGGAGCTCGAATTCGGATCCTTAATCCACCCGCAACG
pET28a-PSPPH_1493-F	AGCAAATGGGTCGCGGATCCATGAGACCGGTGAATTTCGA
pET28a-PSPPH_1493-R	CGGAGCTCGAATTCGGATCCTTAGGCGATCTCGCTT
pET28a-PSPPH_4998-F	AGCAAATGGGTCGCGGATCCATGCAGCCTGATGACGAT
pET28a-PSPPH_4998-R	CGGAGCTCGAATTCGGATCCTTAAAACTCAAACCCCTGC
pET28a-PSPPH_1259-F	AGCAAATGGGTCGCGGATCCATGCGATTTACTCTCCGC
pET28a-PSPPH_1259-R	CGGAGCTCGAATTCGGATCCTTAATACAGGCTTGGCAG
pET28a-PSPPH_3798-F	AGCAAATGGGTCGCGGATCCATGTCCCTGACGTTGC
pET28a-PSPPH_3798-R	CGGAGCTCGAATTCGGATCCTTAACGCCGCGCC
pET28a-PSPPH_2444-F	AGCAAATGGGTCGCGGATCCATGACTCTTACAGAATTACGCTA
pET28a-PSPPH_2444-R	CGGAGCTCGAATTCGGATCCTTAACTTGCGACTGTTTTCG
pET28a-*oxyR*-F	AGCAAATGGGTCGCGGATCCATGTTGCGCTTTGACGAC
pET28a-*oxyR*-R	CGGAGCTCGAATTCGGATCCTTAAAACAACCCAACGGGTG
pET28a-PSPPH_4638-F	AGCAAATGGGTCGCGGATCCATGATCAGCATGGAAATGTTG
pET28a-PSPPH_4638-R	CGGAGCTCGAATTCGGATCCTTACCAGCATCCGTGT

pET28a-PSPPH_0700-F	AGCAAATGGGTCGCGGATCCATGAAAGCGCCCCGC
pET28a-PSPPH_0700-R	CGGAGCTCGAATTCGGATCCTTAGGCGTGTTGTGGA
pET28a-PSPPH_1435-F	AGCAAATGGGTCGCGGATCCATGGCGCTTGACCTCA
pET28a-PSPPH_1435-R	CGGAGCTCGAATTCGGATCCTTATTGCGCCGCAAA
pET28a-PSPPH_2193-F	AGCAAATGGGTCGCGGATCCATGCGTATGACATTGCGT
pET28a-PSPPH_2193-R	CGGAGCTCGAATTCGGATCCTTAAGTTGCCCGCGG
pET28a-PSPPH_5144-F	AGCAAATGGGTCGCGGATCCATGCTGTTACGCCATATACG
pET28a-PSPPH_5144-R	CGGAGCTCGAATTCGGATCCTTAGATATCACTCACCAGTTTG
pET28a-PSPPH_3411-F	AGCAAATGGGTCGCGGATCCATGCCTGAGCGCCC
pET28a-PSPPH_3411-R	CGGAGCTCGAATTCGGATCCTTACTCTCCGGCAAACG
pET28a-PSPPH_3278-F	AGCAAATGGGTCGCGGATCCATGCGTATCGAGCCCC
pET28a-PSPPH_3278-R	CGGAGCTCGAATTCGGATCCTTAGGGCCGGTGTGT
pET28a-PSPPH_2688-F	AGCAAATGGGTCGCGGATCCATGGACAGGCTCAAGGC
pET28a-PSPPH_2688-R	CGGAGCTCGAATTCGGATCCTTATCGGTCTTGAAGAGAGG
pET28a-PSPPH_1737-F	AGCAAATGGGTCGCGGATCCATGGATCTCGCCAACCTT
pET28a-PSPPH_1737-R	CGGAGCTCGAATTCGGATCCTTATGTCTGCGCATCGAG
pET28a-PSPPH_1951-F	AGCAAATGGGTCGCGGATCCATGAGCGCAATTCTCGAT
pET28a-PSPPH_1951-R	CGGAGCTCGAATTCGGATCCTTAAAGCCGAAAATACTTCTG
pET28a-PSPPH_1758-F	AGCAAATGGGTCGCGGATCCATGCAGATCGATGACGAA
pET28a-PSPPH_1758-R	CGGAGCTCGAATTCGGATCCTTAGGTCGCCTGCTC
pET28a-PSPPH_0442-F	AGCAAATGGGTCGCGGATCCATGAATCGAAATGAACTGCG
pET28a-PSPPH_0442-R	CGGAGCTCGAATTCGGATCCTTAGCCTGCCAGGG
pET28a-PSPPH_3014-F	AGCAAATGGGTCGCGGATCCATGCTCAATTACCGTCAGC
pET28a-PSPPH_3014-R	CGGAGCTCGAATTCGGATCCTTAACTTGCGTCGGTAAAC
pET28a-PSPPH_2580-F	AGCAAATGGGTCGCGGATCCATGAACCCTTTTGAAGACATG
pET28a-PSPPH_2580-R	CGGAGCTCGAATTCGGATCCTTAGCCAGGCGGTTC
pET28a-PSPPH_3618-F	AGCAAATGGGTCGCGGATCCATGAACCTGAGCAAGGTC
pET28a-PSPPH_3618-R	CGGAGCTCGAATTCGGATCCTTATGCTTTCTCGAGGCC
pET28a-PSPPH_2631-F	AGCAAATGGGTCGCGGATCCATGCCTCGCAGTCTCC
pET28a-PSPPH_2631-R	CGGAGCTCGAATTCGGATCCTTAATGCAGTACTTCAACTAACGG
pET28a-PSPPH_0077-F	AGCAAATGGGTCGCGGATCCATGCAATATGAAATCACCCATG
pET28a-PSPPH_0077-R	CGGAGCTCGAATTCGGATCCTTAGGCGGATCTGGAC
pET28a-PSPPH_2476-F	AGCAAATGGGTCGCGGATCCATGTTTGATCTCAATGATCTGTT
pET28a-PSPPH_2476-R	CGGAGCTCGAATTCGGATCCTTAGTGGTCTTGAATGCG
pET28a-PSPPH_2236-F	AGCAAATGGGTCGCGGATCCATGGATCGGATCATGGCG
pET28a-PSPPH_2236-R	CGGAGCTCGAATTCGGATCCTTAAGGCTCTGCGCC
pET28a-PSPPH_2967-F	AGCAAATGGGTCGCGGATCCATGCTGAACAAACGTCATC
pET28a-PSPPH_2967-R	CGGAGCTCGAATTCGGATCCTTAACGGGCCGGTTGAT
pET28a-PSPPH_0589-F	AGCAAATGGGTCGCGGATCCATGAACCAGACACAGGACC
pET28a-PSPPH_0589-R	CGGAGCTCGAATTCGGATCCTTAATGCGGGTCTTGCA
pET28a-PSPPH_1579-F	AGCAAATGGGTCGCGGATCCATGAACCTTCGCCGCT
pET28a-PSPPH_1579-R	CGGAGCTCGAATTCGGATCCTTACTCTGTACGGCGATTG
pET28a-PSPPH_2454-F	AGCAAATGGGTCGCGGATCCATGGATCTGGTTCAGCTGG
pET28a-PSPPH_2454-R	CGGAGCTCGAATTCGGATCCTTATCCGGCTTGCGG
pET28a-PSPPH_2553-F	AGCAAATGGGTCGCGGATCCATGCTCAACAGTAATGCCCT
pET28a-PSPPH_2553-R	CGGAGCTCGAATTCGGATCCTTAGAGCAGACCGCG

pET28a-PSPPH_3241-F	AGCAAATGGGTCGCGGATCCATGAATCTGGAAAGCAAATGGC
pET28a-PSPPH_3241-R	CGGAGCTCGAATTCGGATCCTTATGCTTCGCCCGGC
pET28a-PSPPH_5132-F	AGCAAATGGGTCGCGGATCCATGAACACCAGGAGACTGA
pET28a-PSPPH_5132-R	CGGAGCTCGAATTCGGATCCTTACAAAGGGTCGGGAAAC
pET28a-PSPPH_2689-F	AGCAAATGGGTCGCGGATCCATGGAATTGCGCCATCTG
pET28a-PSPPH_2689-R	CGGAGCTCGAATTCGGATCCTTATCGTGCGACATTGCG
pET28a-PSPPH_0411-F	AGCAAATGGGTCGCGGATCCATGCCCATGATCAAAGAACT
pET28a-PSPPH_0411-R	CGGAGCTCGAATTCGGATCCTTAACGAGTCAGAGCGTGT
pET28a-PSPPH_2300-F	AGCAAATGGGTCGCGGATCCATGAGCCTGGTGCAAGAT
pET28a-PSPPH_2300-R	CGGAGCTCGAATTCGGATCCTTAATTGCGAAACGCCG
pET28a-PSPPH_3468-F	AGCAAATGGGTCGCGGATCCATGGATTTGTTTCAGGCGAT
pET28a-PSPPH_3468-R	CGGAGCTCGAATTCGGATCCTTACCGCCCCCAGAG
pET28a-PSPPH_2491-F	AGCAAATGGGTCGCGGATCCATGGCCAACTATCCAGGC
pET28a-PSPPH_2491-R	CGGAGCTCGAATTCGGATCCTTAAGGTTTGAAGCCGTC
pET28a-PSPPH_3643-F	AGCAAATGGGTCGCGGATCCATGTTTGAAGCTTTTGGCG
pET28a-PSPPH_3643-R	CGGAGCTCGAATTCGGATCCTTACACCGCAATCGACG
pET28a-PSPPH_0031-F	AGCAAATGGGTCGCGGATCCATGCAAATATCTGATATAGAGGTG
pET28a-PSPPH_0031-R	CGGAGCTCGAATTCGGATCCTTACAAGGTGACTCCCGC
pET28a-PSPPH_2295-F	AGCAAATGGGTCGCGGATCCATGACGCTTTACGTCAAC
pET28a-PSPPH_2295-R	CGGAGCTCGAATTCGGATCCTTAGTCAGTTTGCGCATT
pET28a-PSPPH_0218-F	AGCAAATGGGTCGCGGATCCATGACCAATCTTTTGCTCTATC
pET28a-PSPPH_0218-R	CGGAGCTCGAATTCGGATCCTTAGTACACATGTACCGC
pET28a-PSPPH_1969-F	AGCAAATGGGTCGCGGATCCATGAAACGCTACGAAAAATTCG
pET28a-PSPPH_1969-R	CGGAGCTCGAATTCGGATCCTTAGAACGACGCGATGAT
pET28a-PSPPH_2983-F	AGCAAATGGGTCGCGGATCCATGAAAAACAACATGGATTTCGCT
pET28a-PSPPH_2983-R	CGGAGCTCGAATTCGGATCCTTAGGGCAGACGCGC
pET28a-PSPPH_2315-F	AGCAAATGGGTCGCGGATCCATGCCCATCGCCCCT
pET28a-PSPPH_2315-R	CGGAGCTCGAATTCGGATCCTTACTCGGACCAGGCCT
pET28a-PSPPH_5138-F	AGCAAATGGGTCGCGGATCCATGAACCTGCGTGTACCT
pET28a-PSPPH_5138-R	CGGAGCTCGAATTCGGATCCTTACCGATGGTGAGTGTC
pET28a-PSPPH_4822-F	AGCAAATGGGTCGCGGATCCATGTACTCCATGAGCACA
pET28a-PSPPH_4822-R	CGGAGCTCGAATTCGGATCCTCATTCGCGCCGGACCAG
pET28a-*lexA1*-F	AGCAAATGGGTCGCGGATCCATGATCAAACTGACGCCA
pET28a-*lexA1*-R	CGGAGCTCGAATTCGGATCCTTAGCGGCGAATGACGCC
pET28a-*lexA2*-F	AGCAAATGGGTCGCGGATCCATGAAACCGATGTCAGAAG
pET28a-*lexA2*-R	CGGAGCTCGAATTCGGATCCTCAACTCAACCGCGTCAG
pET28a-PSPPH_3936-F	AGCAAATGGGTCGCGGATCCATGAACCTGCCTCCCCGC
pET28a-PSPPH_3936-R	CGGAGCTCGAATTCGGATCCTCACACTACTTCCAGACG
pET28a-*glpR*-F	AGCAAATGGGTCGCGGATCCATGTCGAAGCGCAATACA
pET28a-*glpR*-R	CGGAGCTCGAATTCGGATCCCTAGCTGACAGCAGCACA
pET28a-PSPPH_5204-F	AGCAAATGGGTCGCGGATCCATGATCCCCGATCAGCGC
pET28a-PSPPH_5204-R	CGGAGCTCGAATTCGGATCCCTAGCGCTTGCCACCCAC
pET28a-PSPPH_4844-F	AGCAAATGGGTCGCGGATCCATGTCCCTCTTGCTTGCG
pET28a-PSPPH_4844-R	CGGAGCTCGAATTCGGATCCTCAGGGATAGAGGGTGTC
pET28a-PSPPH_2188-F	AGCAAATGGGTCGCGGATCCATGAAAGTCGCCAACCGT
pET28a-PSPPH_2188-R	CGGAGCTCGAATTCGGATCCTCAGCCGGGCAGGGCAAT

pET28a-*srl*-F	AGCAAATGGGTCGCGGATCCATGCGATGGTATGATGCC
pET28a-*srl*-R	CGGAGCTCGAATTCGGATCCTCAACCGCCACCCAGCAC
pET28a-PSPPH_3577-F	AGCAAATGGGTCGCGGATCCATGTCGACTCCGCCCGCC
pET28a-PSPPH_3577-R	CGGAGCTCGAATTCGGATCCTCATTTGGTAAAGCGTCC
pET28a-*hutC*-F	AGCAAATGGGTCGCGGATCCATGGGCGACTTCCACTAT
pET28a-*hutC*-R	CGGAGCTCGAATTCGGATCCTTACGCCTTCTTGTTGCG
pET28a-*fur*-F	AGCAAATGGGTCGCGGATCCATGCTTGTCGACCCACTG
pET28a-*fur*-R	CGGAGCTCGAATTCGGATCCTCAAACCTGCCCCCCGTG
pET28a-PSPPH_3297-F	AGCAAATGGGTCGCGGATCCATGCCTGTCGACATACAA
pET28a-PSPPH_3297-R	CGGAGCTCGAATTCGGATCCTTACCTGTCTGGATAACG
pET28a-PSPPH_1136-F	AGCAAATGGGTCGCGGATCCATGGTGATGCCCCACACCTCA
pET28a-PSPPH_1136-R	CGGAGCTCGAATTCGGATCCTCAGCTGGAAGGCGAAAC
pET28a-PSPPH_0286-F	AGCAAATGGGTCGCGGATCCATGCCCAATACTCCCCTG
pET28a-PSPPH_0286-R	CGGAGCTCGAATTCGGATCCTCAAGCCGCCTTGCAGCC
pET28a-PSPPH_0263-F	AGCAAATGGGTCGCGGATCCATGCCTATCGACCTCGA
pET28a-PSPPH_0263-R	CGGAGCTCGAATTCGGATCCTTAGGTCGGGTTGTTCA
pET28a-PSPPH_4056-F	AGCAAATGGGTCGCGGATCCATGCAAGAGCCTGTCAG
pET28a-PSPPH_4056-R	CGGAGCTCGAATTCGGATCCTTACTCCAGCGGCACC
pET28a-PSPPH_3031-F	AGCAAATGGGTCGCGGATCCATGCGACTGACTACTAAAGG
pET28a-PSPPH_3031-R	CGGAGCTCGAATTCGGATCCTTATTCGACGGCGGA
pET28a-*iscR*-F	AGCAAATGGGTCGCGGATCCATGAACGACCAGTCCCTG
pET28a-*iscR*-R	CGGAGCTCGAATTCGGATCCTTAACGCCCGGTCTTTTTC
pET28a-PSPPH_1771-F	AGCAAATGGGTCGCGGATCCATGGGGATCGCAGCCTA
pET28a-PSPPH_1771-R	CGGAGCTCGAATTCGGATCCTTAGTCGCTGTACAGCG
pET28a-PSPPH_3004-F	AGCAAATGGGTCGCGGATCCATGAACGACGCTGCTT
pET28a-PSPPH_3004-R	CGGAGCTCGAATTCGGATCCTTAGCGGCCCCGG
pET28a-PSPPH_0486-F	AGCAAATGGGTCGCGGATCCATGCTGGGTGCGGTC
pET28a-PSPPH_0486-R	CGGAGCTCGAATTCGGATCCTTATTCACCTCGGTCGGGA
pET28a-PSPPH_2055-F	AGCAAATGGGTCGCGGATCCATGGATGTTGAAGCCGCA
pET28a-PSPPH_2055-R	CGGAGCTCGAATTCGGATCCTTACACCCCAAACACCTTC
pET28a-PSPPH_1734-F	AGCAAATGGGTCGCGGATCCATGACGTTCAAGGCCC
pET28a-PSPPH_1734-R	CGGAGCTCGAATTCGGATCCTTAGTCCGCTGCCAG
pET28a-PSPPH_1776-F	AGCAAATGGGTCGCGGATCCATGAGTACGGTTATACTCAGC
pET28a-PSPPH_1776-R	CGGAGCTCGAATTCGGATCCTTAATCCTTTGCGCGCA
pET28a-PSPPH_3005-F	AGCAAATGGGTCGCGGATCCATGGAATACGGACCTTGCATGC
pET28a-PSPPH_3005-R	CGGAGCTCGAATTCGGATCCTTAGGCCGCCGCCG
pET28a-PSPPH_4637-F	AGCAAATGGGTCGCGGATCCATGACTCGAACTGCCAG
pET28a-PSPPH_4637-R	CGGAGCTCGAATTCGGATCCTTAGCGCCGTTTGAAAAT
pET28a-PSPPH_3471-F	AGCAAATGGGTCGCGGATCCATGCGTGAAGATAAACACCCT
pET28a-PSPPH_3471-R	CGGAGCTCGAATTCGGATCCTTATGCCGACTCCTCCG
pET28a-PSPPH_3923-F	AGCAAATGGGTCGCGGATCCATGCGCAAATCAGATAGAGA
pET28a-PSPPH_3923-R	CGGAGCTCGAATTCGGATCCTTAGGCGGGGGTTTTCT
pET28a-PSPPH_5087-F	AGCAAATGGGTCGCGGATCCATGAGTGAATCTGCCTCG
pET28a-PSPPH_5087-R	CGGAGCTCGAATTCGGATCCTTATTCGGGCGTGATCT
pET28a-PSPPH_2859-F	AGCAAATGGGTCGCGGATCCATGCCATTGACTGATGAACA
pET28a-PSPPH_2859-R	CGGAGCTCGAATTCGGATCCTTAGGATCTTTCCAGATTGG

pET28a-PSPPH_1519-F	AGCAAATGGGTCGCGGATCCATGCTTGACTTAAAAAAACCT
pET28a-PSPPH_1519-R	CGGAGCTCGAATTCGGATCCTTACTCGACGTCAGTCC
pET28a-PSPPH_1800-F	AGCAAATGGGTCGCGGATCCATGTCGTCAGCCGAAC
pET28a-PSPPH_1800-R	CGGAGCTCGAATTCGGATCCTTAGCTTTTACTGCCACTT
pET28a-PSPPH_1617-F	AGCAAATGGGTCGCGGATCCATGCAGTTGTCTAGACAAGC
pET28a-PSPPH_1617-R	CGGAGCTCGAATTCGGATCCTTAGTCAGTCGTTGGGGT
pET28a-*phnF*-F	AGCAAATGGGTCGCGGATCCATGAGCAAACCCGGGC
pET28a-*phnF*-R	CGGAGCTCGAATTCGGATCCTTAGTTCGAGCGCTGGAT
pET28a-*vanR*-F	AGCAAATGGGTCGCGGATCCATGGCCAAACAGCAATTATTG
pET28a-*vanR*-R	CGGAGCTCGAATTCGGATCCTTAACTCCAGCGCTTGA
pET28a-PSPPH_5194-F	AGCAAATGGGTCGCGGATCCATGCAGACAGCATGTGC
pET28a-PSPPH_5194-R	CGGAGCTCGAATTCGGATCCTTATTGTGTTTGCACACG
pET28a-PSPPH_2720-F	AGCAAATGGGTCGCGGATCCATGATTCTTGAGCAAATCAAAG
pET28a-PSPPH_2720-R	CGGAGCTCGAATTCGGATCCTTATAGTTCTTTCTTGAGCCG
pET28a-PSPPH_1222-F	AGCAAATGGGTCGCGGATCCATGCGCGACGATGTG
pET28a-PSPPH_1222-R	CGGAGCTCGAATTCGGATCCTTACAACTTGCCACGCA
pET28a-PSPPH_1595-F	AGCAAATGGGTCGCGGATCCATGCAAAACCAGAGTGCA
pET28a-PSPPH_1595-R	CGGAGCTCGAATTCGGATCCTTACGCTGCCGCTGA
pET28a-PSPPH_2423-F	AGCAAATGGGTCGCGGATCCATGAGTGCCGAAGAAAAC
pET28a-PSPPH_2423-R	CGGAGCTCGAATTCGGATCCTTAAAGGCTCTGGTGCAG
pET28a-*mgrA*-F	AGCAAATGGGTCGCGGATCCATGAAACATTTCAGCCCCG
pET28a-*mgrA*-R	CGGAGCTCGAATTCGGATCCTTAGCTTTTAGCTTTGGGGT
pET28a-*marR*-F	AGCAAATGGGTCGCGGATCCATGGAAAATCAAAGCGCTC
pET28a-*marR*-R	CGGAGCTCGAATTCGGATCCTTAGACCCGCTGCGA
pET28a-PSPPH_4253-F	AGCAAATGGGTCGCGGATCCATGAATTCCCCCAACACC
pET28a-PSPPH_4253-R	CGGAGCTCGAATTCGGATCCTTATGCTTCATGCCGC
pET28a-PSPPH_1233-F	AGCAAATGGGTCGCGGATCCATGGTTGCAGGCACC
pET28a-PSPPH_1233-R	CGGAGCTCGAATTCGGATCCTTAGCGCCCTATGCG
pET28a-PSPPH_2563-F	AGCAAATGGGTCGCGGATCCATGACGCCCAACGGC
pET28a-PSPPH_2563-R	CGGAGCTCGAATTCGGATCCTTAGCGAATTTCCACGCT
pET28a-PSPPH_3048-F	AGCAAATGGGTCGCGGATCCATGGCCAGAAGTCATCGT
pET28a-PSPPH_3048-R	CGGAGCTCGAATTCGGATCCTTAGCAGGTCTCGCC
pET28a-PSPPH_1456-F	AGCAAATGGGTCGCGGATCCATGGCCAAGCCATCCG
pET28a-PSPPH_1456-R	CGGAGCTCGAATTCGGATCCTTACGATTGGTAGACCTTTTTC
pET28a-PSPPH_2432-F	AGCAAATGGGTCGCGGATCCATGGTTATCGAAGAATCAGGGA
pET28a-PSPPH_2432-R	CGGAGCTCGAATTCGGATCCTTAGGGATGAGCCTTGAT
pET28a-PSPPH_2204-F	AGCAAATGGGTCGCGGATCCATGGCTGCAGGAATCCAC
pET28a-PSPPH_2204-R	CGGAGCTCGAATTCGGATCCTTAGGGTGAGAGCGTCA
pET28a-PSPPH_0755-F	AGCAAATGGGTCGCGGATCCATGTGCAGGGCGCAC
pET28a-PSPPH_0755-R	CGGAGCTCGAATTCGGATCCTTAGGCAGTACGCGG
pET28a-PSPPH_1049-F	AGCAAATGGGTCGCGGATCCATGTGCAGGGCGCAC
pET28a-PSPPH_1049-R	CGGAGCTCGAATTCGGATCCTTAGGCAGTACGCGG
pET28a-PSPPH_2106-F	AGCAAATGGGTCGCGGATCCATGACCCTTGAAGTCCCCG
pET28a-PSPPH_2106-R	CGGAGCTCGAATTCGGATCCTTACAAGGCTGGCGGC
pET28a-PSPPH_4622-F	AGCAAATGGGTCGCGGATCCATGACTCAATTGCCGGTAA
pET28a-PSPPH_4622-R	CGGAGCTCGAATTCGGATCCTTATTGCGGCAAACCAC

pET28a-PSPPH_2407-F	AGCAAATGGGTCGCGGATCCATGTCTGCATGGTCGG
pET28a-PSPPH_2407-R	CGGAGCTCGAATTCGGATCCTTAGCGCCCTGTCAG
pET28a-PSPPH_1730-F	AGCAAATGGGTCGCGGATCCATGAGCAGTATCCGCGAG
pET28a-PSPPH_1730-R	CGGAGCTCGAATTCGGATCCTTAGCGGTCCGGCT
pET28a-PSPPH_1728-F	AGCAAATGGGTCGCGGATCCATGGTTCGTCGCACCAAAG
pET28a-PSPPH_1728-R	CGGAGCTCGAATTCGGATCCTTAGGAAGCCTTGCTGATT
pET28a-PSPPH_4012-F	AGCAAATGGGTCGCGGATCCATGACCATCAACGCACGA
pET28a-PSPPH_4012-R	CGGAGCTCGAATTCGGATCCTTAAGCCGGCAGCGG
pET28a-PSPPH_3487-F	AGCAAATGGGTCGCGGATCCATGACCGCACCCATGC
pET28a-PSPPH_3487-R	CGGAGCTCGAATTCGGATCCTTAGGGAGTCACTTCGTA
pET28a-PSPPH_2448-F	AGCAAATGGGTCGCGGATCCATGAGAGTCACCAAGGC
pET28a-PSPPH_2448-R	CGGAGCTCGAATTCGGATCCTTAAAGAGCCTGCAGTTG
pET28a-PSPPH_3066-F	AGCAAATGGGTCGCGGATCCATGACTCTGCATGACCCCA
pET28a-PSPPH_3066-R	CGGAGCTCGAATTCGGATCCTTAGGGACGAGCCCTTA
pET28a-PSPPH_3654-F	AGCAAATGGGTCGCGGATCCATGCGTTACGCGCTC
pET28a-PSPPH_3654-R	CGGAGCTCGAATTCGGATCCTTAGTTGGCGCTCTTC
pET28a-PSPPH_2305-F	AGCAAATGGGTCGCGGATCCATGGCACAGATGGGACG
pET28a-PSPPH_2305-R	CGGAGCTCGAATTCGGATCCTTATTGGTCGTCGGCAA
pET28a-PSPPH_2893-F	AGCAAATGGGTCGCGGATCCATGAGCCTCAATCATTATTGC
pET28a-PSPPH_2893-R	CGGAGCTCGAATTCGGATCCTTATGATTTCTGACCTGCAGG
pET28a-PSPPH_1097-F	AGCAAATGGGTCGCGGATCCATGCCCGCCATTGAATC
pET28a-PSPPH_1097-R	CGGAGCTCGAATTCGGATCCTTATTGGCCTAACGCCAG
pET28a-PSPPH_3122-F	AGCAAATGGGTCGCGGATCCATGATCGTCCTGAACTCGA
pET28a-PSPPH_3122-R	CGGAGCTCGAATTCGGATCCTTACCCGTCGACGCTTTTC
pET28a-PSPPH_3486-F	AGCAAATGGGTCGCGGATCCATGTTTCTAAGCCTTGAAGTTC
pET28a-PSPPH_3486-R	CGGAGCTCGAATTCGGATCCTTACCCGGACTGTTTTTGC
pET28a-PSPPH_2597-F	AGCAAATGGGTCGCGGATCCATGAGTAAAACAACAAGAATCGC
pET28a-PSPPH_2597-R	CGGAGCTCGAATTCGGATCCTTAGTACAGGTTTTGTTCGG
pET28a-PSPPH_1962-F	AGCAAATGGGTCGCGGATCCATGACGTTACCTGTTGTTATTG
pET28a-PSPPH_1962-R	CGGAGCTCGAATTCGGATCCTTAAACCCTACAGCCCTCTT
pET28a-PSPPH_3027-F	AGCAAATGGGTCGCGGATCCATGCTGCGCATGGC
pET28a-PSPPH_3027-R	CGGAGCTCGAATTCGGATCCTTAGTCGTGACGAGTGATC
pET28a-PSPPH_2994-F	AGCAAATGGGTCGCGGATCCATGACTCTCAAAAGTGACTG
pET28a-PSPPH_2994-R	CGGAGCTCGAATTCGGATCCTTAGTCGAAATTGAACAGG
pET28a-PSPPH_2214-F	AGCAAATGGGTCGCGGATCCATGGCTATCAAAGAAGCAG
pET28a-PSPPH_2214-R	CGGAGCTCGAATTCGGATCCTTAGCTCAGGCAGCG
pET28a-PSPPH_4495-F	AGCAAATGGGTCGCGGATCCATGCTGCCCCGCG
pET28a-PSPPH_4495-R	CGGAGCTCGAATTCGGATCCTTAATCGTGACTGCCGA
pET28a-PSPPH_3202-F	AGCAAATGGGTCGCGGATCCATGGCCCAGTCGGAAAC
pET28a-PSPPH_3202-R	CGGAGCTCGAATTCGGATCCTTAGGCCTTGGCTACCG
pET28a-PSPPH_2733-F	AGCAAATGGGTCGCGGATCCATGGCCAGGCTGAAAACT
pET28a-PSPPH_2733-R	CGGAGCTCGAATTCGGATCCTTATTTGTCAGGTGCAGG
pET28a-PSPPH_1670-F	AGCAAATGGGTCGCGGATCCATGCCGACCTTGACCC
pET28a-PSPPH_1670-R	CGGAGCTCGAATTCGGATCCTTATGCGCTTTGGCG
pET28a-PSPPH_3479-F	AGCAAATGGGTCGCGGATCCATGTCGACCTCAGATACC
pET28a-PSPPH_3479-R	CGGAGCTCGAATTCGGATCCTTATGGTGTAAGCAAAAAAGA

pET28a-PSPPH_4788-F	AGCAAATGGGTCGCGGATCCATGGCAACCCGTACCAA
pET28a-PSPPH_4788-R	CGGAGCTCGAATTCGGATCCTTACATCAGAACCTCAGG
pET28a-PSPPH_4320-F	AGCAAATGGGTCGCGGATCCATGAGCGCTCTGGACAT
pET28a-PSPPH_4320-R	CGGAGCTCGAATTCGGATCCTTAATTTTCTTGTGCGGTTTC
pET28a-PSPPH_0376-F	AGCAAATGGGTCGCGGATCCATGAAAACCCGCGACC
pET28a-PSPPH_0376-R	CGGAGCTCGAATTCGGATCCTTACCTACCCAGATACTCCAT
pET28a-PSPPH_0735-F	AGCAAATGGGTCGCGGATCCATGAGGAACGATAAGTGTCT
pET28a-PSPPH_0735-R	CGGAGCTCGAATTCGGATCCTTAAGGCGACAGACTGC
pET28a-PSPPH_3268-F	AGCAAATGGGTCGCGGATCCATGAAGGTGCGTACTGAAG
pET28a-PSPPH_3268-R	CGGAGCTCGAATTCGGATCCTCAGCGCTTTTCCATGCC
pET28a-PSPPH_4899-F	AGCAAATGGGTCGCGGATCCATGACACGCGCCGCCACG
pET28a-PSPPH_4899-R	CGGAGCTCGAATTCGGATCCTTAAATAACTCGCTTCAC
pET28a-PSPPH_4448-F	AGCAAATGGGTCGCGGATCCATGACGATGATGACCGAG
pET28a-PSPPH_4448-R	CGGAGCTCGAATTCGGATCCTTAAAGCAGATCGTACTG
pET28a-PSPPH_1651-F	AGCAAATGGGTCGCGGATCCATGCAGAAAGAACCTCGT
pET28a-PSPPH_1651-R	CGGAGCTCGAATTCGGATCCTTATTCCGTAGCAGGGTC
pET28a-PSPPH_2984-F	AGCAAATGGGTCGCGGATCCTTGCCACTGCCTCAACTG
pET28a-PSPPH_2984-R	CGGAGCTCGAATTCGGATCCTTATCCGCTCGCATCGGA
pET28a-PSPPH_3872-F	AGCAAATGGGTCGCGGATCCGTGACGATTGCCAGAGAAG
pET28a-PSPPH_3872-R	CGGAGCTCGAATTCGGATCCTCACCCTCGAACCGGCTG
pET28a-PSPPH_2774-F	AGCAAATGGGTCGCGGATCCATGAAGCTCATTGCTGAC
pET28a-PSPPH_2774-R	CGGAGCTCGAATTCGGATCCTCAGGAGGTTGACAGGCC
pET28a-PSPPH_3983-F	AGCAAATGGGTCGCGGATCCATGAACGTGGCCACTCCCT
pET28a-PSPPH_3983-R	CGGAGCTCGAATTCGGATCCCTATCCCAACTCTACTCC
pET28a-PSPPH_2905-F	AGCAAATGGGTCGCGGATCCTTGAAGACGTACCCGATG
pET28a-PSPPH_2905-R	CGGAGCTCGAATTCGGATCCTCACTTGCCAGTCGTCGC
pET28a-*betI*-F	AGCAAATGGGTCGCGGATCCATGCCCAAGGTCGGAATG
pET28a-*betI*-R	CGGAGCTCGAATTCGGATCCTCAGCTCGCCGATTTCGC
pET28a-PSPPH_0102-F	AGCAAATGGGTCGCGGATCCATGTCTAGTCGACGATAT
pET28a-PSPPH_0102-R	CGGAGCTCGAATTCGGATCCTTATTGATCGGGAGTGGG
pET28a-PSPPH_0101-F	AGCAAATGGGTCGCGGATCCATGACCGGCCCATCAACT
pET28a-PSPPH_0101-R	CGGAGCTCGAATTCGGATCCTTACAAACCTCCTCCATTC
pET28a-PSPPH_4920-F	AGCAAATGGGTCGCGGATCCATGTATCAATATCCATTG
pET28a-PSPPH_4920-R	CGGAGCTCGAATTCGGATCCTCAAGCGGCCCGCTCAAT
pET28a-PSPPH_3182-F	AGCAAATGGGTCGCGGATCCATGACAAAGGAAAAGTTG
pET28a-PSPPH_3182-R	CGGAGCTCGAATTCGGATCCTCAGGCCTGGCGCTGGTG
pET28a-*dksA2*-F	AGCAAATGGGTCGCGGATCCATGCCCACCCCAGAAAAG
pET28a-*dksA2*-R	CGGAGCTCGAATTCGGATCCTCACTTACCGACCTGTTTTTC
pET28a-PSPPH_5139-F	AGCAAATGGGTCGCGGATCCATGAAAATTATAAAAAATG
pET28a-PSPPH_5139-R	CGGAGCTCGAATTCGGATCCTCAAAACGTTCTCCCTGC
pET28a-PSPPH_4336-F	AGCAAATGGGTCGCGGATCCATGATTCTCTGCCTGAAG
pET28a-PSPPH_4336-R	CGGAGCTCGAATTCGGATCCTCAGAACACCCCGCTCAG
pET28a-*sfsA*-F	AGCAAATGGGTCGCGGATCCATGCGTTTTGCTCCGCAG
pET28a-*sfsA*-R	CGGAGCTCGAATTCGGATCCTCAATCCAGCCAGTGCAC
pET28a-*algQ*-F	AGCAAATGGGTCGCGGATCCATGCTGGAAAGTTGTCAG
pET28a-*algQ*-R	CGGAGCTCGAATTCGGATCCTCACACCTGCGCGGCGAT

Supplementary Table 1b: Strains, plasmids and primers used in this study, related to Fig. 1a, Fig. 2c-2e, Supplementary Fig.2b-2F, Supplementary Fig. 3

**Strains**	**Description**	**Reference or Application**
***E. coli* Strains**		
E. coli DH5α	supE44 Δlac*U169*(φ80lacZΔM15)*hsdR17recA1 endA1 gyrA96thi-1 relA1*λpir	Stratagene
E. coli BL21(DE3)	*F⁻ ompT hsdS*$_B$ *(r*$_B$$^-$*m*$_B$$^-$*) gal dcm met* (DE3)	Invitrogen
***P. syringae* strains**		
P. syringae 1448A	Wild type	Lab stock
P. syringae 1448A Δ*metR*	Clean deletion of *metR*	This study
P. syringae 1448A ΔPSPPH_3297	Clean deletion of PSPPH_3297	This study
P. syringae 1448A Δ*pcaQ*	Clean deletion of *pcaQ*	This study
P. syringae 1448A Δ*rbsR*	Clean deletion of *rbsR*	This study

**Plasmids**	**Description**	**Source or reference**
**pMS402**	Expression reporter plasmid carrying the promoterless luxCDABE; Kn^r	Duan et al., 2003
pMS402-2621	*lux*-reporter fused with the promoter of PSPPH_2621; Kn^r	This study
pMS402-3297	*lux*-reporter fused with the promoter of PSPPH_3297; Kn^r	This study
**pK18 mobsacB**	a pK18 suicide plasmid, used to mutant gene in *P. syringae* 1448A	Kvitko *et al.,*2011
pK18-*metR*	This plasmid was used to mutate *metR* in *P. syringae* 1448A	This study
pK18-PSPPH_3297	This plasmid was used to mutate PSPPH_3297 in *P. syringae* 1448A	This study
pK18-*pcaQ*	This plasmid was used to mutate *pcaQ* in *P. syringae* 1448A	This study
pK18-*rbsR*	This plasmid was used to mutate *rbsR* in *P. syringae* 1448A	This study
**pET28a**	N-terminus His-tag fusion expression plasmid. Ori:f1. T7 promoter. Kan^r	Invitrogen
pET28a-PSPPH_4238	pET28a containing ORF of PSPPH_4238 from *P. syringae* 1448A	This study
pET28a-PSPPH_1818	pET28a containing ORF of PSPPH_1818 from *P. syringae* 1448A	This study
pET28a-PSPPH_1615	pET28a containing ORF of PSPPH_1615 from *P. syringae* 1448A	This study
pET28a-PSPPH_3285	pET28a containing ORF of PSPPH_3285 from *P. syringae* 1448A	This study
pET28a-PSPPH_3530	pET28a containing ORF of PSPPH_3530 from P. syringae 1448A	This study
pET28a-PSPPH_0082	pET28a containing ORF of PSPPH_0082 from *P. syringae* 1448A	This study

pET28a-PSPPH_2337	pET28a containing ORF of PSPPH_2337 from *P. syringae* 1448A	This study
pET28a-PSPPH_1169	pET28a containing ORF of PSPPH_1169 from *P. syringae* 1448A	This study
pET28a-PSPPH_2872	pET28a containing ORF of PSPPH_2872 from *P. syringae* 1448A	This study
pET28a-PSPPH_2357	pET28a containing ORF of PSPPH_2357 from *P. syringae* 1448A	This study
pET28a-PSPPH_0406	pET28a containing ORF of PSPPH_0406 from *P. syringae* 1448A	This study
pET28a-PSPPH_5145	pET28a containing ORF of PSPPH_5145 from *P. syringae* 1448A	This study
pET28a-*hexR*	pET28a containing ORF of *hexR* from *P. syringae* 1448A	This study
pET28a-PSPPH_3195	pET28a containing ORF of PSPPH_3195 from *P. syringae* 1448A	This study
pET28a-PSPPH_1680	pET28a containing ORF of PSPPH_1680 from *P. syringae* 1448A	This study
pET28a-*hrpR*	pET28a containing ORF of *hrpR* truncation (from 241 to 306 aa) from *P. syringae* 1448A	Wang *et al*., 2018
pET28a-PSPPH_2426	pET28a containing ORF of PSPPH_2426 from *P. syringae* 1448A	This study
pET28a-PSPPH_3168	pET28a containing ORF of PSPPH_3168 from *P. syringae* 1448A	This study
pET28a-PSPPH_2229	pET28a containing ORF of PSPPH_2229 from *P. syringae* 1448A	This study
pET28a-*hrpS*	pET28a containing ORF of *hrpS* truncation (from 235 to 303 aa) from *P. syringae* 1448A	Wang *et al*., 2018
pET28a-PSPPH_3522	pET28a containing ORF of PSPPH_3522 from *P. syringae* 1448A	This study
pET28a-PSPPH_4744	pET28a containing ORF of PSPPH_4744 from *P. syringae* 1448A	This study
pET28a-PSPPH_4127	pET28a containing ORF of PSPPH_4127 from *P. syringae* 1448A	This study
pET28a-*ftrA*	pET28a containing ORF of *ftrA* from *P. syringae* 1448A	This study
pET28a-*ntrC*	pET28a containing ORF of *ntrC* from *P. syringae* 1448A	This study
pET28a-*fleQ*	pET28a containing ORF of *fleQ* from *P. syringae* 1448A	This study
pET28a-*algB*	pET28a containing ORF of *algB* from *P. syringae* 1448A	This study
pET28a-*tsiR*	pET28a containing ORF of *tsiR* from *P. syringae* 1448A	This study
pET28a-PSPPH_0146	pET28a containing ORF of PSPPH_0146 from *P. syringae* 1448A	This study
pET28a-*pilR*	pET28a containing ORF of *pilR* from *P. syringae* 1448A	This study
pET28a-*fleR*	pET28a containing ORF of *fleR* from *P. syringae* 1448A	This study
pET28a-PSPPH_3907	pET28a containing ORF of PSPPH_3907 from *P. syringae* 1448A	This study
pET28a-PSPPH_4002	pET28a containing ORF of PSPPH_4002 from *P. syringae* 1448A	This study

pET28a-PSPPH_5171	pET28a containing ORF of PSPPH_5171 from *P. syringae* 1448A	This study
pET28a-PSPPH_3800	pET28a containing ORF of PSPPH_3800 from *P. syringae* 1448A	This study
pET28a-PSPPH_4241	pET28a containing ORF of PSPPH_4241 from *P. syringae* 1448A	This study
pET28a-PSPPH_0642	pET28a containing ORF of PSPPH_0642 from *P. syringae* 1448A	This study
pET28a-PSPPH_3220	pET28a containing ORF of PSPPH_3220 from *P. syringae* 1448A	This study
pET28a-PSPPH_1364	pET28a containing ORF of PSPPH_1364 from *P. syringae* 1448A	This study
pET28a-PSPPH_1374	pET28a containing ORF of PSPPH_1374 from *P. syringae* 1448A	This study
pET28a-*gacA*	pET28a containing ORF of *gacA* from *P. syringae* 1448A	This study
pET28a-*cpxR*	pET28a containing ORF of *cpxR* from *P. syringae* 1448A	This study
pET28a-PSPPH_0778	pET28a containing ORF of PSPPH_0778 from *P. syringae* 1448A	This study
pET28a-PSPPH_1906	pET28a containing ORF of PSPPH_1906 from *P. syringae* 1448A	This study
pET28a-*rhpR*	pET28a containing ORF of *rhpR* from *P. syringae* 1448A	Xie *et al*., 2019
pET28a-PSPPH_4419	pET28a containing ORF of PSPPH_4419 from *P. syringae* 1448A	This study
pET28a-*kdpE*	pET28a containing ORF of *kdpE* from *P. syringae* 1448A	This study
pET28a-*cvsR*	pET28a containing ORF of *cvsR* from *P. syringae* 1448A	This study
pET28a-*colR*	pET28a containing ORF of *colR* from *P. syringae* 1448A	This study
pET28a-*baeS1*	pET28a containing ORF of *baeS1* from *P. syringae* 1448A	This study
pET28a-*algR*	pET28a containing ORF of *algR* from *P. syringae* 1448A	This study
pET28a-PSPPH_3547	pET28a containing ORF of PSPPH_3547 from *P. syringae* 1448A	This study
pET28a-PSPPH_3040	pET28a containing ORF of PSPPH_3040 from *P. syringae* 1448A	This study
pET28a-PSPPH_1195	pET28a containing ORF of PSPPH_1195 from *P. syringae* 1448A	This study
pET28a-PSPPH_3453	pET28a containing ORF of PSPPH_3453 from *P. syringae* 1448A	This study
pET28a-PSPPH_4828	pET28a containing ORF of PSPPH_4828 from *P. syringae* 1448A	This study
pET28a-*ompR*	pET28a containing ORF of *ompR* from *P. syringae* 1448A	This study
pET28a-*tctD*	pET28a containing ORF of *tctD* from *P. syringae* 1448A	This study
pET28a-PSPPH_3294	pET28a containing ORF of PSPPH_3294 from *P. syringae* 1448A	This study
pET28a-PSPPH_2995	pET28a containing ORF of PSPPH_2995 from *P. syringae* 1448A	This study
pET28a-*gltR*	pET28a containing ORF of *gltR* from *P. syringae* 1448A	This study

pET28a-*phoB*	pET28a containing ORF of *phoB* from *P. syringae* 1448A	This study
pET28a-*phoP*	pET28a containing ORF of *phoP* from *P. syringae* 1448A	This study
pET28a-*rstA*	pET28a containing ORF of *rstA* from *P. syringae* 1448A	This study
pET28a-PSPPH_4255	pET28a containing ORF of PSPPH_4255 from *P. syringae* 1448A	This study
pET28a-PSPPH_4841	pET28a containing ORF of PSPPH_4841 from *P. syringae* 1448A	This study
pET28a-PSPPH_4692	pET28a containing ORF of PSPPH_4692 from *P. syringae* 1448A	This study
pET28a-PSPPH_2862	pET28a containing ORF of PSPPH_2862 from *P. syringae* 1448A	This study
pET28a-PSPPH_1580	pET28a containing ORF of PSPPH_1580 from *P. syringae* 1448A	This study
pET28a-*anr*	pET28a containing ORF of *anr* from *P. syringae* 1448A	This study
pET28a-*vfr*	pET28a containing ORF of *vfr* from *P. syringae* 1448A	This study
pET28a-PSPPH_4646	pET28a containing ORF of PSPPH_4646 from *P. syringae* 1448A	This study
pET28a-*pobR*	pET28a containing ORF of *pobR* from *P. syringae* 1448A	This study
pET28a-PSPPH_2917	pET28a containing ORF of PSPPH_2917 from *P. syringae* 1448A	This study
pET28a-PSPPH_3170	pET28a containing ORF of PSPPH_3170 from *P. syringae* 1448A	This study
pET28a-PSPPH_1160	pET28a containing ORF of PSPPH_1160 from *P. syringae* 1448A	This study
pET28a-*cspD*	pET28a containing ORF of *cspD* from *P. syringae* 1448A	This study
pET28a-*capB*	pET28a containing ORF of *capB* from *P. syringae* 1448A	This study
pET28a-*capA*	pET28a containing ORF of *capA* from *P. syringae* 1448A	This study
pET28a-PSPPH_3779	pET28a containing ORF of PSPPH_3779 from *P. syringae* 1448A	This study
pET28a-PSPPH_3504	pET28a containing ORF of PSPPH_3504 from *P. syringae* 1448A	This study
pET28a-PSPPH_2338	pET28a containing ORF of PSPPH_2338 from *P. syringae* 1448A	This study
pET28a-*gntR*	pET28a containing ORF of *gntR* from *P. syringae* 1448A	This study
pET28a-*rbsR*	pET28a containing ORF of *rbsR* from *P. syringae* 1448A	This study
pET28a-*fruR*	pET28a containing ORF of *fruR* from *P. syringae* 1448A	This study
pET28a-PSPPH_5193	pET28a containing ORF of PSPPH_5193 from *P. syringae* 1448A	This study
pET28a-PSPPH_4730	pET28a containing ORF of PSPPH_4730 from *P. syringae* 1448A	This study
pET28a-PSPPH_4858	pET28a containing ORF of PSPPH_4858 from *P. syringae* 1448A	This study
pET28a-PSPPH_4673	pET28a containing ORF of PSPPH_4673 from *P. syringae* 1448A	This study

pET28a-PSPPH_1393	pET28a containing ORF of PSPPH_1393 from *P. syringae* 1448A	This study
pET28a-PSPPH_0653	pET28a containing ORF of PSPPH_0653 from *P. syringae* 1448A	This study
pET28a-PSPPH_2151	pET28a containing ORF of PSPPH_2151 from *P. syringae* 1448A	This study
pET28a-PSPPH_2478	pET28a containing ORF of PSPPH_2478 from *P. syringae* 1448A	This study
pET28a-PSPPH_0226	pET28a containing ORF of PSPPH_0226 from *P. syringae* 1448A	This study
pET28a-PSPPH_5057	pET28a containing ORF of PSPPH_5057 from *P. syringae* 1448A	This study
pET28a-PSPPH_4324	pET28a containing ORF of PSPPH_4324 from *P. syringae* 1448A	This study
pET28a-PSPPH_1584	pET28a containing ORF of PSPPH_1584 from *P. syringae* 1448A	This study
pET28a-PSPPH_4694	pET28a containing ORF of PSPPH_4694 from *P. syringae* 1448A	This study
pET28a-PSPPH_2792	pET28a containing ORF of PSPPH_2792 from *P. syringae* 1448A	This study
pET28a-PSPPH_2327	pET28a containing ORF of PSPPH_2327 from *P. syringae* 1448A	This study
pET28a-PSPPH_2194	pET28a containing ORF of PSPPH_2194 from P. syringae 1448A	This study
pET28a-PSPPH_4674	pET28a containing ORF of PSPPH_4674 from *P. syringae* 1448A	This study
pET28a-PSPPH_2800	pET28a containing ORF of PSPPH_2800 from *P. syringae* 1448A	This study
pET28a-PSPPH_4644	pET28a containing ORF of PSPPH_4644 from *P. syringae* 1448A	This study
pET28a-PSPPH_2142	pET28a containing ORF of PSPPH_2142 from *P. syringae* 1448A	This study
pET28a-PSPPH_1011	pET28a containing ORF of PSPPH_1011 from *P. syringae* 1448A	This study
pET28a-PSPPH_2630	pET28a containing ORF of PSPPH_2630 from *P. syringae* 1448A	This study
pET28a-*cadR*	pET28a containing ORF of *cadR* from *P. syringae* 1448A	This study
pET28a-PSPPH_2539	pET28a containing ORF of PSPPH_2539 from *P. syringae* 1448A	This study
pET28a-PSPPH_2015	pET28a containing ORF of PSPPH_2015 from *P. syringae* 1448A	This study
pET28a-PSPPH_2149	pET28a containing ORF of PSPPH_2149 from *P. syringae* 1448A	This study
pET28a-*iclR*	pET28a containing ORF of *iclR* from *P. syringae* 1448A	This study
pET28a-*pcaR*	pET28a containing ORF of *pcaR* from *P. syringae* 1448A	This study
pET28a-PSPPH_1960	pET28a containing ORF of PSPPH_1960 from *P. syringae* 1448A	This study
pET28a-PSPPH_2665	pET28a containing ORF of PSPPH_2665 from *P. syringae* 1448A	This study
pET28a-PSPPH_2832	pET28a containing ORF of PSPPH_2832 from *P. syringae* 1448A	This study
pET28a-PSPPH_4612	pET28a containing ORF of PSPPH_4612 from *P. syringae* 1448A	This study

pET28a-PSPPH_5042	pET28a containing ORF of PSPPH_5042 from *P. syringae* 1448A	This study
pET28a-PSPPH_4377	pET28a containing ORF of PSPPH_4377 from *P. syringae* 1448A	This study
pET28a-PSPPH_2219	pET28a containing ORF of PSPPH_2219 from *P. syringae* 1448A	This study
pET28a-PSPPH_1244	pET28a containing ORF of PSPPH_1244 from *P. syringae* 1448A	This study
pET28a-PSPPH_1762	pET28a containing ORF of PSPPH_1762 from *P. syringae* 1448A	This study
pET28a-PSPPH_5054	pET28a containing ORF of PSPPH_5054 from *P. syringae* 1448A	This study
pET28a-PSPPH_4700	pET28a containing ORF of PSPPH_4700 from *P. syringae* 1448A	This study
pET28a-PSPPH_4356	pET28a containing ORF of PSPPH_4356 from *P. syringae* 1448A	This study
pET28a-*lrp*	pET28a containing ORF of *lrp* from *P. syringae* 1448A	This study
pET28a-PSPPH_0239	pET28a containing ORF of PSPPH_0239 from *P. syringae* 1448A	This study
pET28a-PSPPH_3155	pET28a containing ORF of PSPPH_3155 from *P. syringae* 1448A	This study
pET28a-PSPPH_2921	pET28a containing ORF of PSPPH_2921 from *P. syringae* 1448A	This study
pET28a-PSPPH_3817	pET28a containing ORF of PSPPH_3817 from *P. syringae* 1448A	This study
pET28a-PSPPH_2301	pET28a containing ORF of PSPPH_2301 from *P. syringae* 1448A	This study
pET28a-PSPPH_3022	pET28a containing ORF of PSPPH_3022 from *P. syringae* 1448A	This study
pET28a-*iciA*	pET28a containing ORF of *iciA* from *P. syringae* 1448A	This study
pET28a-PSPPH_3079	pET28a containing ORF of PSPPH_3079 from *P. syringae* 1448A	This study
pET28a-PSPPH_3464	pET28a containing ORF of PSPPH_3464 from *P. syringae* 1448A	This study
pET28a-*metR*	pET28a containing ORF of *metR* from *P. syringae* 1448A	This study
pET28a-PSPPH_0141	pET28a containing ORF of PSPPH_0141 from *P. syringae* 1448A	This study
pET28a-PSPPH_3233	pET28a containing ORF of PSPPH_3233 from *P. syringae* 1448A	This study
pET28a-PSPPH_3990	pET28a containing ORF of PSPPH_3990 from *P. syringae* 1448A	This study
pET28a-PSPPH_3460	pET28a containing ORF of PSPPH_3460 from *P. syringae* 1448A	This study
pET28a-PSPPH_1100	pET28a containing ORF of PSPPH_1100 from *P. syringae* 1448A	This study
pET28a-PSPPH_2439	pET28a containing ORF of PSPPH_2439 from *P. syringae* 1448A	This study
pET28a-*pcaQ*	pET28a containing ORF of *pcaQ* from *P. syringae* 1448A	This study
pET28a-*trpI*	pET28a containing ORF of *trpI* from *P. syringae* 1448A	This study
pET28a-PSPPH_2555	pET28a containing ORF of PSPPH_2555 from *P. syringae* 1448A	This study

pET28a-PSPPH_3611	pET28a containing ORF of PSPPH_3611 from *P. syringae* 1448A	This study
pET28a-PSPPH_0711	pET28a containing ORF of PSPPH_0711 from *P. syringae* 1448A	This study
pET28a-*mexT*	pET28a containing ORF of *mexT* from *P. syringae* 1448A	This study
pET28a-PSPPH_2469	pET28a containing ORF of PSPPH_2469 from *P. syringae* 1448A	This study
pET28a-PSPPH_4620	pET28a containing ORF of PSPPH_4620 from *P. syringae* 1448A	This study
pET28a-PSPPH_2772	pET28a containing ORF of PSPPH_2772 from *P. syringae* 1448A	This study
pET28a-PSPPH_2638	pET28a containing ORF of PSPPH_2638 from *P. syringae* 1448A	This study
pET28a-*cysB*	pET28a containing ORF of *cysB* from *P. syringae* 1448A	This study
pET28a-PSPPH_2693	pET28a containing ORF of PSPPH_2693 from *P. syringae* 1448A	This study
pET28a-PSPPH_1493	pET28a containing ORF of PSPPH_1493 from *P. syringae* 1448A	This study
pET28a-PSPPH_4998	pET28a containing ORF of PSPPH_4998 from *P. syringae* 1448A	This study
pET28a-PSPPH_1259	pET28a containing ORF of PSPPH_1259 from *P. syringae* 1448A	This study
pET28a-PSPPH_3798	pET28a containing ORF of PSPPH_3798 from *P. syringae* 1448A	This study
pET28a-PSPPH_2444	pET28a containing ORF of PSPPH_2444 from *P. syringae* 1448A	This study
pET28a-*oxyR*	pET28a containing ORF of *oxyR* from *P. syringae* 1448A	This study
pET28a-PSPPH_4638	pET28a containing ORF of PSPPH_4638 from *P. syringae* 1448A	This study
pET28a-PSPPH_0700	pET28a containing ORF of PSPPH_0700 from *P. syringae* 1448A	This study
pET28a-PSPPH_1435	pET28a containing ORF of PSPPH_1435 from *P. syringae* 1448A	This study
pET28a-PSPPH_2193	pET28a containing ORF of PSPPH_2193 from *P. syringae* 1448A	This study
pET28a-PSPPH_5144	pET28a containing ORF of PSPPH_5144 from *P. syringae* 1448A	This study
pET28a-PSPPH_3411	pET28a containing ORF of PSPPH_3411 from *P. syringae* 1448A	This study
pET28a-PSPPH_3278	pET28a containing ORF of PSPPH_3278 from *P. syringae* 1448A	This study
pET28a-PSPPH_2688	pET28a containing ORF of PSPPH_2688 from *P. syringae* 1448A	This study
pET28a-PSPPH_1737	pET28a containing ORF of PSPPH_1737 from *P. syringae* 1448A	This study
pET28a-PSPPH_1951	pET28a containing ORF of PSPPH_1951 from *P. syringae* 1448A	This study
pET28a-PSPPH_1758	pET28a containing ORF of PSPPH_1758 from *P. syringae* 1448A	This study
pET28a-PSPPH_0442	pET28a containing ORF of PSPPH_0442 from *P. syringae* 1448A	This study
pET28a-PSPPH_3014	pET28a containing ORF of PSPPH_3014 from *P. syringae* 1448A	This study

pET28a-PSPPH_2580	pET28a containing ORF of PSPPH_2580 from *P. syringae* 1448A	This study
pET28a-PSPPH_3618	pET28a containing ORF of PSPPH_3618 from *P. syringae* 1448A	This study
pET28a-PSPPH_2631	pET28a containing ORF of PSPPH_2631 from *P. syringae* 1448A	This study
pET28a-PSPPH_0077	pET28a containing ORF of PSPPH_0077 from *P. syringae* 1448A	This study
pET28a-PSPPH_2476	pET28a containing ORF of PSPPH_2476 from *P. syringae* 1448A	This study
pET28a-PSPPH_2236	pET28a containing ORF of PSPPH_2236 from *P. syringae* 1448A	This study
pET28a-PSPPH_2967	pET28a containing ORF of PSPPH_2967 from *P. syringae* 1448A	This study
pET28a-PSPPH_0589	pET28a containing ORF of PSPPH_0589 from *P. syringae* 1448A	This study
pET28a-PSPPH_1579	pET28a containing ORF of PSPPH_1579 from *P. syringae* 1448A	This study
pET28a-PSPPH_2454	pET28a containing ORF of PSPPH_2454 from *P. syringae* 1448A	This study
pET28a-PSPPH_2553	pET28a containing ORF of PSPPH_2553 from *P. syringae* 1448A	This study
pET28a-PSPPH_3241	pET28a containing ORF of PSPPH_3241 from *P. syringae* 1448A	This study
pET28a-PSPPH_5132	pET28a containing ORF of PSPPH_5132 from *P. syringae* 1448A	This study
pET28a-PSPPH_2689	pET28a containing ORF of PSPPH_2689 from *P. syringae* 1448A	This study
pET28a-PSPPH_0411	pET28a containing ORF of PSPPH_0411 from *P. syringae* 1448A	This study
pET28a-PSPPH_2300	pET28a containing ORF of PSPPH_2300 from *P. syringae* 1448A	This study
pET28a-PSPPH_3468	pET28a containing ORF of PSPPH_3468 from *P. syringae* 1448A	This study
pET28a-PSPPH_2491	pET28a containing ORF of PSPPH_2491 from *P. syringae* 1448A	This study
pET28a-PSPPH_3643	pET28a containing ORF of PSPPH_3643 from *P. syringae* 1448A	This study
pET28a-PSPPH_0031	pET28a containing ORF of PSPPH_0031 from P. syringae 1448A	This study
pET28a-PSPPH_2295	pET28a containing ORF of PSPPH_2295 from *P. syringae* 1448A	This study
pET28a-PSPPH_0218	pET28a containing ORF of PSPPH_0218 from *P. syringae* 1448A	This study
pET28a-PSPPH_1969	pET28a containing ORF of PSPPH_1969 from *P. syringae* 1448A	This study
pET28a-PSPPH_2983	pET28a containing ORF of PSPPH_2983 from *P. syringae* 1448A	This study
pET28a-PSPPH_2315	pET28a containing ORF of PSPPH_2315 from *P. syringae* 1448A	This study
pET28a-PSPPH_5138	pET28a containing ORF of PSPPH_5138 from *P. syringae* 1448A	This study
pET28a-PSPPH_4822	pET28a containing ORF of PSPPH_4822 from *P. syringae* 1448A	This study
pET28a-*lexA1*	pET28a containing ORF of *lexA1* from *P. syringae* 1448A	This study

pET28a-*lexA2*	pET28a containing ORF of *lexA2* from *P. syringae* 1448A	This study
pET28a-PSPPH_3936	pET28a containing ORF of PSPPH_3936 from *P. syringae* 1448A	This study
pET28a-*glpR*	pET28a containing ORF of *glpR* from *P. syringae* 1448A	This study
pET28a-PSPPH_5204	pET28a containing ORF of PSPPH_5204 from *P. syringae* 1448A	This study
pET28a-PSPPH_4844	pET28a containing ORF of PSPPH_4844 from *P. syringae* 1448A	This study
pET28a-PSPPH_2188	pET28a containing ORF of PSPPH_2188 from *P. syringae* 1448A	This study
pET28a-*srl*	pET28a containing ORF of *srl* from *P. syringae* 1448A	This study
pET28a-PSPPH_3577	pET28a containing ORF of PSPPH_3577 from *P. syringae* 1448A	This study
pET28a-*hutC*	pET28a containing ORF of *hutC* from *P. syringae* 1448A	This study
pET28a-*fur*	pET28a containing ORF of *fur* from *P. syringae* 1448A	This study
pET28a-PSPPH_3297	pET28a containing ORF of PSPPH_3297 from *P. syringae* 1448A	This study
pET28a-PSPPH_1136	pET28a containing ORF of PSPPH_1136 from *P. syringae* 1448A	This study
pET28a-PSPPH_0286	pET28a containing ORF of PSPPH_0286 from *P. syringae* 1448A	This study
pET28a-PSPPH_0263	pET28a containing ORF of PSPPH_0263 from *P. syringae* 1448A	This study
pET28a-PSPPH_4056	pET28a containing ORF of PSPPH_4056 from *P. syringae* 1448A	This study
pET28a-PSPPH_3031	pET28a containing ORF of PSPPH_3031 from *P. syringae* 1448A	This study
pET28a-*iscR*	pET28a containing ORF of *iscR* from *P. syringae* 1448A	This study
pET28a-PSPPH_1771	pET28a containing ORF of PSPPH_1771 from *P. syringae* 1448A	This study
pET28a-PSPPH_3004	pET28a containing ORF of PSPPH_3004 from *P. syringae* 1448A	This study
pET28a-PSPPH_0486	pET28a containing ORF of PSPPH_0486 from P. syringae 1448A	This study
pET28a-PSPPH_2055	pET28a containing ORF of PSPPH_2055 from *P. syringae* 1448A	This study
pET28a-PSPPH_1734	pET28a containing ORF of PSPPH_1734 from *P. syringae* 1448A	This study
pET28a-PSPPH_1776	pET28a containing ORF of PSPPH_1776 from *P. syringae* 1448A	This study
pET28a-PSPPH_3005	pET28a containing ORF of PSPPH_3005 from *P. syringae* 1448A	This study
pET28a-PSPPH_4637	pET28a containing ORF of PSPPH_4637 from *P. syringae* 1448A	This study
pET28a-PSPPH_3471	pET28a containing ORF of PSPPH_3471 from *P. syringae* 1448A	This study
pET28a-PSPPH_3923	pET28a containing ORF of PSPPH_3923 from *P. syringae* 1448A	This study
pET28a-PSPPH_5087	pET28a containing ORF of PSPPH_5087 from *P. syringae* 1448A	This study

pET28a-PSPPH_2859	pET28a containing ORF of PSPPH_2859 from *P. syringae* 1448A	This study
pET28a-PSPPH_1519	pET28a containing ORF of PSPPH_1519 from *P. syringae* 1448A	This study
pET28a-PSPPH_1800	pET28a containing ORF of PSPPH_1800 from *P. syringae* 1448A	This study
pET28a-PSPPH_1617	pET28a containing ORF of PSPPH_1617 from *P. syringae* 1448A	This study
pET28a-*phnF*	pET28a containing ORF of *phnF* from *P. syringae* 1448A	This study
pET28a-*vanR*	pET28a containing ORF of *vanR* from *P. syringae* 1448A	This study
pET28a-PSPPH_5194	pET28a containing ORF of PSPPH_5194 from *P. syringae* 1448A	This study
pET28a-PSPPH_2720	pET28a containing ORF of PSPPH_2720 from *P. syringae* 1448A	This study
pET28a-PSPPH_1222	pET28a containing ORF of PSPPH_1222 from *P. syringae* 1448A	This study
pET28a-PSPPH_1595	pET28a containing ORF of PSPPH_1595 from *P. syringae* 1448A	This study
pET28a-PSPPH_2423	pET28a containing ORF of PSPPH_2423 from *P. syringae* 1448A	This study
pET28a-*mgrA*	pET28a containing ORF of *mgrA* from *P. syringae* 1448A	This study
pET28a-*marR*	pET28a containing ORF of *marR* from *P. syringae* 1448A	This study
pET28a-PSPPH_4253	pET28a containing ORF of PSPPH_4253 from *P. syringae* 1448A	This study
pET28a-PSPPH_1233	pET28a containing ORF of PSPPH_1233 from *P. syringae* 1448A	This study
pET28a-PSPPH_2563	pET28a containing ORF of PSPPH_2563 from *P. syringae* 1448A	This study
pET28a-PSPPH_3048	pET28a containing ORF of PSPPH_3048 from *P. syringae* 1448A	This study
pET28a-PSPPH_1456	pET28a containing ORF of PSPPH_1456 from *P. syringae* 1448A	This study
pET28a-PSPPH_2432	pET28a containing ORF of PSPPH_2432 from *P. syringae* 1448A	This study
pET28a-PSPPH_2204	pET28a containing ORF of PSPPH_2204 from *P. syringae* 1448A	This study
pET28a-PSPPH_0755	pET28a containing ORF of PSPPH_0755 from *P. syringae* 1448A	This study
pET28a-PSPPH_1049	pET28a containing ORF of PSPPH_1049 from *P. syringae* 1448A	This study
pET28a-PSPPH_2106	pET28a containing ORF of PSPPH_2106 from *P. syringae* 1448A	This study
pET28a-PSPPH_4622	pET28a containing ORF of PSPPH_4622 from *P. syringae* 1448A	This study
pET28a-PSPPH_2407	pET28a containing ORF of PSPPH_2407 from *P. syringae* 1448A	This study
pET28a-PSPPH_1730	pET28a containing ORF of PSPPH_1730 from *P. syringae* 1448A	This study
pET28a-PSPPH_1728	pET28a containing ORF of PSPPH_1728 from *P. syringae* 1448A	This study
pET28a-PSPPH_4012	pET28a containing ORF of PSPPH_4012 from *P. syringae* 1448A	This study

pET28a-PSPPH_3487	pET28a containing ORF of PSPPH_3487 from *P. syringae* 1448A	This study
pET28a-*aefR*	pET28a containing ORF of *aefR* from *P. syringae* 1448A	This study
pET28a-PSPPH_2448	pET28a containing ORF of PSPPH_2448 from *P. syringae* 1448A	This study
pET28a-PSPPH_3066	pET28a containing ORF of PSPPH_3066 from *P. syringae* 1448A	This study
pET28a-PSPPH_3654	pET28a containing ORF of PSPPH_3654 from *P. syringae* 1448A	This study
pET28a-PSPPH_2305	pET28a containing ORF of PSPPH_2305 from *P. syringae* 1448A	This study
pET28a-PSPPH_2893	pET28a containing ORF of PSPPH_2893 from *P. syringae* 1448A	This study
pET28a-PSPPH_1097	pET28a containing ORF of PSPPH_1097 from *P. syringae* 1448A	This study
pET28a-PSPPH_3122	pET28a containing ORF of PSPPH_3122 from *P. syringae* 1448A	This study
pET28a-PSPPH_3486	pET28a containing ORF of PSPPH_3486 from *P. syringae* 1448A	This study
pET28a-PSPPH_2597	pET28a containing ORF of PSPPH_2597 from *P. syringae* 1448A	This study
pET28a-PSPPH_1962	pET28a containing ORF of PSPPH_1962 from *P. syringae* 1448A	This study
pET28a-PSPPH_3027	pET28a containing ORF of PSPPH_3027 from *P. syringae* 1448A	This study
pET28a-PSPPH_2994	pET28a containing ORF of PSPPH_2994 from *P. syringae* 1448A	This study
pET28a-PSPPH_2214	pET28a containing ORF of PSPPH_2214 from *P. syringae* 1448A	This study
pET28a-PSPPH_4495	pET28a containing ORF of PSPPH_4495 from *P. syringae* 1448A	This study
pET28a-PSPPH_3202	pET28a containing ORF of PSPPH_3202 from *P. syringae* 1448A	This study
pET28a-PSPPH_2733	pET28a containing ORF of PSPPH_2733 from *P. syringae* 1448A	This study
pET28a-PSPPH_1670	pET28a containing ORF of PSPPH_1670 from P. syringae 1448A	This study
pET28a-PSPPH_3479	pET28a containing ORF of PSPPH_3479 from *P. syringae* 1448A	This study
pET28a-PSPPH_4788	pET28a containing ORF of PSPPH_4788 from *P. syringae* 1448A	This study
pET28a-PSPPH_4320	pET28a containing ORF of PSPPH_4320 from P. syringae 1448A	This study
pET28a-PSPPH_0376	pET28a containing ORF of PSPPH_0376 from *P. syringae* 1448A	This study
pET28a-PSPPH_0735	pET28a containing ORF of PSPPH_0735 from *P. syringae* 1448A	This study
pET28a-PSPPH_3268	pET28a containing ORF of PSPPH_3268 from *P. syringae* 1448A	This study
pET28a-PSPPH_4899	pET28a containing ORF of PSPPH_4899 from *P. syringae* 1448A	This study
pET28a-PSPPH_4448	pET28a containing ORF of PSPPH_4448 from *P. syringae* 1448A	This study
pET28a-PSPPH_1651	pET28a containing ORF of PSPPH_1651 from *P. syringae* 1448A	This study

pET28a-PSPPH_2984	pET28a containing ORF of PSPPH_2984 from *P. syringae* 1448A	This study
pET28a-PSPPH_3872	pET28a containing ORF of PSPPH_3872 from *P. syringae* 1448A	This study
pET28a-PSPPH_2774	pET28a containing ORF of PSPPH_2774 from *P. syringae* 1448A	This study
pET28a-PSPPH_3983	pET28a containing ORF of PSPPH_3983 from *P. syringae* 1448A	This study
pET28a-PSPPH_2905	pET28a containing ORF of PSPPH_2905 from *P. syringae* 1448A	This study
pET28a-*betI*	pET28a containing ORF of *betI* from *P. syringae* 1448A	This study
pET28a-PSPPH_0102	pET28a containing ORF of PSPPH_0102 from *P. syringae* 1448A	This study
pET28a-PSPPH_0101	pET28a containing ORF of PSPPH_0101 from *P. syringae* 1448A	This study
pET28a-PSPPH_4920	pET28a containing ORF of PSPPH_4920 from *P. syringae* 1448A	This study
pET28a-PSPPH_3182	pET28a containing ORF of PSPPH_3182 from *P. syringae* 1448A	This study
pET28a-*dksA2*	pET28a containing ORF of *dksA2* from *P. syringae* 1448A	This study
pET28a-PSPPH_5139	pET28a containing ORF of PSPPH_5139 from *P. syringae* 1448A	This study
pET28a-PSPPH_4336	pET28a containing ORF of PSPPH_4336 from *P. syringae* 1448A	This study
pET28a-*sfsA*	pET28a containing ORF of *sfsA* from *P. syringae* 1448A	This study
pET28a-*algQ*	pET28a containing ORF of *algQ* from *P. syringae* 1448A	This study
pET28a-*lon1*	pET28a containing ORF of *lon1* from *P. syringae* 1448A	Hua *et al.*, 2020

Supplementary table 1c: The following primers were used for building the HT-SELEX

1. Primers for BGI system

BGI-insert-oligo	ATGGCTACGATCCGACTTNNNNNNNNNNNNNNNNNNNNNNNNNNNNNN NNNNNNNNNAAGTCGGAGGCCAAGCGGTCTTAGGAAGACAA
BGI-system-F	/5Phos/ GAACGACATGGCTACGATCCGACTT
BGI-system-R	TGTGAGCCAAGGAGTTG
Barcode 1R	TGTGAGCCAAGGAGTTG ATCGGACCTA TTGTCTTCCTAAGACCGC
Barcode 2R	TGTGAGCCAAGGAGTTG GATTCCGTCC TTGTCTTCCTAAGACCGC
Barcode 3R	TGTGAGCCAAGGAGTTG CGGCAGTAAG TTGTCTTCCTAAGACCGC
Barcode 4R	TGTGAGCCAAGGAGTTG TCAATTAGGT TTGTCTTCCTAAGACCGC
Barcode 13R	TGTGAGCCAAGGAGTTG CGGATTGCCG TTGTCTTCCTAAGACCGC
Barcode 14R	TGTGAGCCAAGGAGTTG GAATCCTGAT TTGTCTTCCTAAGACCGC
Barcode 15R	TGTGAGCCAAGGAGTTG TCTGGAATGA TTGTCTTCCTAAGACCGC
Barcode 16R	TGTGAGCCAAGGAGTTG ATCCAGCATC TTGTCTTCCTAAGACCGC
Barcode 25R	TGTGAGCCAAGGAGTTG TTGTCCTCTA TTGTCTTCCTAAGACCGC
Barcode 26R	TGTGAGCCAAGGAGTTG ATTCGCTAGG TTGTCTTCCTAAGACCGC
Barcode 28R	TGTGAGCCAAGGAGTTG ACAGCTCAGC TTGTCTTCCTAAGACCGC
Barcode 29R	TGTGAGCCAAGGAGTTG TATCTAGGTT TTGTCTTCCTAAGACCGC
Barcode 30R	TGTGAGCCAAGGAGTTG GAGATGGCAA TTGTCTTCCTAAGACCGC
Barcode 32R	TGTGAGCCAAGGAGTTG GCCGATAGCG TTGTCTTCCTAAGACCGC
Barcode 33R	TGTGAGCCAAGGAGTTG CCATCGTTGC TTGTCTTCCTAAGACCGC
Barcode 34R	TGTGAGCCAAGGAGTTG TGAACGATTA TTGTCTTCCTAAGACCGC
Barcode 35R	TGTGAGCCAAGGAGTTG TAGAGCGAAC TTGTCTTCCTAAGACCGC
Barcode 36R	TGTGAGCCAAGGAGTTG ATGTGTGAGA TTGTCTTCCTAAGACCGC
Barcode 37R	TGTGAGCCAAGGAGTTG ATCCTAACAG TTGTCTTCCTAAGACCGC
Barcode 38R	TGTGAGCCAAGGAGTTG CGCGTCTGCG TTGTCTTCCTAAGACCGC
Barcode 39R	TGTGAGCCAAGGAGTTG GATGATCCTT TTGTCTTCCTAAGACCGC
Barcode 41R	TGTGAGCCAAGGAGTTG ATGCATCTAA TTGTCTTCCTAAGACCGC
Barcode 42R	TGTGAGCCAAGGAGTTG AGCTCTGGAC TTGTCTTCCTAAGACCGC
Barcode 43R	TGTGAGCCAAGGAGTTG CTATCACGTG TTGTCTTCCTAAGACCGC
Barcode 44R	TGTGAGCCAAGGAGTTG GGACTAGTGG TTGTCTTCCTAAGACCGC
Barcode 45R	TGTGAGCCAAGGAGTTG GCCAAGTCCA TTGTCTTCCTAAGACCGC
Barcode 46R	TGTGAGCCAAGGAGTTG CCTGTCAAGC TTGTCTTCCTAAGACCGC
Barcode 47R	TGTGAGCCAAGGAGTTG TAGAGGTCTT TTGTCTTCCTAAGACCGC
Barcode 48R	TGTGAGCCAAGGAGTTG TATGGCAACT TTGTCTTCCTAAGACCGC
Barcode 49R	TGTGAGCCAAGGAGTTG CTGCGTACAT TTGTCTTCCTAAGACCGC
Barcode 50R	TGTGAGCCAAGGAGTTG ATCTCATTAA TTGTCTTCCTAAGACCGC
Barcode 51R	TGTGAGCCAAGGAGTTG AAGTGGCGCA TTGTCTTCCTAAGACCGC
Barcode 52R	TGTGAGCCAAGGAGTTG GGCCTTAATG TTGTCTTCCTAAGACCGC
Barcode 53R	TGTGAGCCAAGGAGTTG TCTGAGGCGG TTGTCTTCCTAAGACCGC
Barcode 55R	TGTGAGCCAAGGAGTTG GATAACCGGC TTGTCTTCCTAAGACCGC
Barcode 56R	TGTGAGCCAAGGAGTTG TCAATATTCC TTGTCTTCCTAAGACCGC
Barcode 57R	TGTGAGCCAAGGAGTTG TCCGTTGAAT TTGTCTTCCTAAGACCGC
Barcode 58R	TGTGAGCCAAGGAGTTG CAGTACAGTT TTGTCTTCCTAAGACCGC
Barcode 59R	TGTGAGCCAAGGAGTTG ATTGAGGTAC TTGTCTTCCTAAGACCGC
Barcode 60R	TGTGAGCCAAGGAGTTG ATTAGAAGTC TTGTCTTCCTAAGACCGC
Barcode 61R	TGTGAGCCAAGGAGTTG CAACGCTTCA TTGTCTTCCTAAGACCGC
Barcode 62R	TGTGAGCCAAGGAGTTG GGATCGCACG TTGTCTTCCTAAGACCGC
Barcode 63R	TGTGAGCCAAGGAGTTG TGCCTTCCGA TTGTCTTCCTAAGACCGC
Barcode 64R	TGTGAGCCAAGGAGTTG GCGACATCGG TTGTCTTCCTAAGACCGC
Barcode 65R	TGTGAGCCAAGGAGTTG CATTCTAAGT TTGTCTTCCTAAGACCGC
Barcode 66R	TGTGAGCCAAGGAGTTG CAGGCTTGGA TTGTCTTCCTAAGACCGC
Barcode 67R	TGTGAGCCAAGGAGTTG ATCATCGTCT TTGTCTTCCTAAGACCGC
Barcode 68R	TGTGAGCCAAGGAGTTG GTCTTGTGAG TTGTCTTCCTAAGACCGC
Barcode 69R	TGTGAGCCAAGGAGTTG AGTAGGAACG TTGTCTTCCTAAGACCGC
Barcode 70R	TGTGAGCCAAGGAGTTG TCACAACCAC TTGTCTTCCTAAGACCGC

Barcode 71R	TGTGAGCCAAGGAGTTG GCAGGCCTTC TTGTCTTCCTAAGACCGC
Barcode 72R	TGTGAGCCAAGGAGTTG TGGCAAGCTA TTGTCTTCCTAAGACCGC
Barcode 73R	TGTGAGCCAAGGAGTTG GAGCATTGTC TTGTCTTCCTAAGACCGC
Barcode 74R	TGTGAGCCAAGGAGTTG TGTGATTAGC TTGTCTTCCTAAGACCGC
Barcode 75R	TGTGAGCCAAGGAGTTG CCTATGGACT TTGTCTTCCTAAGACCGC
Barcode 76R	TGTGAGCCAAGGAGTTG TAGGCGATAG TTGTCTTCCTAAGACCGC
Barcode 77R	TGTGAGCCAAGGAGTTG AGACCACGAT TTGTCTTCCTAAGACCGC
Barcode 78R	TGTGAGCCAAGGAGTTG GTATTAGCCA TTGTCTTCCTAAGACCGC
Barcode 79R	TGTGAGCCAAGGAGTTG CTCTGCACTG TTGTCTTCCTAAGACCGC
Barcode 80R	TGTGAGCCAAGGAGTTG ACCAGCCTGA TTGTCTTCCTAAGACCGC
Barcode 81R	TGTGAGCCAAGGAGTTG GCGTGAGTAT TTGTCTTCCTAAGACCGC
Barcode 82R	TGTGAGCCAAGGAGTTG CGCGGAGCAT TTGTCTTCCTAAGACCGC
Barcode 83R	TGTGAGCCAAGGAGTTG CAAGTTCACA TTGTCTTCCTAAGACCGC
Barcode 84R	TGTGAGCCAAGGAGTTG AGCACCTCTC TTGTCTTCCTAAGACCGC
Barcode 85R	TGTGAGCCAAGGAGTTG TTACAGTGCA TTGTCTTCCTAAGACCGC
Barcode 86R	TGTGAGCCAAGGAGTTG TTGCCTAGGC TTGTCTTCCTAAGACCGC
Barcode 87R	TGTGAGCCAAGGAGTTG GCTATGATGG TTGTCTTCCTAAGACCGC
Barcode 88R	TGTGAGCCAAGGAGTTG AATTACCATG TTGTCTTCCTAAGACCGC
Barcode 89R	TGTGAGCCAAGGAGTTG AGACATGGTG TTGTCTTCCTAAGACCGC
Barcode 90R	TGTGAGCCAAGGAGTTG CCAGACATAT TTGTCTTCCTAAGACCGC
Barcode 91R	TGTGAGCCAAGGAGTTG ACGCTTCCTT TTGTCTTCCTAAGACCGC
Barcode 92R	TGTGAGCCAAGGAGTTG GACGTCTTGA TTGTCTTCCTAAGACCGC
Barcode 93R	TGTGAGCCAAGGAGTTG TACTGAGCGG TTGTCTTCCTAAGACCGC
Barcode 94R	TGTGAGCCAAGGAGTTG TGTACACACC TTGTCTTCCTAAGACCGC
Barcode 95R	TGTGAGCCAAGGAGTTG CTTACGTGAA TTGTCTTCCTAAGACCGC
Barcode 96R	TGTGAGCCAAGGAGTTG GTGTGGAACC TTGTCTTCCTAAGACCGC
Barcode 97R	TGTGAGCCAAGGAGTTG AAGAATACCT TTGTCTTCCTAAGACCGC
Barcode 98R	TGTGAGCCAAGGAGTTG GTTGCATTCG TTGTCTTCCTAAGACCGC
Barcode 99R	TGTGAGCCAAGGAGTTG CGCCGTTGAA TTGTCTTCCTAAGACCGC
Barcode 100R	TGTGAGCCAAGGAGTTG TTCCGCCGAG TTGTCTTCCTAAGACCGC
Barcode 101R	TGTGAGCCAAGGAGTTG CCATTACCGT TTGTCTTCCTAAGACCGC
Barcode 102R	TGTGAGCCAAGGAGTTG ACGTCGGATC TTGTCTTCCTAAGACCGC
Barcode 103R	TGTGAGCCAAGGAGTTG TGTATCGTGA TTGTCTTCCTAAGACCGC
Barcode 104R	TGTGAGCCAAGGAGTTG GAAGAGAATC TTGTCTTCCTAAGACCGC
Barcode 114R	TGTGAGCCAAGGAGTTG CTGACTCTGG TTGTCTTCCTAAGACCGC
Barcode 115R	TGTGAGCCAAGGAGTTG ACTGCCTGTT TTGTCTTCCTAAGACCGC
Barcode 116R	TGTGAGCCAAGGAGTTG GTCATGGAGC TTGTCTTCCTAAGACCGC
Barcode 117R	TGTGAGCCAAGGAGTTG GGATAGACAT TTGTCTTCCTAAGACCGC
Barcode 121R	TGTGAGCCAAGGAGTTG TTGATCAAGG TTGTCTTCCTAAGACCGC
Barcode 122R	TGTGAGCCAAGGAGTTG TGCCACTTCC TTGTCTTCCTAAGACCGC
Barcode 123R	TGTGAGCCAAGGAGTTG GTAGAATGTT TTGTCTTCCTAAGACCGC
Barcode 124R	TGTGAGCCAAGGAGTTG GACTCGCGTC TTGTCTTCCTAAGACCGC
Barcode 125R	TGTGAGCCAAGGAGTTG AGTGTTATAG TTGTCTTCCTAAGACCGC
Barcode 126R	TGTGAGCCAAGGAGTTG ACACGAGACT TTGTCTTCCTAAGACCGC
Barcode 127R	TGTGAGCCAAGGAGTTG CATAGGCCGA TTGTCTTCCTAAGACCGC
Barcode 128R	TGTGAGCCAAGGAGTTG CCGTCTGCAA TTGTCTTCCTAAGACCGC
**2. Primers for Illumina system**	
SELEX-F	AATGATACGGCGACCACCGAGATCTACAC
SELEX-R	CAAGCAGAAGACGGCATACGAGAT
N50N70	TCCCTACACGAC GCTCTTCCGATCT<br>NNNNNNNNNNNNNNNNNNNNNNNNNNNNNNNNNNNNNNNN<br>GATCGGAAGAGC ACACGTCTGAACTCCAGTCAC
N501	AATGATACGGCGACCACCGAGATCTACAC TAGATCGC<br>ACACTCTTTCCCTACACGAC GCTCTTCCGATCT
N502	AATGATACGGCGACCACCGAGATCTACAC CTCTCTAT<br>ACACTCTTTCCCTACACGAC GCTCTTCCGATCT

N503	AATGATACGGCGACCACCGAGATCTACAC TATCCTCT ACACTCTTTCCCTACACGAC GCTCTTCCGATCT
N504	AATGATACGGCGACCACCGAGATCTACAC AGAGTAGA ACACTCTTTCCCTACACGAC GCTCTTCCGATCT
N505	AATGATACGGCGACCACCGAGATCTACAC GTAAGGAG ACACTCTTTCCCTACACGAC GCTCTTCCGATCT
N506	AATGATACGGCGACCACCGAGATCTACAC ACTGCATA ACACTCTTTCCCTACACGAC GCTCTTCCGATCT
N507	AATGATACGGCGACCACCGAGATCTACAC AAGGAGTA ACACTCTTTCCCTACACGAC GCTCTTCCGATCT
N508	AATGATACGGCGACCACCGAGATCTACAC CTAAGCCT ACACTCTTTCCCTACACGAC GCTCTTCCGATCT
N701	CAAGCAGAAGACGGCATACGAGAT TCGCCTTA GTGACTGGAGTTCAGACGTGT
N702	CAAGCAGAAGACGGCATACGAGAT CTAGTACG GTGACTGGAGTTCAGACGTGT
N703	CAAGCAGAAGACGGCATACGAGAT TTCTGCCT GTGACTGGAGTTCAGACGTGT
N704	CAAGCAGAAGACGGCATACGAGAT GCTCAGGA GTGACTGGAGTTCAGACGTGT
N705	CAAGCAGAAGACGGCATACGAGAT AGGAGTCC GTGACTGGAGTTCAGACGTGT
N706	CAAGCAGAAGACGGCATACGAGAT CATGCCTA GTGACTGGAGTTCAGACGTGT
N707	CAAGCAGAAGACGGCATACGAGAT GTAGAGAG GTGACTGGAGTTCAGACGTGT
N708	CAAGCAGAAGACGGCATACGAGAT CCTCTCTG GTGACTGGAGTTCAGACGTGT
N709	CAAGCAGAAGACGGCATACGAGAT AGCGTAGC GTGACTGGAGTTCAGACGTGT
N710	CAAGCAGAAGACGGCATACGAGAT CAGCCTCG GTGACTGGAGTTCAGACGTGT
N711	CAAGCAGAAGACGGCATACGAGAT TGCCTCTT GTGACTGGAGTTCAGACGTGT
N712	CAAGCAGAAGACGGCATACGAGAT TCCTCTAC GTGACTGGAGTTCAGACGTGT
SA501	AATGATACGGCGACCACCGAGATCTACAC ATCGTACG ACACTCTTTCCCTACACGAC GCTCTTCCGATCT
SA502	AATGATACGGCGACCACCGAGATCTACAC ACTATCTG ACACTCTTTCCCTACACGAC GCTCTTCCGATCT
SA503	AATGATACGGCGACCACCGAGATCTACAC TAGCGAGT ACACTCTTTCCCTACACGAC GCTCTTCCGATCT
SA504	AATGATACGGCGACCACCGAGATCTACAC CTGCGTGT ACACTCTTTCCCTACACGAC GCTCTTCCGATCT
SA505	AATGATACGGCGACCACCGAGATCTACAC TCATCGAG ACACTCTTTCCCTACACGAC GCTCTTCCGATCT
SA506	AATGATACGGCGACCACCGAGATCTACAC CGTGAGTG ACACTCTTTCCCTACACGAC GCTCTTCCGATCT
SA507	AATGATACGGCGACCACCGAGATCTACAC GGATATCT ACACTCTTTCCCTACACGAC GCTCTTCCGATCT
SA508	AATGATACGGCGACCACCGAGATCTACAC GACACCGT ACACTCTTTCCCTACACGAC GCTCTTCCGATCT
SA701	CAAGCAGAAGACGGCATACGAGAT CGAGAGTT GTGACTGGAGTTCAGACGTGT
SA702	CAAGCAGAAGACGGCATACGAGAT GACATAGT GTGACTGGAGTTCAGACGTGT

SA703	CAAGCAGAAGACGGCATACGAGAT ACGCTACT GTGACTGGAGTTCAGACGTGT
SA704	CAAGCAGAAGACGGCATACGAGAT ACTCACAG GTGACTGGAGTTCAGACGTGT
SA705	CAAGCAGAAGACGGCATACGAGAT TGAGTAGG GTGACTGGAGTTCAGACGTGT
SA706	CAAGCAGAAGACGGCATACGAGAT CTGCGTAG GTGACTGGAGTTCAGACGTGT
SA707	CAAGCAGAAGACGGCATACGAGAT TAGTCTCC GTGACTGGAGTTCAGACGTGT
SA708	CAAGCAGAAGACGGCATACGAGAT CGAGCGAC GTGACTGGAGTTCAGACGTGT
SA709	CAAGCAGAAGACGGCATACGAGAT ACTACGAC GTGACTGGAGTTCAGACGTGT
SA710	CAAGCAGAAGACGGCATACGAGAT GTCTGCTA GTGACTGGAGTTCAGACGTGT
SA711	CAAGCAGAAGACGGCATACGAGAT GTCTATGA GTGACTGGAGTTCAGACGTGT
SA712	CAAGCAGAAGACGGCATACGAGAT TATAGCGA GTGACTGGAGTTCAGACGTGT
