# Supplementary material for: A compendium of DNA-binding specificities of transcription factors in Pseudomonas syringae
Source: Nat Commun. 2020 Oct 2;11:4947. doi: 10.1038/s41467-020-18744-7 (PMC7532196; doi:10.1038/s41467-020-18744-7)
Supplement: Supplementary file 3 — Description of Additional Supplementary Files [file 41467_2020_18744_MOESM3_ESM.pdf]

Description of Additional Supplementary Files

File name: Supplementary Data 1.

Description: TF similarity network with motif logos, related to Fig. 1. The 69 distinct modules of TFs of the obtained PWMs. Diamonds indicate TFs, circles indicate individual PWMs. The dashed lines show the motif of the TF. The TFs without names are named with their locus tag omitting "PSPPH_".

File name: Supplementary Data 2.

Description: List of putative genomic binding sites for individual TFs, 100 TFs in total, related to Fig. 3.

File name: Supplementary Data 3.

Description: Transcriptional regulatory networks of non-T3SS pathways, related to Fig. 4. Networks illustrate the regulatory relationship between TFs and their target genes in non-T3SS virulent pathways, including c-di-GMP (a), Flagella-Mediated Motility (b), Surface Attachment (c), Siderophore (d), Phytotoxin (e) and ROS (f). Circles indicate TF proteins, and rectangles indicate target genes. Red arrows show the binding sites located in the putative promoters of the target genes, while blue arrows show the binding sites located in the target gene bodies. The TFs without names are named with their locus tag omitting "PSPPH_".

File name: Supplementary Data 4.

Description: This table contains background subtracted PWMs for all factors analyzed.
