# Supplementary material for: A compendium of DNA-binding specificities of transcription factors in Pseudomonas syringae
Source: Nat Commun. 2020 Oct 2;11:4947. doi: 10.1038/s41467-020-18744-7 (PMC7532196; doi:10.1038/s41467-020-18744-7)
Supplement: Supplementary file 4 — Supplementary Data 1 [file 41467_2020_18744_MOESM4_ESM.pdf]

Supplementary Data 1

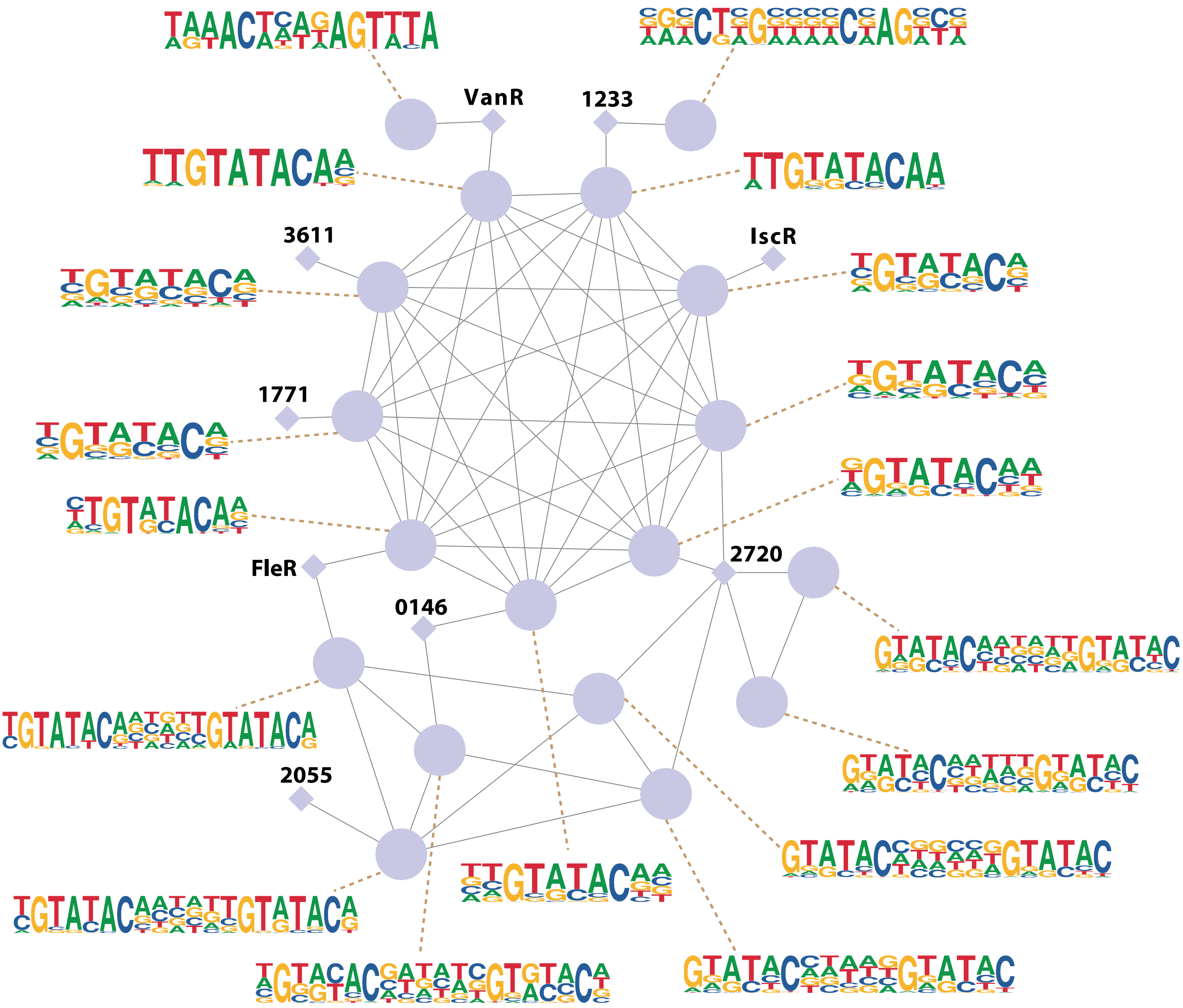

Module 1

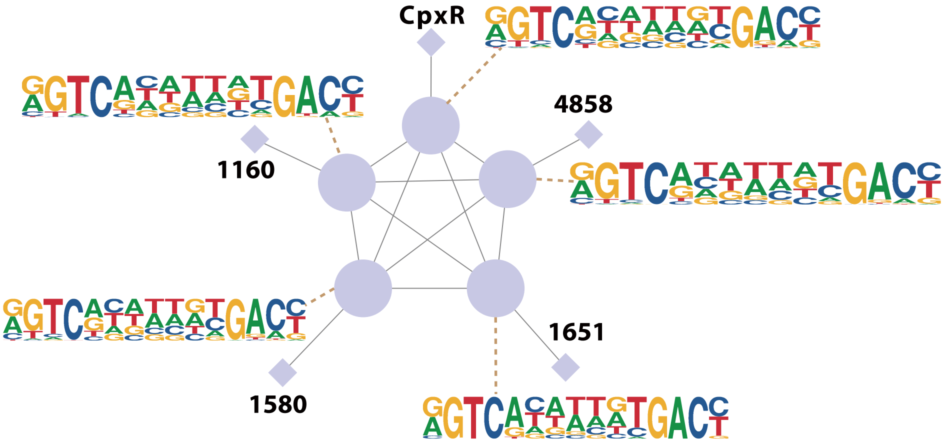

Module 2

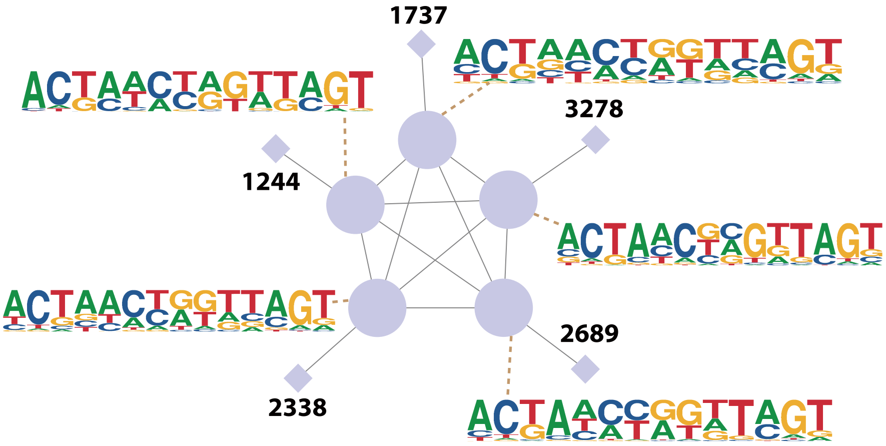

Module 3

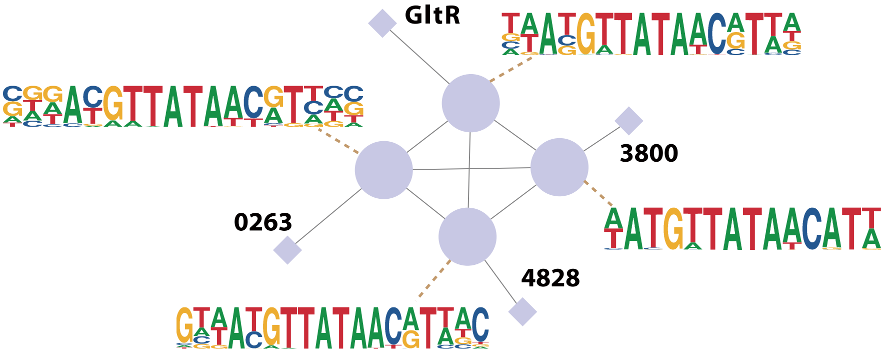

Module 4

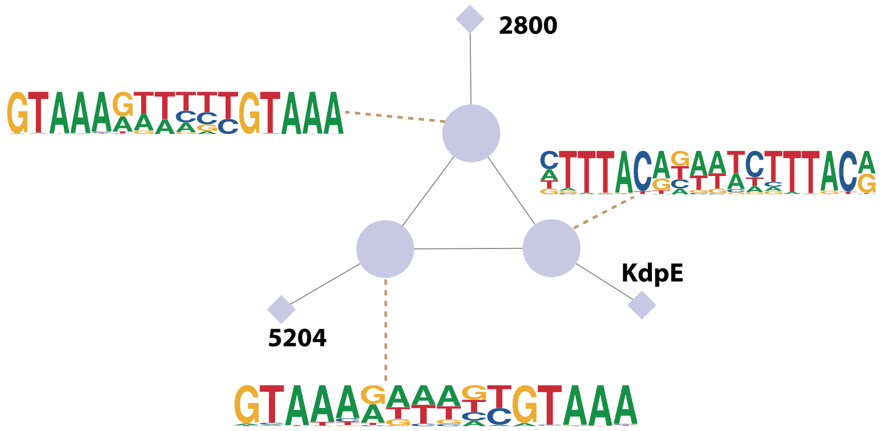

Module 5

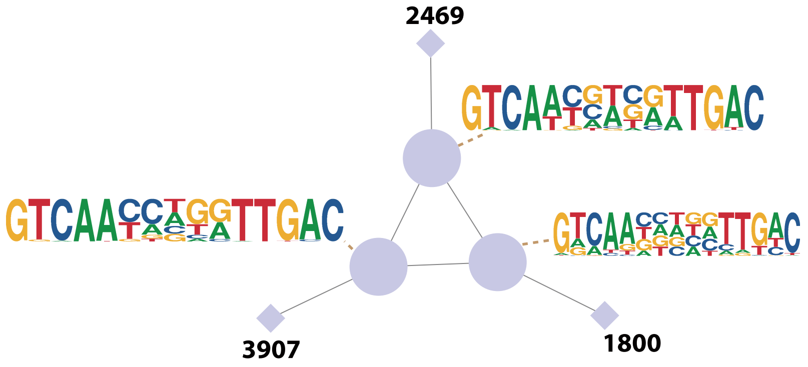

Module 6

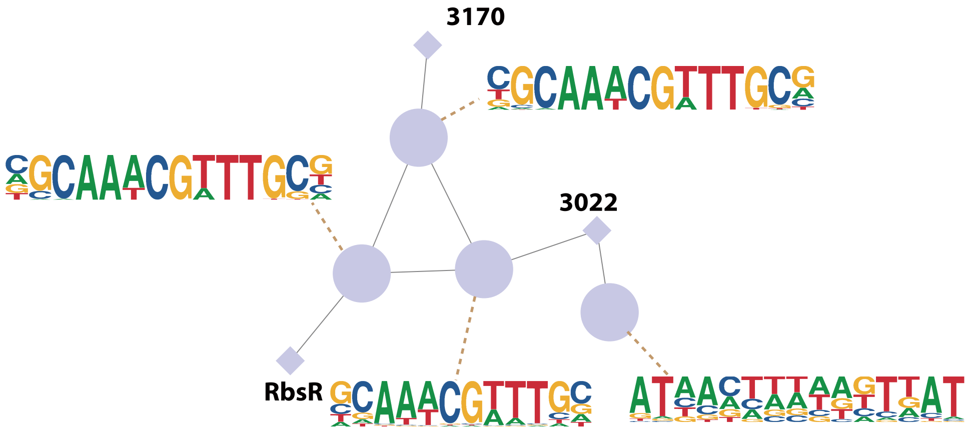

Module 7

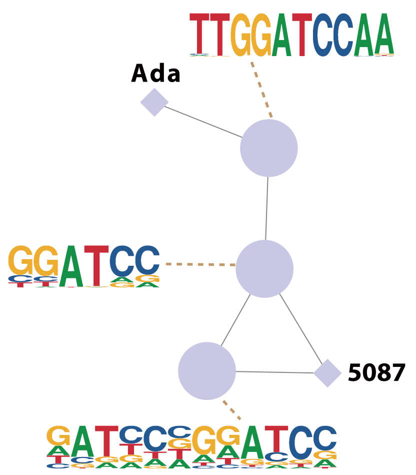

Module 8

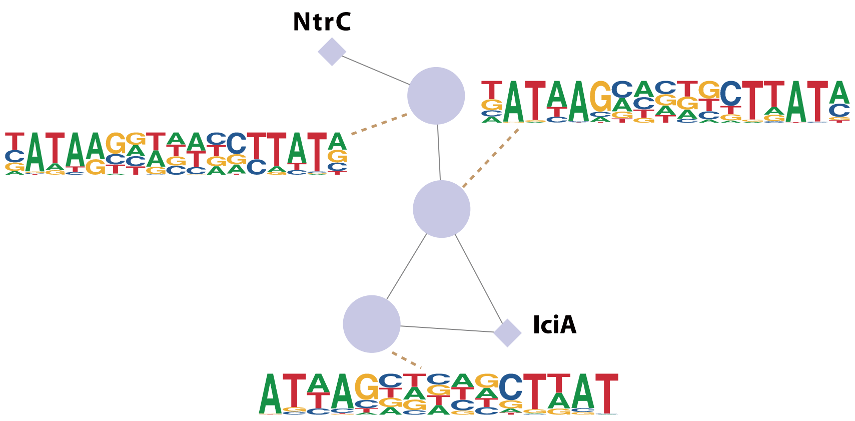

Module 9

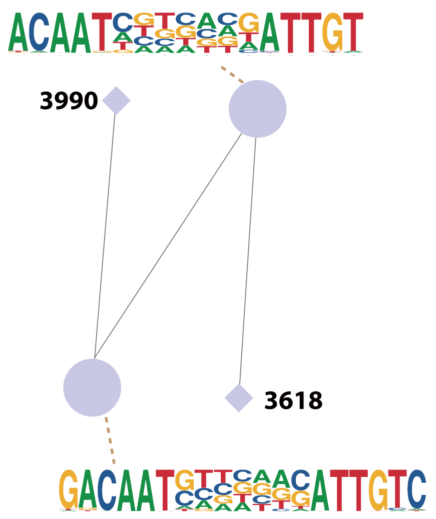

Module 10

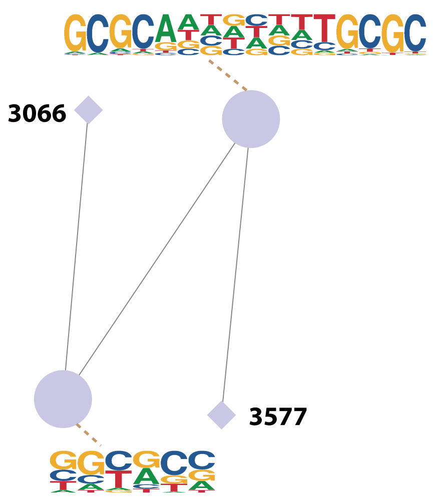

Module 11

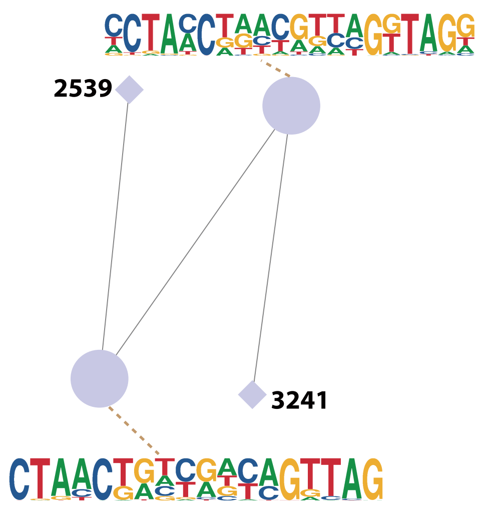

Module 12

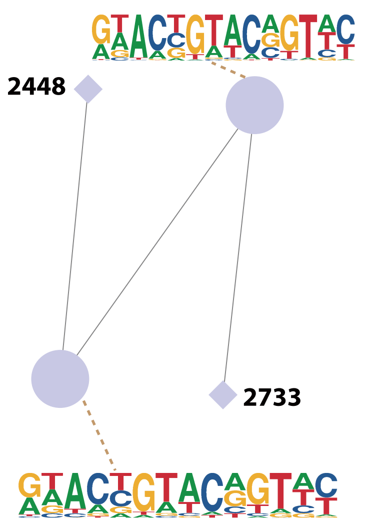

Module 13

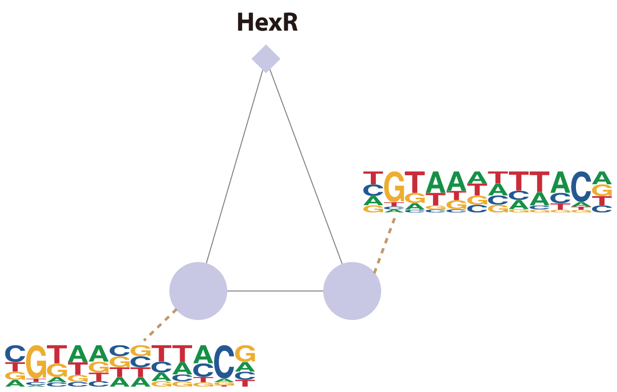

Module 14

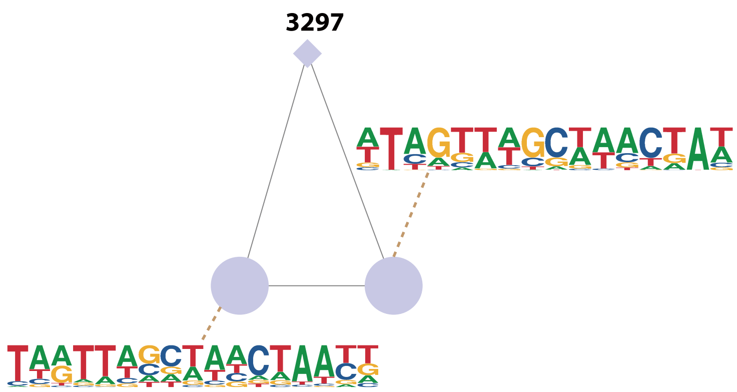

Module 15

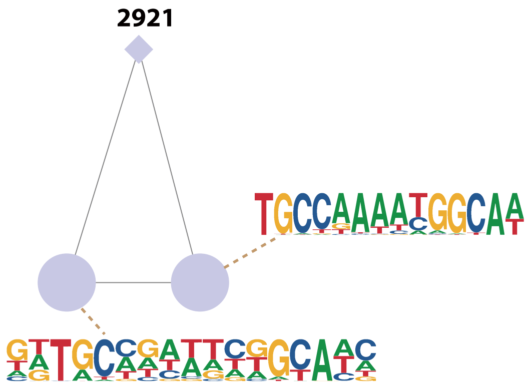

Module 16

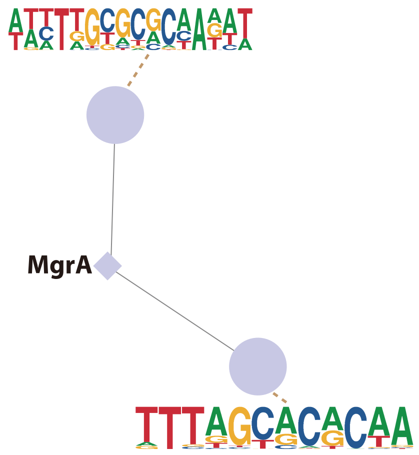

Module 17

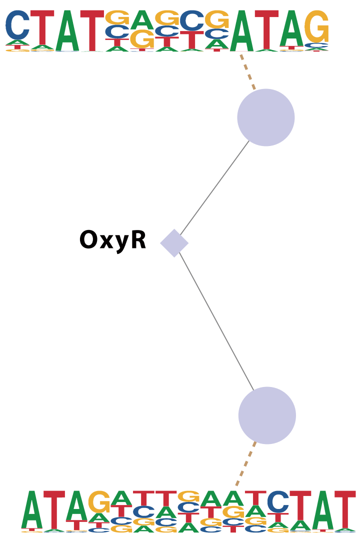

Module 18

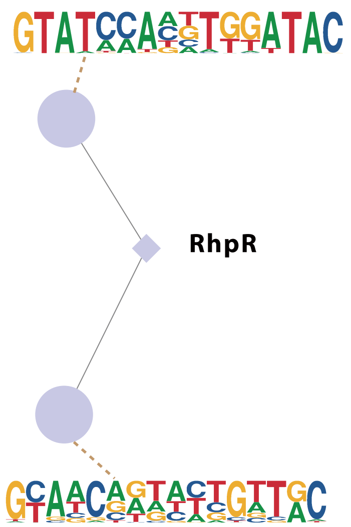

Module 19

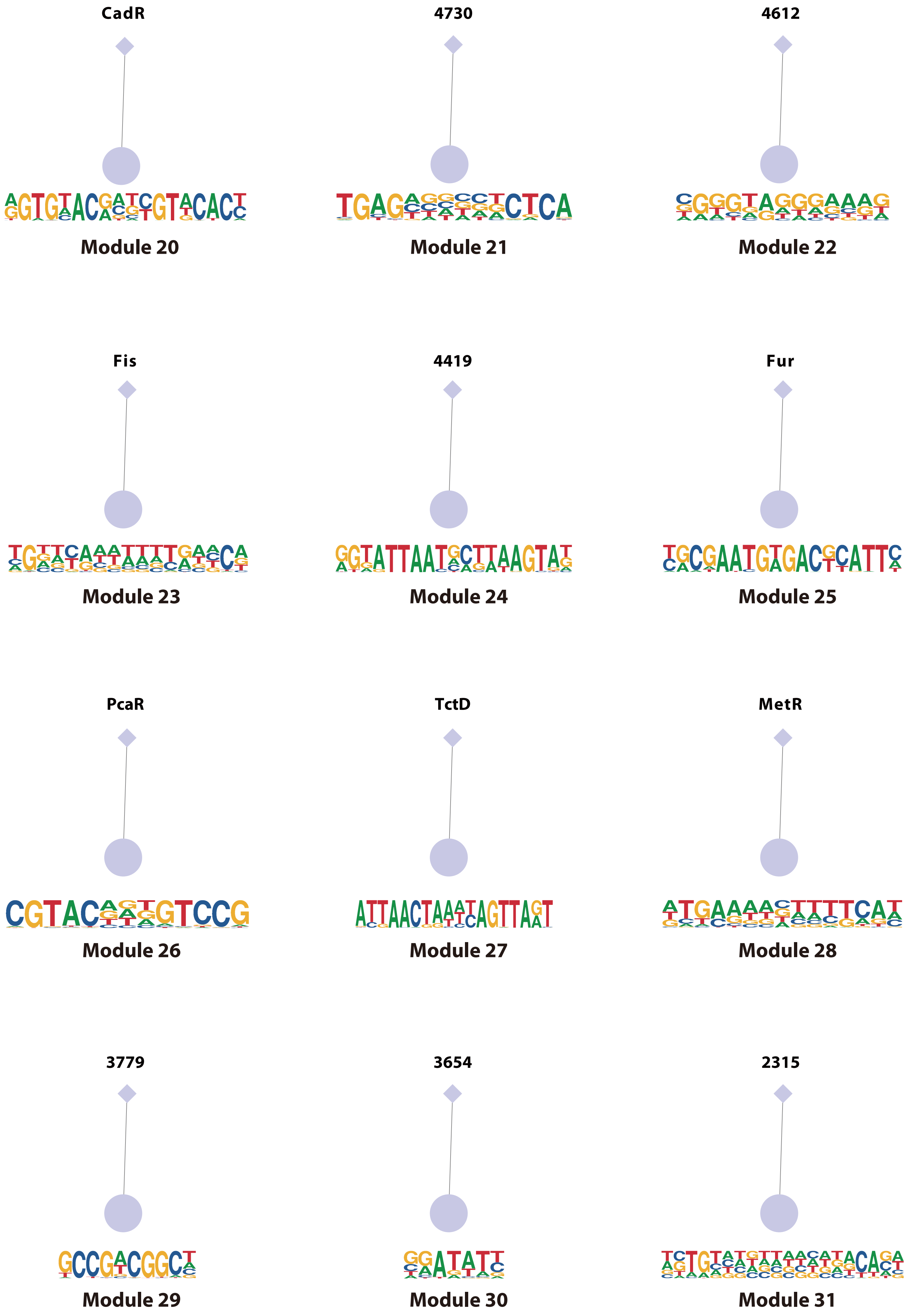
CadR
Module 20
4730
Module 21
4612
Module 22
Fis
Module 23
4419
Module 24
Fur
Module 25
PcaR
Module 26
TctD
Module 27
MetR
Module 28
3779
Module 29
3654
Module 30
2315
Module 31

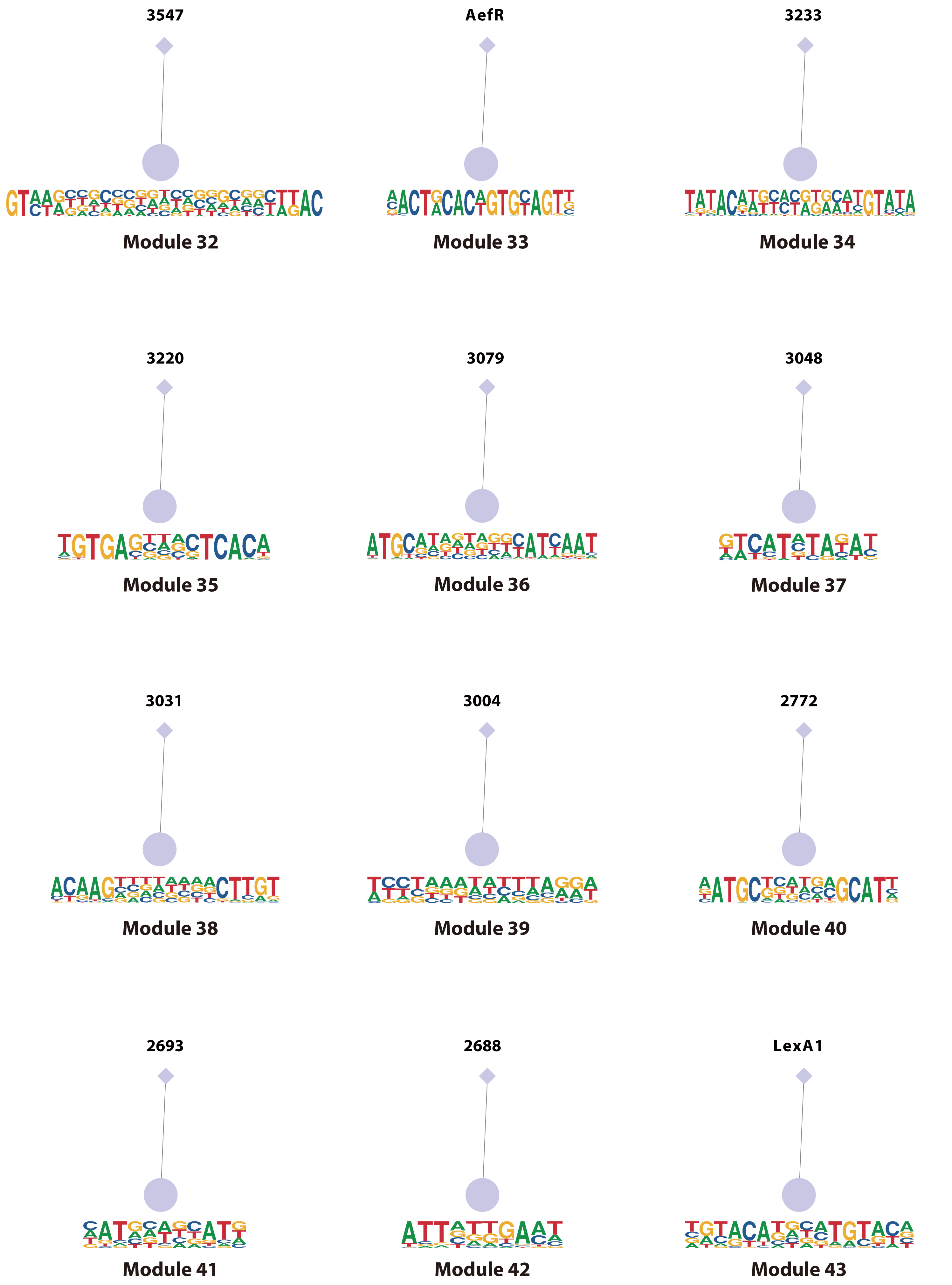
3547
AefR
3233
Module 32
Module 33
Module 34
3220
3079
3048
Module 35
Module 36
Module 37
3031
3004
2772
Module 38
Module 39
Module 40
2693
2688
LexA1
Module 41
Module 42
Module 43

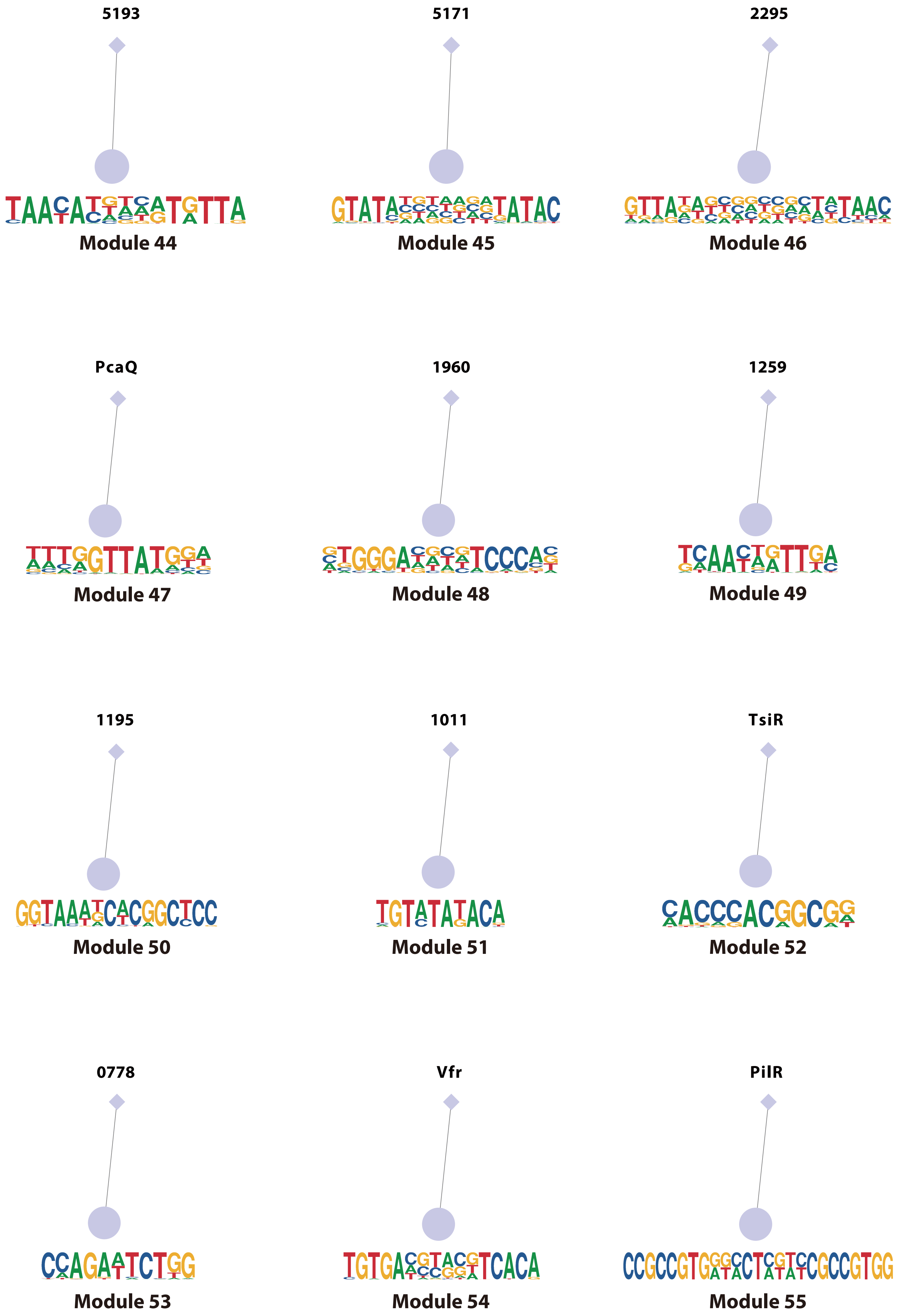
5193
Module 44
5171
Module 45
2295
Module 46
PcaQ
Module 47
1960
Module 48
1259
Module 49
1195
Module 50
1011
Module 51
TsiR
Module 52
0778
Module 53
Vfr
Module 54
PilR
Module 55

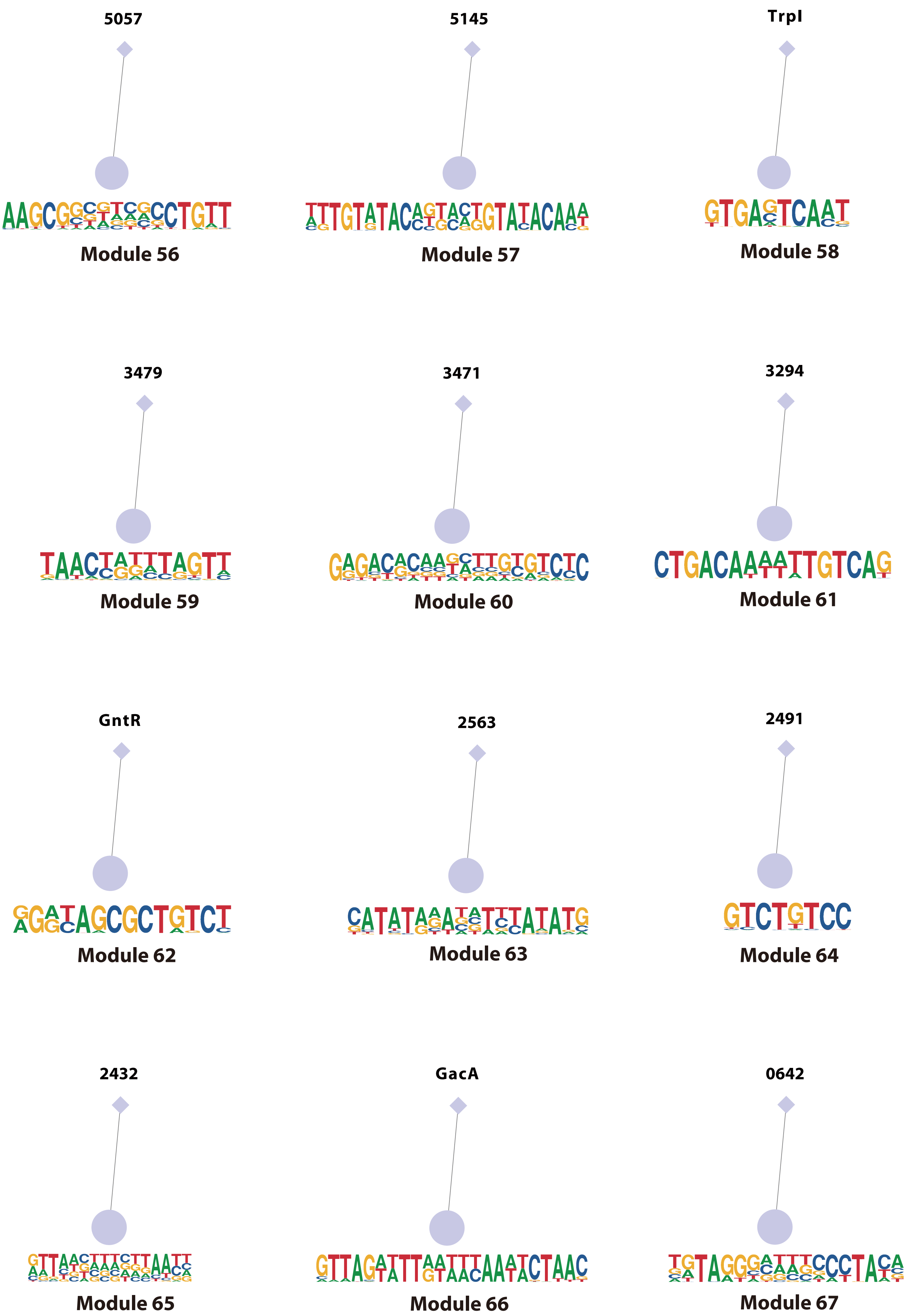
5057
Module 56
5145
Module 57
TrpI
Module 58
3479
Module 59
3471
Module 60
3294
Module 61
GntR
Module 62
2563
Module 63
2491
Module 64
2432
Module 65
GacA
Module 66
0642
Module 67

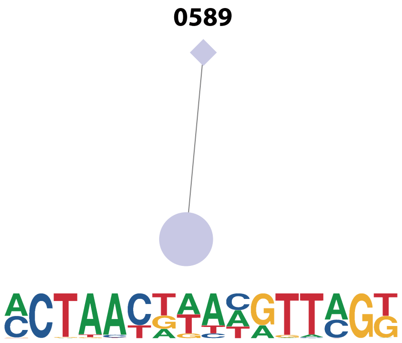

module 68

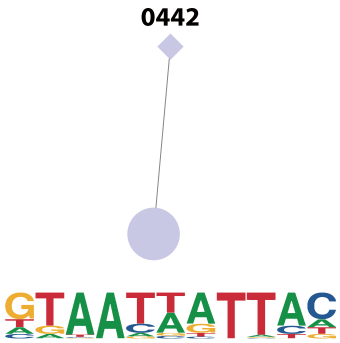

module 69
