# Supplementary material for: A compendium of DNA-binding specificities of transcription factors in Pseudomonas syringae
Source: Nat Commun. 2020 Oct 2;11:4947. doi: 10.1038/s41467-020-18744-7 (PMC7532196; doi:10.1038/s41467-020-18744-7)

Supplementary Data 3

a. Transcriptional Regulatory Network of c-di-GMP

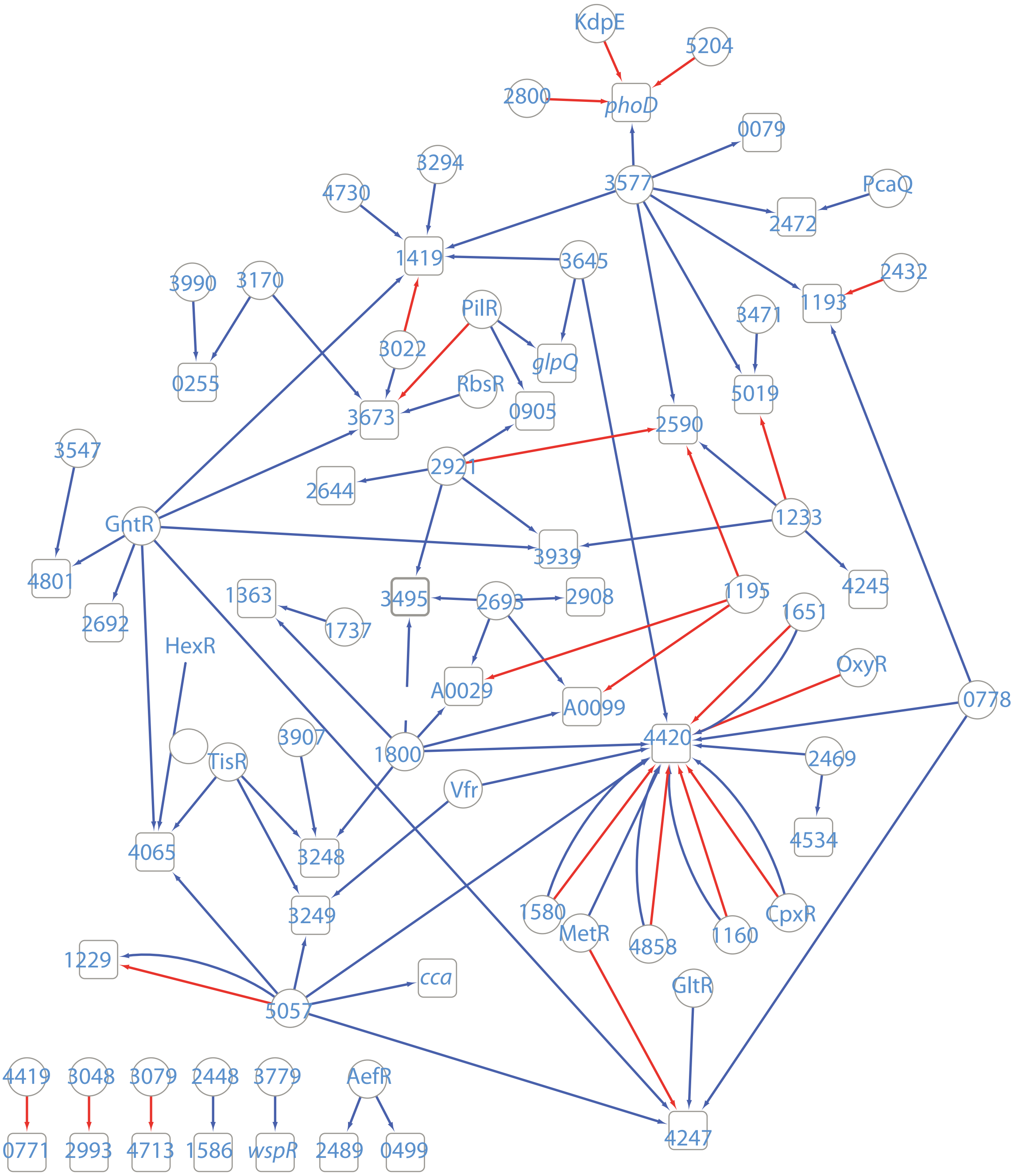

b. Transcriptional Regulatory Network of Flagell a-Mediated Motility

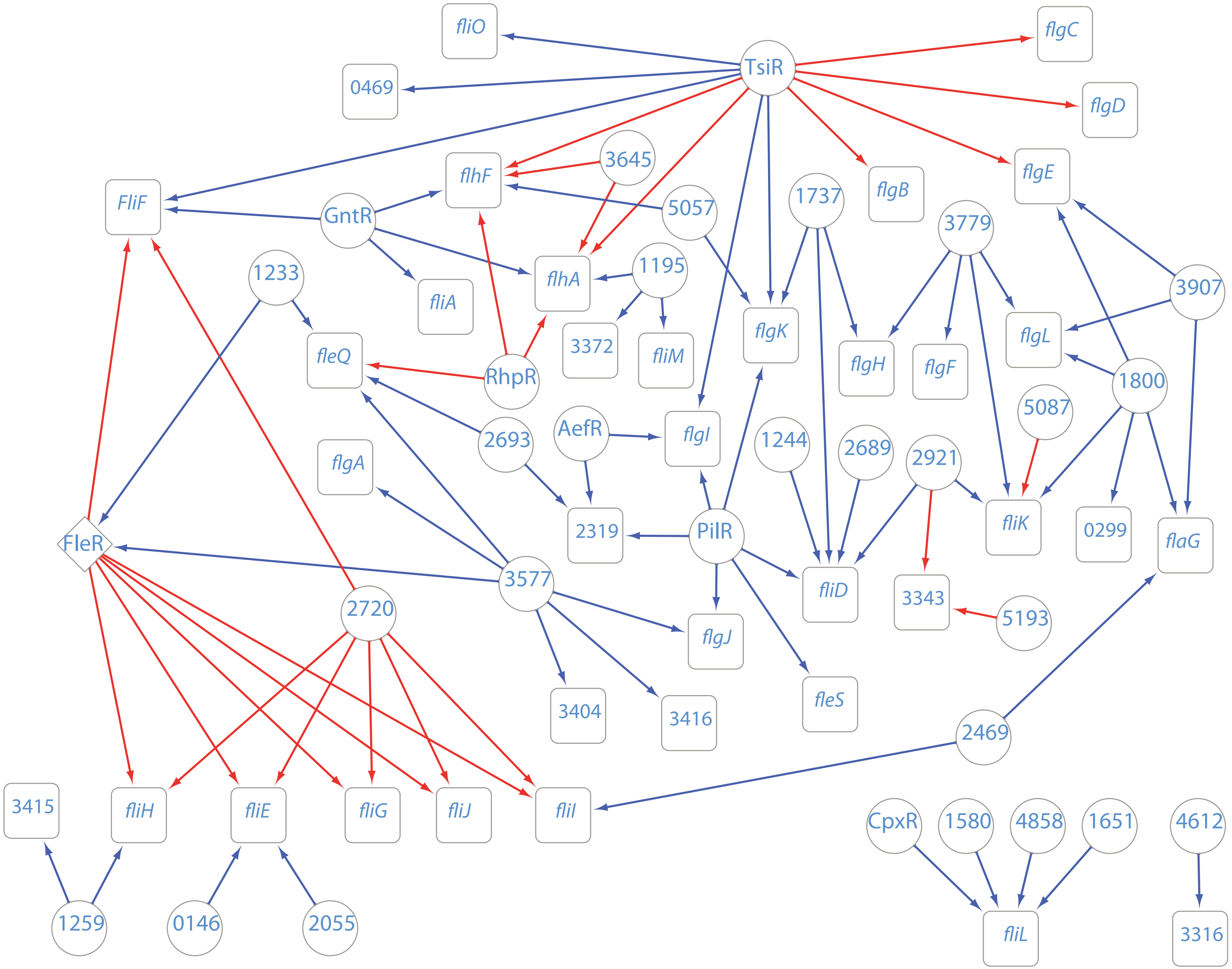

c. Transcriptional Regulatory Network of Surface Attachment

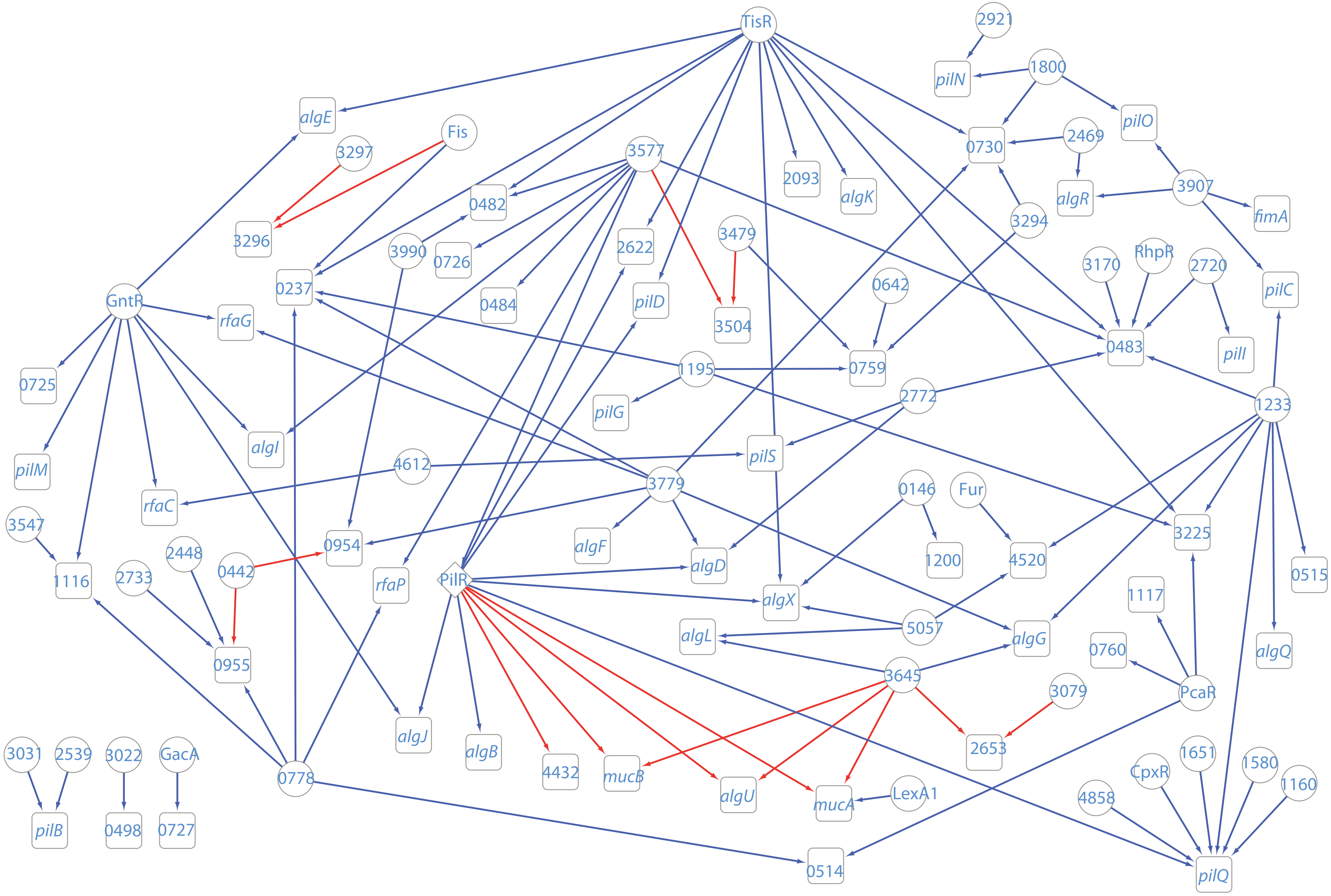

d. Transcriptional Regulatory Network of Siderophore

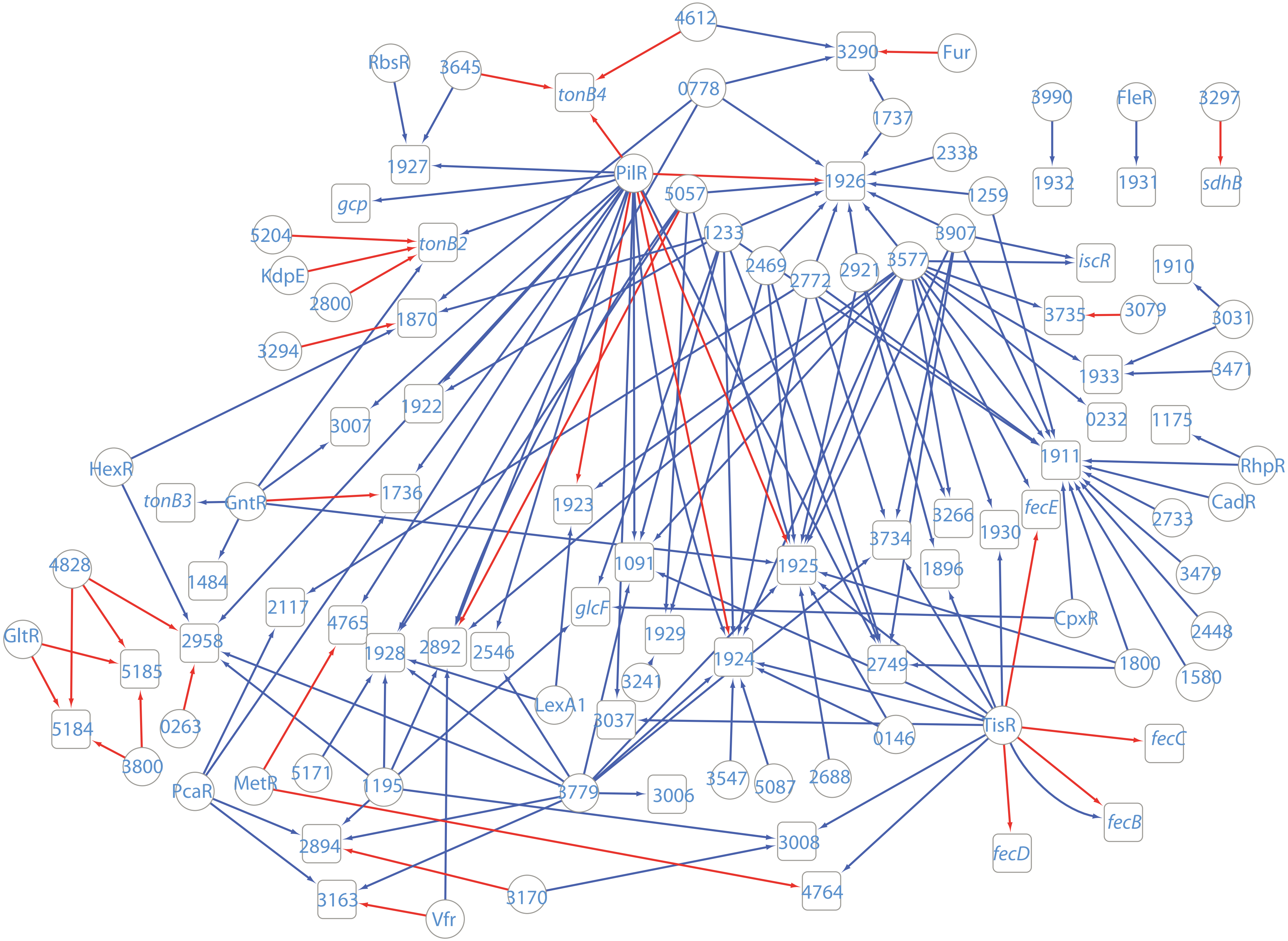

e. Transcriptional Regulatory Network of Phytotoxin

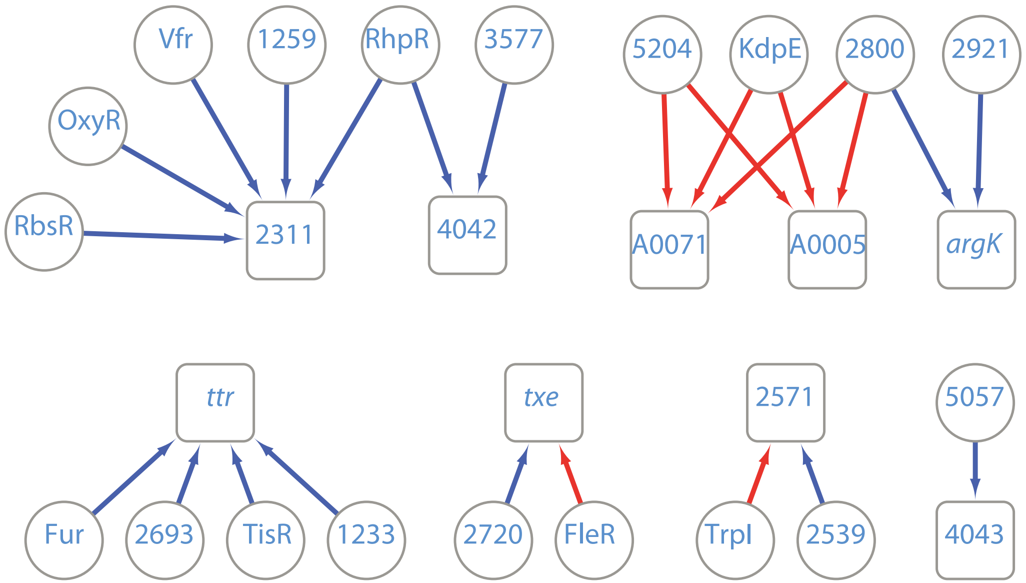

f. Transcriptional Regulatory Network of ROS

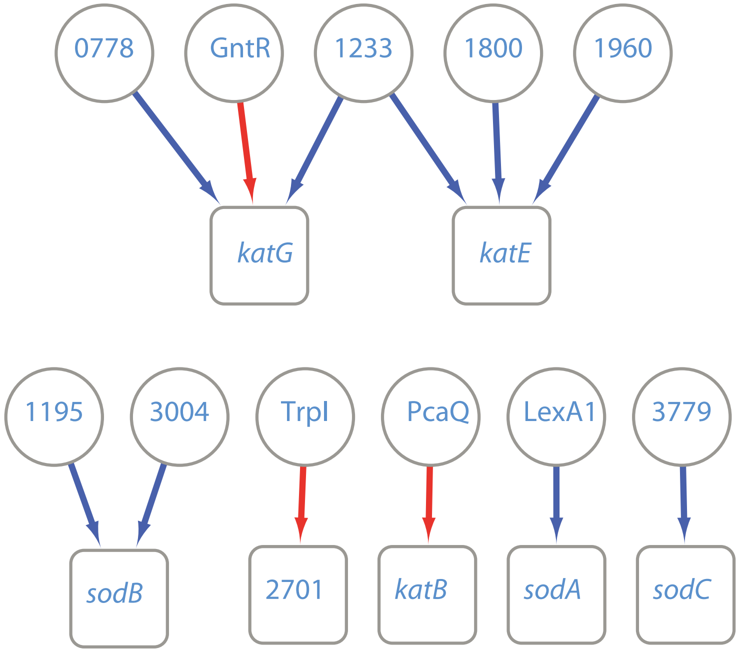

Supplement: Supplementary file 6 — Supplementary Data 3 [file 41467_2020_18744_MOESM6_ESM.pdf]
