# Supplementary material for: A compendium of DNA-binding specificities of transcription factors in Pseudomonas syringae
Source: Nat Commun. 2020 Oct 2;11:4947. doi: 10.1038/s41467-020-18744-7 (PMC7532196; doi:10.1038/s41467-020-18744-7)
Supplement: Supplementary file 8 — Reporting Summary [file 41467_2020_18744_MOESM8_ESM.pdf]

nature research

Corresponding author(s): Xin Deng, Jian Yan

Last updated by author(s): Aug 26, 2020

Reporting Summary

Nature Research wishes to improve the reproducibility of the work that we publish. This form provides structure for consistency and transparency in reporting. For further information on Nature Research policies, see our Editorial Policies and the Editorial Policy Checklist.

Statistics

For all statistical analyses, confirm that the following items are present in the figure legend, table legend, main text, or Methods section.

n/a	Confirmed	
☐	☒	The exact sample size (*n*) for each experimental group/condition, given as a discrete number and unit of measurement
☐	☒	A statement on whether measurements were taken from distinct samples or whether the same sample was measured repeatedly
☐	☒	The statistical test(s) used AND whether they are one- or two-sided<br>*Only common tests should be described solely by name; describe more complex techniques in the Methods section.*
☒	☐	A description of all covariates tested
☐	☒	A description of any assumptions or corrections, such as tests of normality and adjustment for multiple comparisons
☐	☒	A full description of the statistical parameters including central tendency (e.g. means) or other basic estimates (e.g. regression coefficient) AND variation (e.g. standard deviation) or associated estimates of uncertainty (e.g. confidence intervals)
☐	☒	For null hypothesis testing, the test statistic (e.g. *F*, *t*, *r*) with confidence intervals, effect sizes, degrees of freedom and *P* value noted<br>*Give P values as exact values whenever suitable.*
☒	☐	For Bayesian analysis, information on the choice of priors and Markov chain Monte Carlo settings
☒	☐	For hierarchical and complex designs, identification of the appropriate level for tests and full reporting of outcomes
☒	☐	Estimates of effect sizes (e.g. Cohen's *d*, Pearson's *r*), indicating how they were calculated

Our web collection on statistics for biologists contains articles on many of the points above.

Software and code

Policy information about availability of computer code

Data collection

No software was used.

Data analysis

1. ChIP-seq analysis
The raw ChIP-seq data were downloaded from GEO (GSE122629) and were previously published by our lab. The data were mapped to P. syringae 1448A genome by using bowtie (v1.2.2). Only the uniquely mapped reads were kept for the subsequent analyses. Binding peaks were identified using MACS software (v2.1.2). Peaks were annotated by using bedtools (v2.25.0)83. The peaks and reads distribution were visualized by using IGV software (v2.4.14).

2. RNA-seq analysis
The raw RNA-Seq data were downloaded from GEO (GSE122629). These data were previously published by our lab. RNA-seq data were mapped to the P. syringae 1448A genome by using HSAT (v 2.1.0). Only the uniquely mapped reads were kept for the subsequent analyses. Gene expression was quantified using GFOLD (v1.1.4). Reads distribution was visualized by using IGV software (v2.4.14).

3. HT-SELEX data analysis
Raw sequencing data were binned according to barcodes for each sample. Sequences from the 40-nt random region without bases annotated as N were used for further analyses. PWM models were generated using initial seeds identified using Autoseed that were refined by expert analysis as described in Jolma et al. Exact seeds, cycles, and multinomial model were indicated in Supplementary Table 2. All motif seqlogos were generated using the R package ggseqlogo.

4. Network analysis of similarity between the PWMs
We calculated the similarities of all pairs of 118 PWMs using SSTAT (parameters: 50% GC-content, pseudocount regularization, type I threshold 0.01) as described in Jolma et al. We generated a network containing two types of nodes, one type representing TF binding profiles, and another type representing TF proteins. TF protein nodes were connected to their binding models, and the binding models were further

connected to each other if their SSTAT similarity score (asymptotic covariance) was greater than 1.5 x 10-5 as described in Jolma et al. Finally, the network was visualized using Cytoscape software v3.7.2.

5. Analysis of the transcriptional regulatory networks
We first scanned the P. syringae reference genome with 118 PWMs using FIMO, and then used bedtools (v2.25.0) to annotate all putative binding sites. We generated transcriptional regulatory networks for 7 important systems of P. syringae that contained two types of nodes, one type representing TF proteins, and another type representing targets. TF protein nodes were connected to their targets. All networks were visualized using Cytoscape software v3.7.2. We used the hypergeometric distribution to calculate the statistical significance (p value) and thus analyze the master regulators for each specific pathway and visualize it using python matplotlib package: the probability of having the number of genes targeted by a TF (n) belonging to a specific pathways (k) could be described with the hypergeometric distribution, from the genes in that pathway (K) out of the total number of genes in the P. syringae genome (N). Our null hypothesis is that the target genes of a TF are not over-represented in a specific pathway. Therefore, we used the hypergeometric distribution to calculate the statistical significance (p value) to see whether the observed number of the given TF-targeted genes was enriched in the specific pathway.

For manuscripts utilizing custom algorithms or software that are central to the research but not yet described in published literature, software must be made available to editors and reviewers. We strongly encourage code deposition in a community repository (e.g. GitHub). See the Nature Research guidelines for submitting code & software for further information.

Data

Policy information about availability of data

All manuscripts must include a data availability statement. This statement should provide the following information, where applicable:

- Accession codes, unique identifiers, or web links for publicly available datasets
- A list of figures that have associated raw data
- A description of any restrictions on data availability

Sequencing data have been deposited in Gene Expression Omnibus (GEO) under accession number GSE146697 with the URL https://www.ncbi.nlm.nih.gov/geo/query/acc.cgi.

Field-specific reporting

Please select the one below that is the best fit for your research. If you are not sure, read the appropriate sections before making your selection.

[x] Life sciences [ ] Behavioural & social sciences [ ] Ecological, evolutionary & environmental sciences

For a reference copy of the document with all sections, see nature.com/documents/nr-reporting-summary-flat.pdf

Life sciences study design

All studies must disclose on these points even when the disclosure is negative.

Sample size	Three independent infiltration were performed in the 3 host red kidney bean plants.
Data exclusions	No data were excluded from the analyses.
Replication	Biological or technical replicates were performed to ensure replication for HT-SELEX, RT-qPCR, and promoter activity detection experiments. Results for HT-SELEX were shown to be reproducible between replicates and DBD/FL from the same TF (Supplementary data 1). For RT-qPCR and promoter activity detection, the assays were repeated at least twice with similar results (as the error bar shows).
Randomization	There was no relevant randomization in this study.
Blinding	There was no relevant blindid in this study.

Reporting for specific materials, systems and methods

We require information from authors about some types of materials, experimental systems and methods used in many studies. Here, indicate whether each material, system or method listed is relevant to your study. If you are not sure if a list item applies to your research, read the appropriate section before selecting a response.

April 2020

Materials & experimental systems

n/a	Involved in the study	
☒	☐	Antibodies
☒	☐	Eukaryotic cell lines
☒	☐	Palaeontology and archaeology
☐	☒	Animals and other organisms
☒	☐	Human research participants
☒	☐	Clinical data
☒	☐	Dual use research of concern

Methods

n/a	Involved in the study	
☒	☐	ChIP-seq
☒	☐	Flow cytometry
☒	☐	MRI-based neuroimaging

Animals and other organisms

Policy information about studies involving animals; ARRIVE guidelines recommended for reporting animal research

Laboratory animals	The study did not involve laboratory animals. The strains we used are Pseudomonas savastanoi pv. phaseolicola 1448A, Escherichia coli DH5α and Escherichia coli BL21. The plant we used are red kidney bean.
Wild animals	The study did not involve laboratory animals.
Field-collected samples	The study did not involve laboratory animals.
Ethics oversight	The study did not involve laboratory animals. No ethical approval or guidance was required.

Note that full information on the approval of the study protocol must also be provided in the manuscript.
